# Characterization of the *FAD2* Gene Family in Soybean Reveals the Limitations of Gel-Based TILLING in Genes with High Copy Number

**DOI:** 10.3389/fpls.2017.00324

**Published:** 2017-03-13

**Authors:** Naoufal Lakhssassi, Zhou Zhou, Shiming Liu, Vincent Colantonio, Amer AbuGhazaleh, Khalid Meksem

**Affiliations:** ^1^Department of Plant, Soil and Agricultural Systems, Southern Illinois UniversityCarbondale, IL, USA; ^2^Department of Animal Science, Food and Nutrition, Southern Illinois UniversityCarbondale, IL, USA

**Keywords:** EMS mutagenesis, mutation breeding, forward genetics, reverse genetics, TILLING, oleic acid, *FAD2-1*, *FAD2-2*

## Abstract

Soybean seed oil typically contains 18–20% oleic acid. Increasing the content of oleic acid is beneficial for health and biodiesel production. Mutations in *FAD2-1* genes have been reported to increase seed oleic acid content. A subset of 1,037 mutant families from a mutagenized soybean cultivar (cv.) Forrest population was screened using reverse genetics (TILLING) to identify mutations within *FAD2* genes. Although no *fad2* mutants were identified using gel-based TILLING, four *fad2-1A* and one *fad2-1B* mutants were identified to have high seed oleic acid content using forward genetic screening and subsequent target sequencing. TILLING has been successfully used as a non-transgenic reverse genetic approach to identify mutations in genes controlling important agronomic traits. However, this technique presents limitations in traits such as oil composition due to gene copy number and similarities within the soybean genome. In soybean, *FAD2* are present as two copies, *FAD2-1* and *FAD2-2. Two FAD2-1* members: FAD2-1A and FAD2-1B; and three *FAD2-2* members: *FAD2-2A, FAD2-2B*, and *FAD2-2C* have been reported. Syntenic, phylogenetic, and *in silico* analysis revealed two additional members constituting the *FAD2* gene family: *GmFAD2-2D* and *GmFAD2-2E*, located on chromosomes 09 and 15, respectively. They are presumed to have diverged from other *FAD2-2* members localized on chromosomes 19 (*GmFAD2-2A* and *GmFAD2-2B*) and 03 (*GmFAD2-2C*). This work discusses alternative solutions to the limitations of gel-based TILLING in functional genomics due to high copy number and multiple paralogs of the *FAD2* gene family in soybean.

## Introduction

The fatty acid desaturase-2 enzyme (FAD2-1) is responsible for the conversion of oleic acid to linoleic acid in the developing soybean seeds (Okuley et al., 1994; Schlueter et al., 2007). It has been reported that five *FAD2* members are present in four different loci and constitute the fatty acid desaturase family in the soybean genome (Schlueter et al., 2007; Bachlava et al., 2008; Pham et al., 2010; Zhang et al., 2014). These members belong to two *FAD2* copies (*FAD2-1* and *FAD2-2*), present with many splicing variants and a strong conservation of gene sequence, order, and orientation (Heppard et al., 1996; Schlueter et al., 2006, 2007). FAD2 variants have diversified functionalities in fatty acid modification: catalyzing hydroxylation (van de Loo et al., 1995; Broun and Somerville, 1997), epoxidation (Lee et al., 1998), formation of acetylenic bonds (Voinnet et al., 2003), and conjugated double bonds (Cahoon et al., 1999; Dyer et al., 2002; Cahoon and Kinney, 2004). Interestingly, many multifunctional FAD2 enzymes have been characterized. These include the bifunctional hydroxylase/desaturase activity from *Lesquerella fendleribroun* (Broun et al., 1998), and trifunctional acetylenase from *Crepis alpina*, which catalyzes the formation of both *trans* and *cis* double bonds at the Δ12 position of oleic acid (Carlsson et al., 2004). Although *FAD2-2* members have not been linked to oleic acid content, *FAD2-1* has been reported to control the high oleic acid content of certain soybean lines (Hoshino et al., 2010; Pham et al., 2010, 2011).

Downregulation of *FAD2-1A* using a ribozyme-terminated antisense approach presented major limitations (Buhr et al., 2002). In addition, the production and exportation of these transgenic high oleic acid lines have to overcome regulatory hurdles in the United States' most important exportation markets. Therefore, producing EMS mutants with a single base pair mutation within these two candidate genes appears to be the best strategy to produce beneficial alleles with high oleic content.

Three sources of *FAD2-1A* mutants were previously described: M23 carrying a large genomic deletion of 160 Kb; KK21, a mutant with a single nucleotide deletion; and 17D (S117N) were combined with one *FAD2-1B* mutant (P137R) from PI283327 to produce high oleic acid lines (Dierking and Bilyeu, 2009; Hoshino et al., 2010; Pham et al., 2010; Bolon et al., 2011). However, many of these lines presented poor agronomic characteristics such as a reduction in oleic acid content when grown in cooler environments (17D), a reduction in yields, and/or susceptibility to pathogens (i.e., SCN, SDS, etc.) (Taylor et al., 2002; Anai et al., 2008; Pham et al., 2010). The M23-derived lines were shown to have a reductions in yield that appear to be linked to the deleted portion of chromosome 10 (Taylor et al., 2002). Thus, developed mutants like *FAD2-1A* from the M23 line with a large genomic deletion is agronomically impractical. Consequently, producing new EMS mutants with a single base pair mutation in each *FAD2-1* gene seems to be a better strategy to produce high oleic mutant alleles with stable oil content, particularly after combining both mutant alleles into one line (Pham et al., 2011). This may result in soybean lines with more than 80% oleic acid content, as it was reported in two previous independent studies (Hoshino et al., 2010; Pham et al., 2011). Since then, several efforts have been accomplished to improve soybean seed composition traits (Dierking and Bilyeu, 2009; Pham et al., 2012; Lestari et al., 2013; Chaudhary et al., 2015).

TILLING is a useful method for the identification of mutations in a certain gene, such as those controlling important agronomic traits. TILLING has been commonly employed in many organisms (McCallum et al., 2000; Meksem et al., 2008; Moens et al., 2008; Dierking and Bilyeu, 2009; Colasuonno et al., 2016; Gauffier et al., 2016; Nida et al., 2016). To perform TILLING, a mutagenized population is developed using a chemical mutagen such as ethyl methanesulfonate (EMS). Next, a mismatch enzyme that recognizes single base mutations is used to identify particular mutants within a given population.

Two whole genome duplication events occurred in soybean 13 and 59 million years ago (Schmutz et al., 2010). This was followed by subsequent divergent selection in many gene families (Yin et al., 2013; Zhu et al., 2014; Singh and Jain, 2015). Nearly 75% of predicted genes in soybean are present in multiple copies due to, in part, those two duplication events (Schmutz et al., 2010). Interestingly, the results obtained from syntenic, phylogenetic, and *in silico* analysis demonstrated that the *FAD2* gene family contains seven members; two *FAD2-1* and five *FAD2-2* paralogs, but not three *FAD2-2* members as have been previously reported (Schlueter et al., 2007; Pham et al., 2010; Zhang et al., 2014). The newly identified members are on chromosomes 09 (*GmFAD2-2D*) and 15 (*GmFAD2-2E*), and the *FAD2-2E* may have recently diverged together with the other *FAD2-2* members localized on chromosomes 19 (*GmFAD2-2A* and *GmFAD2-2B*) and 03 (*GmFAD2-2C*); however, in the phylogenetic analysis the *FAD2-2D* was separately grouped from the rest of the *FAD2-2* gene family.

A soybean (*Glycine max*) EMS mutagenized population employing the cultivar “Forrest” was used to identify soybean lines carrying mutations in both the *FAD2-1A* and *FAD2-1B* genes by reverse genetic and forward genetic approaches. Using gel-based TILLING, no mutant was identified within the *FAD2-1A* and *FAD2-1B*, however, a forward genetic approach based on gas chromatography screening identified five soybean lines with high oleic acid seed content. Through target sequencing, new alleles presenting mutations in either the *FAD2-1A* or *FAD2-1B* were identified.

## Methods

### Phylogenetic analysis

Multiple sequence alignments were performed using the MEGA4 software package and the Clustal W algorithm. An unrooted phylogenetic tree was calculated with the neighbor-joining method (Saitou and Nei, 1987), and tree topology robustness was tested through bootstrap analysis of 5,000 replicates.

### Development of an EMS mutagenized “Forrest” population

The soybean cv. Forrest seed was acquired from the Southern Illinois University Carbondale Agricultural Research Center (ARC) and was used to develop an EMS-mutagenized population. The seed was mutagenized with 0.6% (v/v) EMS, as described by Meksem et al. (2008) and planted at the Horticulture Research Center (HRC). A total of 1,588 M2 family seeds were harvested in 2011, then successively advanced to an M3 generation in 2012 and to an M4 generation in 2013, all at Southern Illinois University Carbondale. In total, 1,037 mutant families (lines) of M3/M4 were harvested. Then, seeds were threshed at the ARC, packaged, and stored at 4°C for short term storage and at −20°C for long term storage.

### Mutation screening of *FAD2-1A* and *FAD2-1B*

Specific primers for *FAD2-1A* and *FAD2-1B* were used to screen the new population of EMS-mutagenized M3 families from the SCN-resistant *cv*. Forrest (Table S4). For target sequencing, two pairs of primers were used to sequence the promoters and the exons on both *FAD2-1A* and *FAD2-1B*, and TILLING was performed on a LICOR system as previously described (Meksem et al., 2008). Then, new alleles and subsequent amino acid changes within the predicted protein sequences were identified.

### Analysis of seed fatty acids

All M3 and M4 lines were analyzed for seed fatty acid composition using the two-step procedure as outlined by Kramer et al. (1997). At least four seeds per mutant from the M3 and M4 generations and 30 wild type Forrest seeds were randomly selected for fatty acid analysis. Briefly, each seed was broken/scratched and then loaded into one 16 mm × 200 mm tube with a Teflon-lined screw caps. Two milliliters of sodium methoxide was added into each tube and then the tube was incubated in a water bath at 50°C for 10 min. Afterward 3 ml of 5% (v/v) methanolic HCl was added into each tube after the tube was cooled for 5 min. Subsequently, the tube was incubated in a water bath at 80°C for 10 min, cooled for 7 min, and then 7.5 ml of 6% (w/v) potassium carbonate and 1 ml of hexane were added. Finally, the tubes were centrifuged at 1,200 g for 5 min to separate the layers. The upper layer was then transferred and used for the fatty acid analyses using a Shimadzu GC-2010 (Columbia, MD) gas chromatograph equipped with a flame ionization detector and a Supelco 60-m SP-2560 (Bellefonte, PA) fused silica capillary famewax column (0.25 mm i.d. × 0.25 μm film thickness). The helium carrier gas was maintained at a linear velocity of 23 cm/s. The oven temperature was programmed for 175°C for 22 min, then increased at 15°C/min to 225°C and held for 3 min. The injector and detector temperatures were set at 255°C. Peaks were identified by comparing the retention times with those of the corresponding standards (Nu-Chek-Prep., Elysian, MN; Supelco). The levels of palmitic acid, stearic acid, oleic acid, linoleic acid, and linolenic acid were calculated using the statistical computing package (JMP Pro V12 software).

### Detection of genotypes and mutations by target sequencing

Young leaf tissue from mutants with high oleic acid content, as well as wild type Forrest, was used to extract DNA using the CTAB method (Rogers and Bendich, 1994) with small modifications. Specific primers were designed to amplify the fragments covering both the promoters and exons of two genes, *FAD2-1A* and *FAD2-1B*, using the extracted DNA as the template with 38 cycles of PCR amplification at 94°C for 30 s, 52°C for 30 s, and 72°C for 1 min. The PCR products were purified by enzymatic clean-up, or the specific bands were cut from the agarose gels after electrophoresis using a Qiagen gel extraction kit (QIAquick). Then, the purified PCR fragments were sequenced at GENEWIZ (www.genewiz.com). The genotypes and specific mutations were determined by comparing the genomic cDNA and predicted protein sequences of the genes with wild type Forrest using DNASTAR Lasergene software.

### Alignment analysis of FAD2

In order to evaluate the position of substitutions, multiple sequence alignments were performed with the ClustalW program and mutations were overlaid on the wild type Forrest nucleotide and protein sequences. Predicted protein alignment of the FAD2-1A and FAD2-1B sequences of the wild type Forrest and the identified missense mutants were obtained using DNASTAR Lasergene 8 software package and the Clustal W algorithm. Alignment analysis of the seven soybean FAD2 family members was performed using the same tools described previously. All parameter values correspond to default definitions.

### Statistical analysis

All of the results presented were performed with the analysis of variance by T-student test means comparison using JMP Pro V12 software.

## Results

### *FAD2* duplication in the soybean genome

Five fatty acid desaturase (*GmFAD2*) gene family members were previously reported to be located on chromosomes 10 (*GmFAD2-1A*; Glyma.10G278000), 20 (*GmFAD2-1B*; Glyma.20G111000), 19 (*GmFAD2-2A*; Glyma.19G147300), 19 (*GmFAD2-2B*; Glyma.19G147400), and 03 (*GmFAD2-2C*; Glyma.03G144500) (Schlueter et al., 2007; Pham et al., 2010; Zhang et al., 2014). The analysis of the *G. max*_[Williams 82]_ genome using both soybase and phytozome databases indicates that two novel *FAD2-2* members were found on chromosomes 09 (Glyma.09G111900) and 15 (Glyma.15G195200) and named in this study as *GmFAD2-2D* and *GmFAD2-2E*, respectively (Table 1, Figures S7, S9). In particular, *GmFAD2-1A* and *GmFAD2-1B* present the seed specific isoforms in soybean with very high expression levels in the seeds (Figure S1).

**Table 1:** *****FAD2*** gene family isoforms with corresponding copy number, paralogs, and splicing variants on the soybean genome**.

**Copies**	**Gene/paralogs**	**Splicing variants**	**Old gene ID (V1.1)**	**Amino acid sequence**	**Score**	**Similarity (%)**
*FAD2-1*	*FAD2-1A*	Glyma.10G278000.1	Glyma10g42470.1	387	–	–
		Glyma.10G278000.2	Glyma10g42470.2	387	2,551	100.00
	*FAD2-1B*	Glyma.20G111000.1	Glyma20g24530.1	387	2,429	97.70
		Glyma.20G111000.2	Glyma20g24530.2	387	2,429	97.70
		Glyma.20G111000.3	Glyma20g24530.3	387	2,391	95.90
		Glyma.20G111000.4	Glyma20g24530.4	387	2,429	97.70
		Glyma.20G111000.5	Glyma20g24530.5	387	2,429	97.70
		Glyma.20G111000.6	Glyma20g24530.6	387	2,429	97.70
		Glyma.20G111000.7	Glyma20g24530.7	387	2,429	97.70
*FAD2-2*	*FAD2-2C*	Glyma.03G144500.1	Glyma03g30070.1	383	–	–
		Glyma.03G144500.2	Glyma03g30070.2	383	2,471	100.00
	*FAD2-2B*	Glyma.19G147400.1	Glyma19g32940.1	383	2,373	97.40
		Glyma.19G147400.2	Glyma19g32940.2	383	2,373	97.40
	*FAD2-2D*	Glyma.09G111900.1	Glyma09g17170.1	368	1,868	84.30
	*FAD2-2E*	Glyma.15G195200.1	Glyma.15G23200.1	293	1,829	75.70
	*FAD2-2A*	Glyma.19G147300.1	Glyma.19G32930.1	216	1,164	52.00

*Scores and similarities based on FAD2-1A (Glyma.10G278000.1) and FAD2-2C (Glyma.03G144500.1) were obtained from phytozome database*.

**Figure 1:**
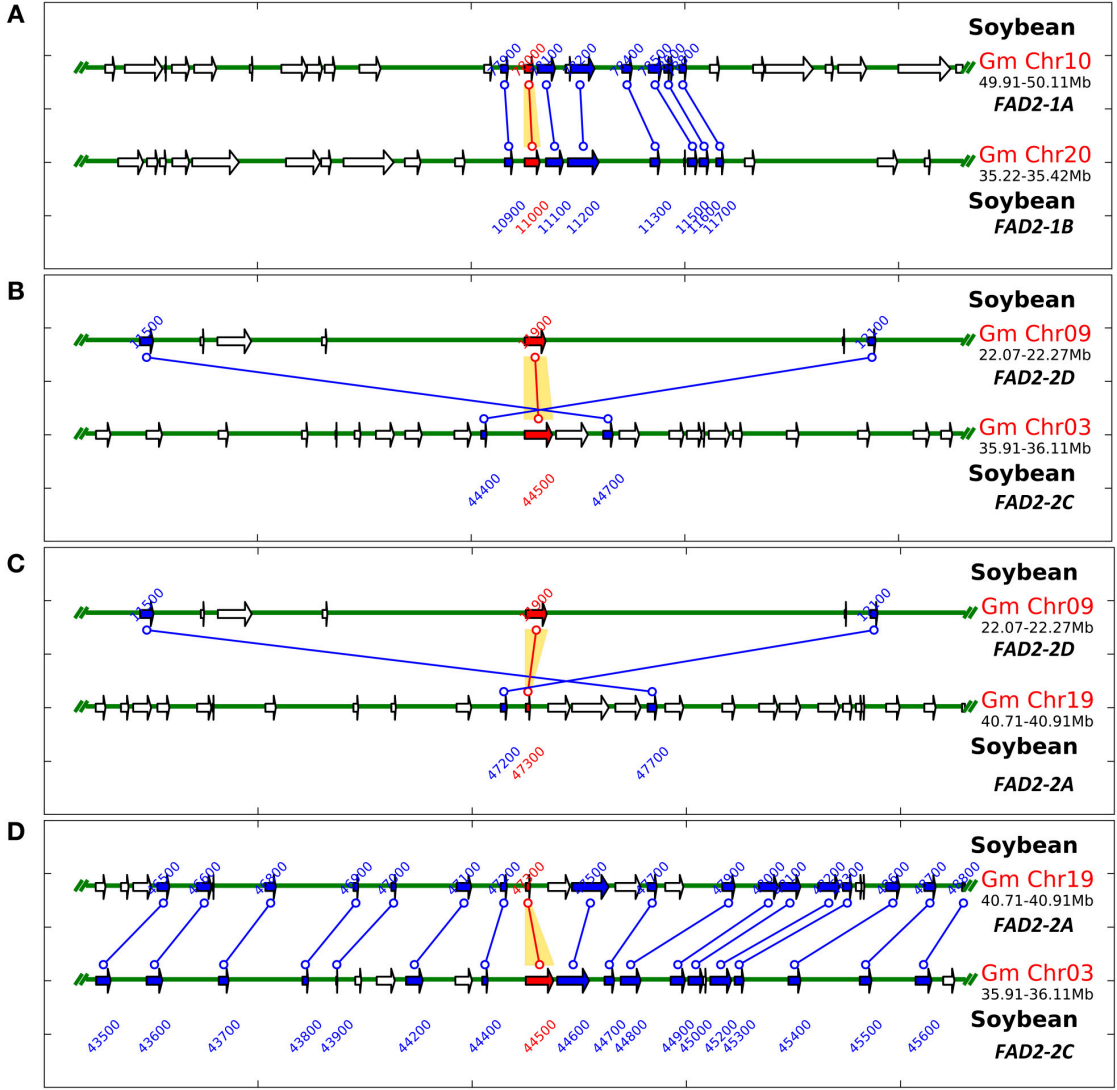
**Schematic representation of ***GmFAD2*** containing duplicated segments identified in the soybean genome**. Soybean *FAD2* intragenome syntenic relationships were calculated using the Plant Genome Duplication Database (http://chibba.agtec.uga.edu/duplication/). **(A)** *GmFAD2* in chr10 and chr20 belongs to another duplicated segment with less conserved genes; just 8 duplicated genes or anchors. **(B)** *GmFAD2* in chr03 and chr09 belongs to a third duplicated segment containing 19 additional inverted duplicated genes or anchors. **(C)** *GmFAD2* in chr19 and chr09 belongs to a third duplicated segment containing 21 additional inverted duplicated genes or anchors. **(D)** *GmFAD2* in chr19 and chr03 belongs to a very large duplicated segment containing 1,060 additional conserved duplicated genes or anchors. Graphs represent ±100 kb duplicated region centered in the *GmFAD2* gene.

In order to test the contribution of the soybean segmental duplications in the number of *GmFAD2* genes, the soybean genome was analyzed for duplicated chromosomal segments containing *GmFAD2s* using the plant genome duplication database (Tang et al., 2008a,b; Lee et al., 2012). As shown in Figure 1, four independent duplicate blocks containing the genomic pairs *FAD2-2A/FAD2-2C, FAD2-2A/FAD2-2D, FAD2-2D/FAD2-2C*, and *FAD2-1A/FAD2-1B* were identified in ±100 kb duplicated regions centered around the *GmFAD2* genes, with *FAD2-2D* constituting a new member of the *GmFAD2* gene family. The intragenome syntenic relationship calculations for *GmFAD2*s in all conserved genes surrounding *GmFAD2* reveal that *GmFAD2* duplication between chr19/chr03 belongs to a very large duplicated segment containing at least 1,060 additional conserved duplicated genes or anchors (Table S1). However, *GmFAD2* duplication between chr19/chr09, chr03/chr09, and chr10/chr20 belongs to other duplicated segments with less conserved genes containing 21 inverted, 19 inverted, and 8 non-inverted duplicated genes or anchors, respectively (Tables 2–**4**). Interestingly, syntenic analysis did not show any duplicated block or segment between all four *FAD2* members cited previously and the *FAD2-2B* and *FAD2-2E* members in soybean. Surprisingly, *FAD2-2B* was contained within two duplicated blocks containing 7 and 8 additional conserved duplicated genes or anchors each with oil palm (*Elaeis guineensis*) (Figure S2). In addition, FAD2-2A showed a truncated protein and shared the lowest identity with FAD2-2C, showing only 52% as compared to the other members; GmFAD2-2B, GmFAD2-2D, and GmFAD2-2E that shared 97.40, 84.30, and 75.70% identities, respectively (Table 1).

**Table 2:** **Gene divergence of duplicated regions between Chr10 (49.91–50.11 Mb) and Chr20 (35.22–35.42 Mb)**.

**Order within block**	**Locus 1**	**Locus 2**	**Putative function**	**Ka**	**Ks**	**Ka/Ks**
1	Glyma.10G277900	Glyma.20G110900	UF, ATPase inhibitor protein (*M. truncatula*)	0.09	0.13	0.69
**2**	**Glyma.10G278000**	**Glyma.20G111000**	**Fatty acid desaturase**	**0.03**	**0.13**	**0.23**
3	Glyma.10G278100	Glyma.20G111100	SNARE PROTEINS	0.15	0.31	0.48
4	Glyma.10G278200	Glyma.20G111200	COATOMER SUBUNIT DELTA	0.01	0.07	0.14
5	Glyma.10G278400	Glyma.20G111300	Hemerythrin HHE cation binding domain	0.03	0.15	0.2
6	Glyma.10G278500	Glyma.20G111500	Ribonuclease HI	0.43	0.51	0.84
7	Glyma.10G278600	Glyma.20G111600	UF, Unkown Function	0.08	0.12	0.66
8	Glyma.10G278800	Glyma.20G111700	EPLICATION PROTEIN A 14 KDA SUBUNIT A	0.02	0.06	0.33

*GmFAD2 in chr10 and chr20 belongs to another duplicated segment with less conserved genes; just 8 duplicated genes or anchors. Analysis represent ±100 kb duplicated region centered in the GmFAD2 gene*.

### Evolution of *FAD2* gene family members

**Figure 2:**
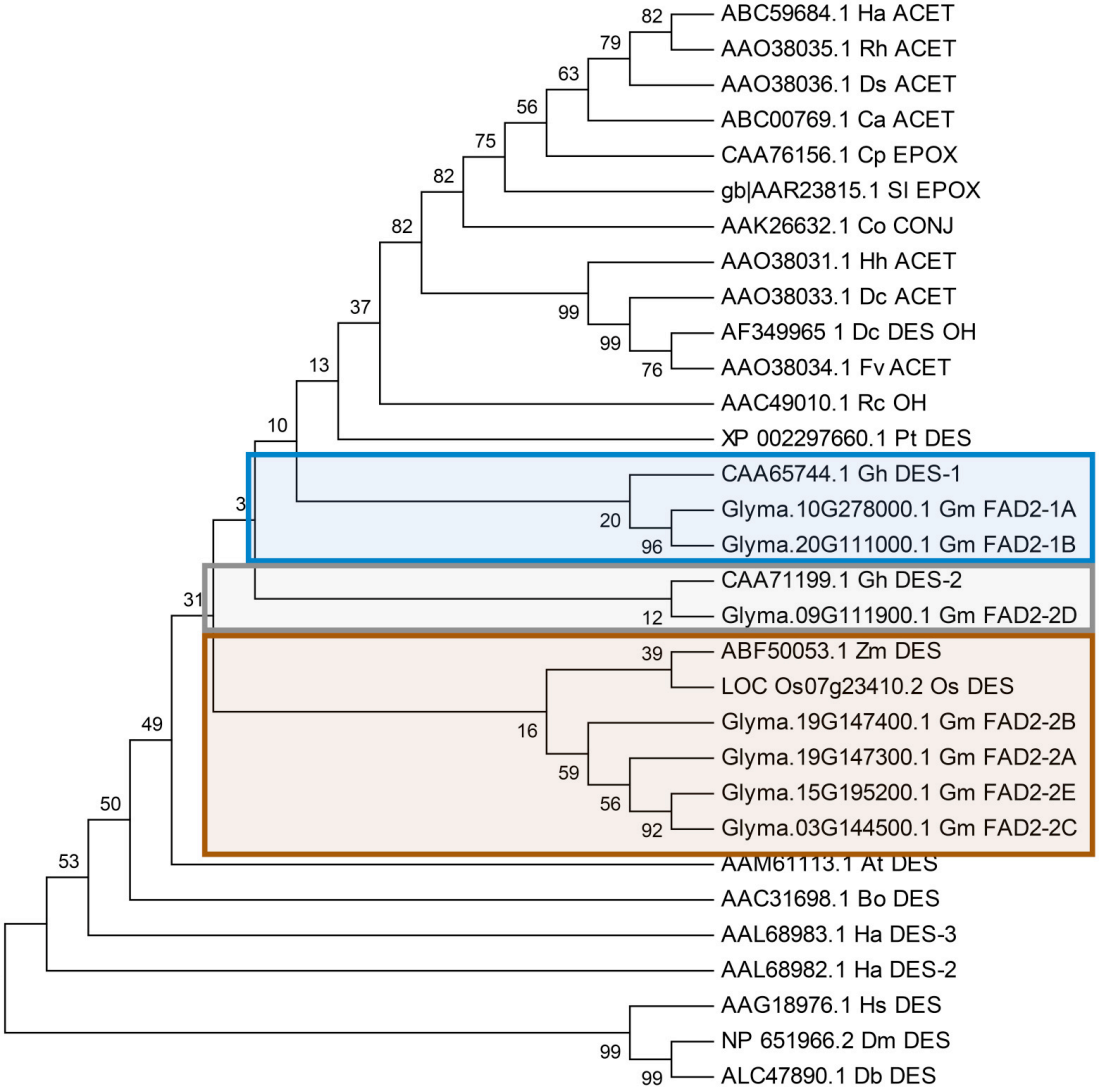
**Phylogenetic tree comparison of seven soybean ***GmFAD2*** gene family members and orthologous FAD2s from 20 other species**. FAD2 proteins identified in four model species from *O. sativa* (Monocots), *A. thaliana* (Dicots), *D. melanogaster* (Insects), and *H. salinarum* (Archaea) were included in the phylogenetic analysis. FAD2 with different functions such as desaturases (DES), hydroxylases (OH), epoxygenases (EPOX), acetylenases (ACET), and conjugases (CONJ) were included in the analysis. At, *Arabidopsis thaliana*; Co, *Calendula officinalis*; Ha, *Helianthus annuus*; Rh, *Rudbeckia hirta*; Ds, *Dimorphotheca sinuate*; Ca, *Crepis alpine*; Cp, *Crepis palaestina*; Sl, *Stokesia laevis*; Dc, *Daucus carota*; Fv, *Foeniculum vulgare*; Hh, *Hedera helix*; Bo, *Borago officinalis*; Gh, *Gossypium hirsutum*; Pt, *Populus trichocarpa*; Rc, *Ricinus communis*; Zm, *Zea mays*; Os, *Oriza sativa*; Hs, *Halobacterium salinarum*; Db, *Drosophila busckii*; Dm, *Drosophila melanogaster*. The phylogenetic tree was generated using the MEGA4 software package and the Clustal W algorithm, and calculated using the neighbor-joining method. The tree bootstrap values are indicated at the nodes (*n* = 5,000).

To elucidate the phylogenetic relationship of soybean *FAD2* gene family members, the seven predicted polypeptide sequences of soybeans were aligned with orthologous FAD2 sequences from monocots, dicots, insects, and archaea. A neighbor-joining tree was constructed using Mega 4 software (Saitou and Nei, 1987). The analysis grouped FAD2-1 and FAD2-2 members separately, which supports the results obtained from the previous syntenic analysis. As expected, the FAD2 from insects (DmFAD2 and DbFAD2) and archaea (HsFAD2) were outgrouped. As shown in Figure 2, GmFAD2-1A and GmFAD2-1B are aligned next to each other and to other seed-expressed fatty acid desaturases such as cotton GhFAD2-1. However, GmFAD2-2A, -2B, -2C, and -2E formed a new branch, most likely to have recently diverged. Interestingly, it is embedded in a clade with the two FAD2 from the monocots corn ZmFAD2 and rice OsFAD2. The newly identified GmFAD2-2D member was separately grouped from GmFAD2-1 and the other four GmFAD2-2 members, and was aligned with expressed fatty acid desaturases GhFAD2-2 from cotton. None of the seven FAD2 members were grouped with functionally divergent fatty acid modifying enzymes such as fatty acid hydroxylases, acetylenases, epoxygenases, and/or conjugases.

### Limitations of a gel-based TILLING approach due to copy number

**Figure 3:**
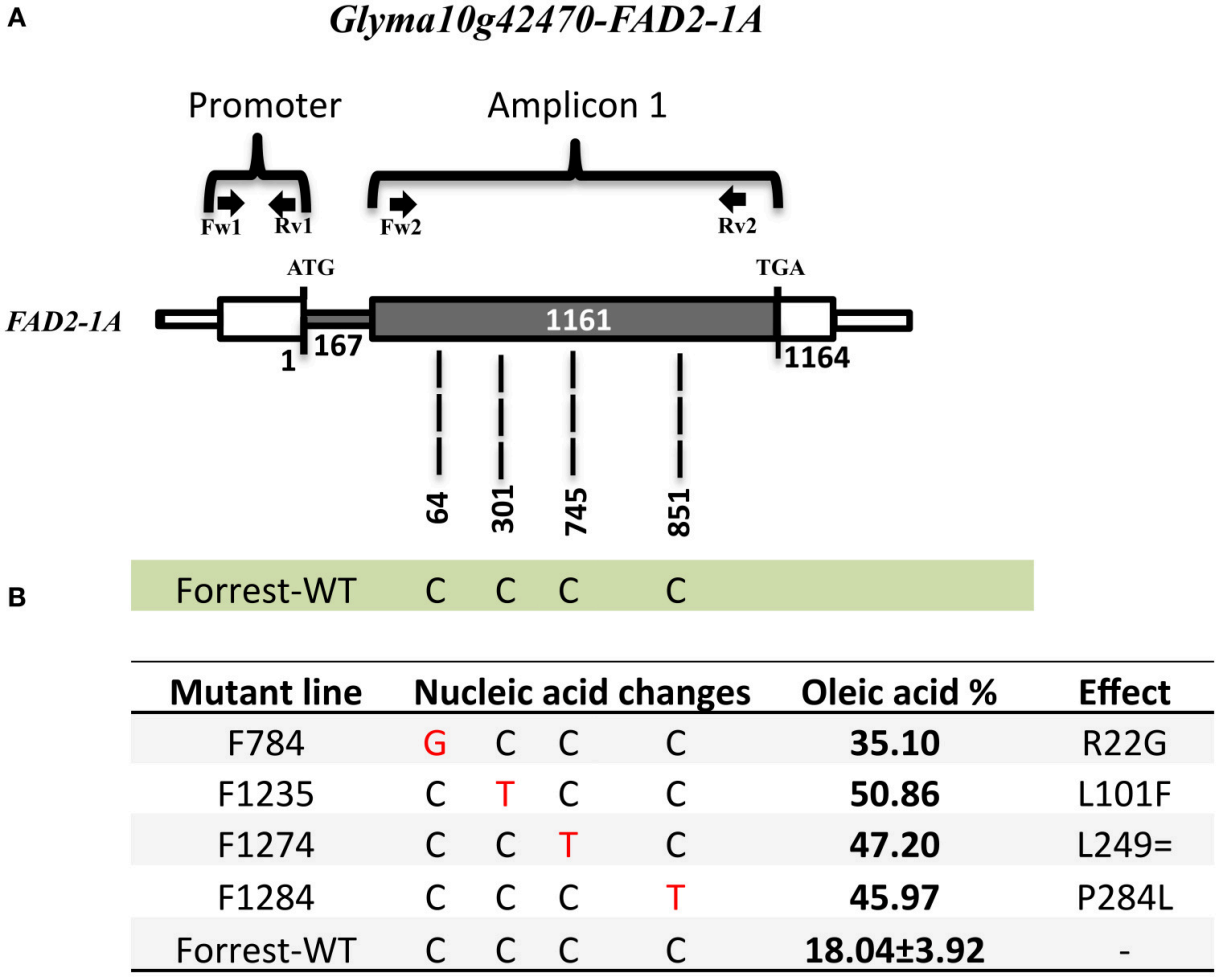
*****FAD2-1A*** mutations of the screened four high oleic acid mutants. (A)** *FAD2-1A* gene model in the wild type Forrest. **(B)** Predicted FAD2-1A protein showing amino acid differences between the Forrest-WT and the four high oleic mutants identified by forward genetics, three missense mutations (R22G, L101F, and P284L), and one silent mutation (L249=) within the *FAD2-1A*. The oleic acid level represents the highest content obtained from each line. The primers used for TILLING and gDNA sequencing are indicated by arrows.

**Figure 4:**
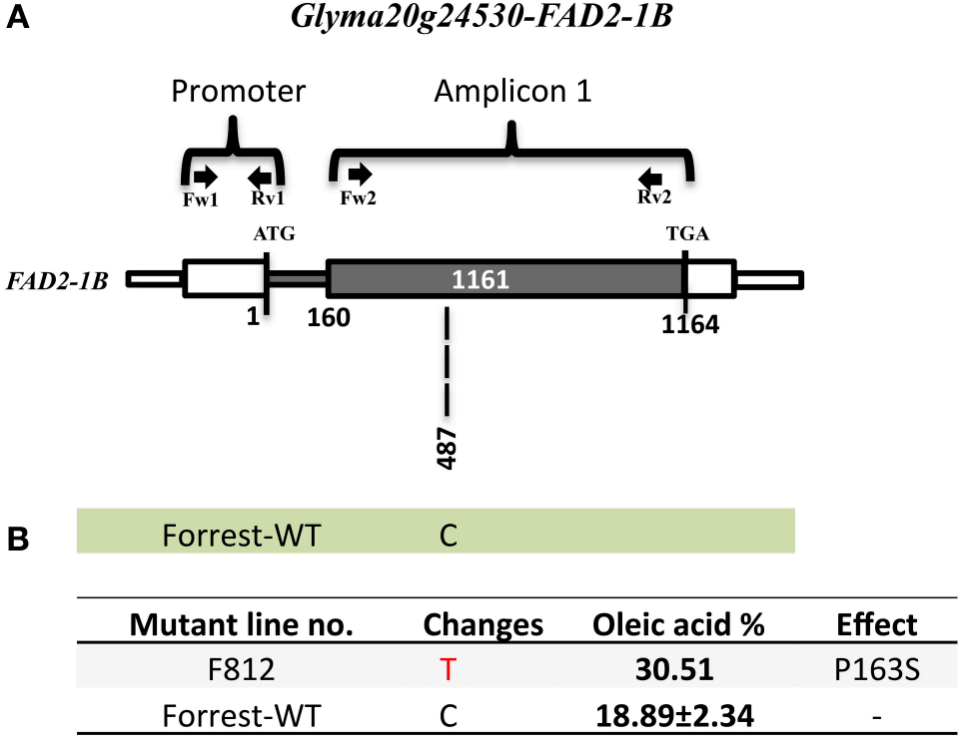
*****FAD2-1B*** mutations of the screened high oleic acid mutant. (A)** *FAD2-1B* gene model in wild type Forrest. **(B)** Predicted FAD2-1B protein showing amino acid differences between the Forrest-WT and the high oleic mutant identified by forward genetics; one missense mutations (P163S) within the *FAD2-1B*. The oleic acid level represents the highest content obtained. The primers used for PCR genotyping are indicated by arrows.

Fatty acid desaturases in the soybean genome are present under two *FAD2* copies: *FAD2-1* and *FAD2-2*, with seven *FAD2* members in six linkage groups of the soybean genome (Table 1). In addition, *FAD2-1A, FAD2-2B*, and *FAD2-2C* have two splicing variants and *FAD2-1B* has 7 splicing variants that produce many isoforms (Table 1, Figures S8, S9). Wild type Forrest seeds were used as a background and mutagenized with a treatment of 0.6% (v/v) EMS. In 2011, 1,588 mutant families of the M2 Forrest population were developed and advanced to the M3 generation in 2012 and the M4 generation in 2013, with a total of 1,037 mutant families each. The EMS-mutagenized Forrest M2 populations were screened for mutations within *FAD2-1A* and *FAD2-1B* using gel-based TILLING. Two pairs of primers covering both the promoters and the exons for each of the two genes were designed to identify mutants in *FAD2-1A* (Figure 3A) and *FAD2-1B* (Figure 4A). Interestingly, pooling the heteroduplexes during TILLING and using mismatch enzymes resulted in detection of many mutants from the gel (Figure S3), however, all of them corresponded to false positive mutants due to non-specific hybridizations. After sequencing all these lines, no mutants were identified in either of the two-targeted genes using this reverse genetic approach after screening more than 2,000 mutant families belonging to the FM2-2011, in addition to the FM2-2008 EMS mutagenized population (Meksem et al., 2008; Liu et al., 2012).

### Identification of soybean lines with high level of seed oleic acid content by forward genetic screening

The seed fatty acid content of all the M3 and M4 lines was measured by gas chromatography, and compared to the wild type Forrest. The graphs in Figure S4 represent the distribution of the five major fatty acid contents in the seed oil of the Forrest M3 mutant population. The distribution of the five major seed fatty acid contents ranged between 2.05 and 31.89% for palmitic acid, 0.41 and 11% for stearic acid, 3.22 and 47.24% for oleic acid, 8.86 and 84.95% for linoleic acid, and 1.77 and 16.31% for linolenic acid, as shown in Figure S4. However, the average of the Forrest wild type was 10.3% for palmitic acid, 3.25% for stearic acid, 18.89% for oleic acid, 53.71% for linoleic acid, and 6.89% for linolenic acid.

**Figure 5:**
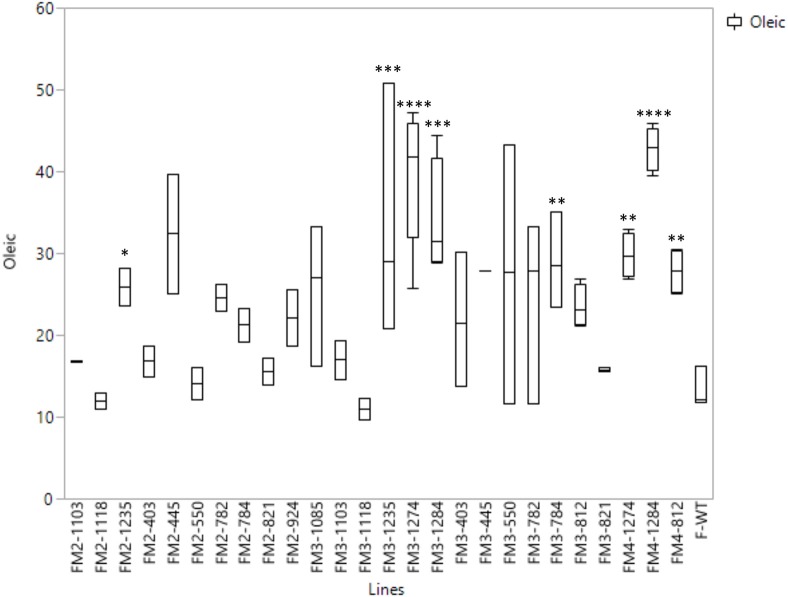
**Box plots of oleic acid content of the seed oil of the wild type Forrest and 14 selected re-screened mutants in M2 (2011), M3 (2012), and/or M4 generations**. Fatty acid levels were averaged for n segregating M2, M3, and M4 lines for each mutation. Asterisks indicate significant differences between samples as determined by *t*-test (^*^P < 0.0001, ^**^P < 0.001, ^***^P < 0.01, ^****^P < 0.05).

The highest oleic acid content in the seed oil of the Forrest M3 mutant population was 47.24%, while the average of oleic acid content of the wild type Forrest was 18.89%. This increase presented about 2.5 times the level of oleic acid contained in the wild type Forrest seed. Using forward genetic screening, 1,037 M3 mutants were screened for fatty acid content. A subset of 14 lines presenting high oleic acid content in the M3 population was selected. These lines presented at least 1.5 times the levels of seed oleic acid contained in the wild type Forrest. However, only three mutant lines: F812, F1274, and F1284, maintained a significantly high oleic acid content with a *P*-value of *P* < 0.01 in the M4 generation (Figure 5). The average of oleic acid content in the seed oil of F1274, F1284, and F812 was 39.5% (*P* < 0.01), 42.81% (*P* < 0.0001), and 27.82% (*P* < 0.01), respectively (**Table 5**). In addition to oleic acid; palmitic, stearic, linoleic, and linolenic acid contents were measured. The content of these four types of fatty acids in seed oil did not show significant differences between the three-screened mutants and the wild type Forrest, except for a decrease in linoleic acid (**Table 5**).

### Correlation between elevated oleic acid in soybean seeds and the *FAD2* genes

The enzyme FAD2 converts oleic acid to linoleic acid (Figure S5). In the soybean genome, there are two seed-specific *FAD2* genes, *FAD2-1A* and *FAD2-1B* (Table 1). In order to identify possible mutations in the *FAD2* genes and to test the direct correlation between the *FAD2* mutations and seed oleic acid phenotype alterations, the genomic sequences of the two *FAD2-1* isoforms of the three mutants (F1274, F1284, and F812) and the wild type Forrest were analyzed by target sequencing.

**Figure 6:**
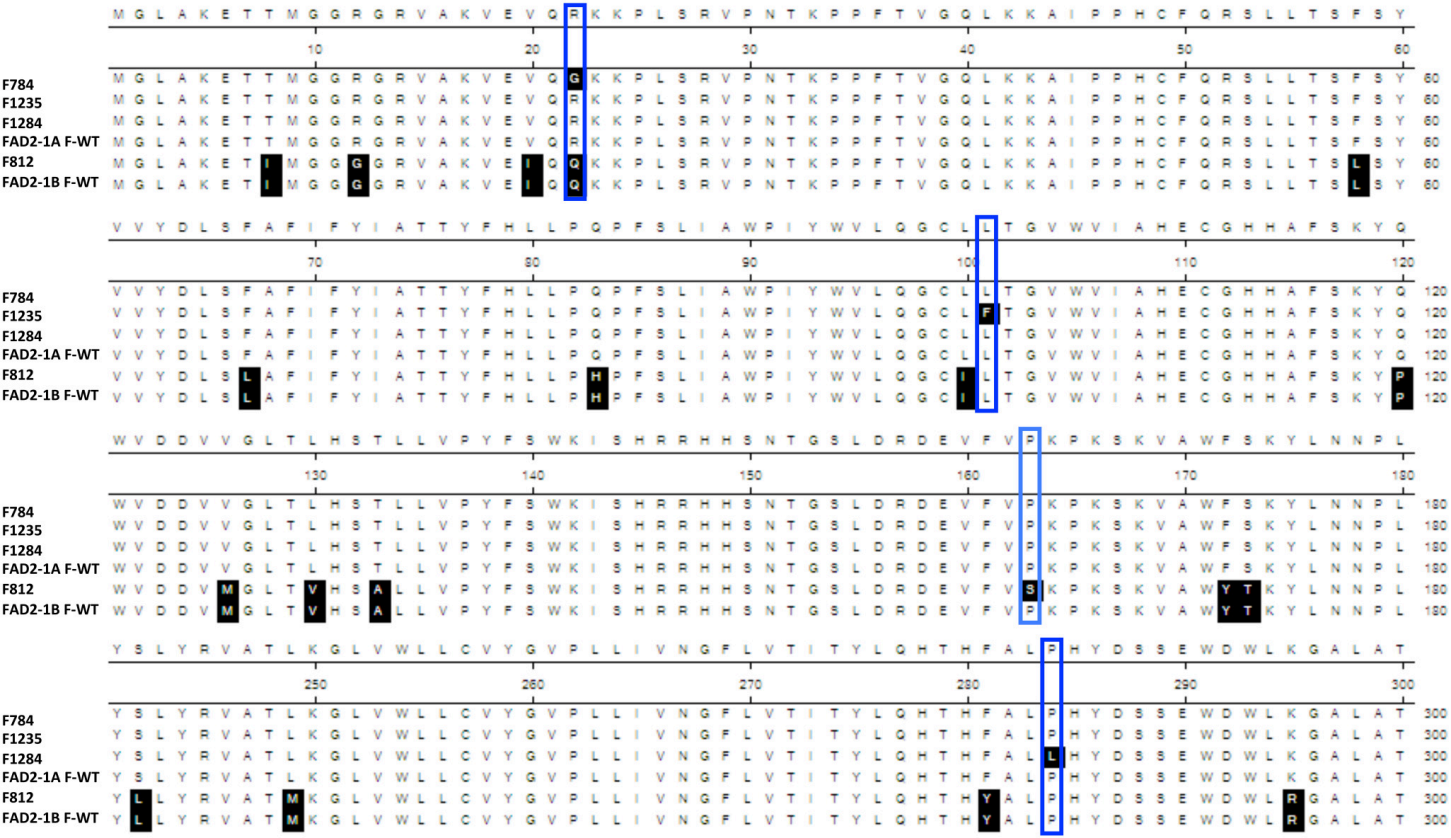
**FAD2 mutations of the four screened oleic acid mutants**. Protein sequence alignement of FAD2-1A, FAD2-1B, wild type Forrest, and the four screened missense mutants showing the identified substitutions: F784 (FAD2-1A_R22G_), F1235 (FAD2-1A_F101L_), F1284 (FAD2-1A_P284L_), and F813 (FAD2-1B_P163S_). Only sequences containing amino acid changes are represented, and thus, sequences from 181 to 240 are not shown.

The sequencing results showed that the two mutants F1274 and F1284 presented different SNPs at positions C745T and C851T in *FAD2-1A* compared to the Forrest wild type (Figure S10). Only one SNP generated amino acid changes, FAD2-1A_P284L_ in the mutant F1284 (Figures 3B, 6). In addition, a silent mutation, FAD2-1A_L249 =_, appeared in the F1274 mutant (Figures 3B, 6). However, neither of these two mutants exhibited a mutation in the *FAD2-1B* exons or promoter. Interestingly, sequencing results of the F812 mutant showed that it does present a SNP change (C487T) in *FAD2-1B* with amino acid changes FAD2-1B_P163S_ (Figures 4B, 6). No SNPs were found in the *FAD2-1A* exons or promoter for this F812 mutant. Our results indicated that the high oleic acid mutants exhibited missense mutations (new alleles) in *FAD2-1A* and *FAD2-1B*, seemingly correlating with the oleic acid phenotype alterations.

### Identification of additional *FAD2-1A* alleles with missense mutations by re-screening the M2 generation

One seed from each of the 1,037 M3 mutant families was screened for fatty acid content. A set of 14 lines within the M3 population with high oleic acid content was selected. However, only three mutant lines: F812 (FAD2-1B_P163S_), F1274 (FAD2-1A_L249 =_), and F1284 (FAD2-1A_P284L_), characterized previously maintained a significantly high oleic acid content in the M4 generation. The remaining 11 mutants showed no oleic acid content changes. The results of fatty acid analysis showed that three M3 lines were segregating for their oleic acid content (Table S2). The M3 mutants were then advanced to the M4 generation by planting one seed from each one of the 1,037 mutant families. It is likely that the selected seed from each of the 11 M3 lines that were identified to have high oleic acid were segregating for the non-mutant alleles when advanced to the M4 generation. For this reason, they did not present a higher oleic acid content. In order to support this hypothesis, another two seeds from each of these 11 mutant M2 and M3 families were re-screened when available. The fatty acid screening showed that two mutants from both the M2 and M3 generations maintained a high oleic acid content at least 1.5 times more than the level of oleic acid contained in the wild type Forrest (**Table 6**). Six mutants presented higher oleic acid content in either the M2 or M3 generations, while the other three mutants did not show any significant changes in oleic acid content (Table S3). In addition, only the two mutants F784 (*P* < 0.01) and F1235 (*P* < 0.05) maintained significantly higher seed oleic acid content in both M2 and M3 generations (Figure 5). These 11 mutants were also genotyped for both *FAD2-1* paralogs. Interestingly, target sequencing revealed that only the two mutants maintaining significantly higher content from both the M2 and M3 generations are new alleles in *FAD2-1A* presenting two missense mutations: FAD2-1A_R22G_ and FAD2-1A_L101F_, respectively (Figures 3B, 6).

## Discussion

### Limitations of gel-based TILLING in genes with high copy number and multiple paralogs of the *FAD2* gene family

The purpose of this study was the identification of soybean lines with high oleic acid levels, by screening the developed EMS mutagenized Forrest library using a gel-based TILLLING approach. We developed several soybean EMS mutagenized populations and screened multiple soybean mutants using TILLING during the past 10 years (Cooper et al., 2008; Meksem et al., 2008; Liu et al., 2011, 2012). Using gel-based TILLING we were able to identify different genes involved in non-complex traits like SCN resistance including a serine hydroxymethyltransferase (SHMT) and a Leucine Rich Repeat (LRR-RLK) (Liu et al., 2011, 2012). However, this method presented limitations in functional gene analysis in the case of complex traits (i.e., oil) due to the high copy number and multiple paralogs of the *FAD2* gene family in the soybean genome. Most likely, during the initial steps of the PCR, although the designed primers were specific to the targeted gene, the possibility of amplification of the other *FAD2* copies was present because of the high similarities shared between those genes. Therefore, these non-targeted genes were present within the amplicons more than with just their initial copy number. In this case, the PCR products anneal with the other *FAD2* copies, and the heteroduplex formed contain both the targeted amplicon from the PCR product and the non-specific amplicons. Forming the heteroduplex during TILLING resulted in non-specific hybridizations leading to the detection of false positive mutants by the mismatch enzyme.

### Identification of a new syntenic duplication segment surrounding fatty acid desaturases in the soybean genome

Homologous regions associated with a major seed protein content QTL in soybean have been reported to be present only on chr20, but not on chr10. The presence of a duplicated genomic regions harboring QTL for both protein/oil content has been reported between chr20 and chr10. It has been shown that this segment encompasses 27 conserved genes (Lestari et al., 2013).

In this study, new-duplicated segments between chromosomes chr10 and chr20, chr09 and chr03, chr09 and chr19, chr19 and chr03 surrounding and harboring the fatty acid desaturase gene family members (*FAD2*) have been identified and reported (Figure 2, Tables 3, 4 and Table S1). The duplicated segment between chr20 and chr10 reported in this study encompasses 8 new genes on a segment located apart from the region reported previously (≈5 Mb between each other) (Figure 2A, Table 2). Thus, this newly identified region is specific to genes surrounding the fatty acid desaturase-2 gene family within the soybean genome, and different from the duplication reported previously.

**Table 3:** **Gene divergence of duplicated regions between Chr03 (35.91–36.11 Mb) and Chr09 (22.07–22.27 Mb)**.

**Order within block**	**Locus 1**	**Locus 2**	**Putative function**	**Ka**	*Ks*	**Ka/Ks**
1	Glyma.03G143100	Glyma.09G113800	SBP domain	0.18	0.81	0.22
2	Glyma.03G143500	Glyma.09G113100	HOMEODOMAIN-LIKE SUPERFAMILY PROTEIN	0.53	1.7	0.31
3	Glyma.03G143600	Glyma.09G113000	MYB-LIKE HTH TRANSCRIPTIONAL REGULATOR FAMILY PROTEIN	0.21	0.5	0.42
4	Glyma.03G143800	Glyma.09G112700	Cotton fiber expressed protein	0.23	0.73	0.32
5	Glyma.03G144400	Glyma.09G112100	LATE EMBRYOGENESIS ABUNDANT PROTEIN 4-5	0.4	1.15	0.35
6	**Glyma.03G144500**	**Glyma.09G111900**	**OMEGA-6 FATTY ACID DESATURASE, ENDOPLASMIC RETICULUM**	**0.13**	**1.3**	**0.10**
7	Glyma.03G144700	Glyma.09G111500	UF	0.26	1	0.26
8	Glyma.03G144800	Glyma.09G111200	ACTIN	0.01	0.74	0.01
9	Glyma.03G144900	Glyma.09G111100	DnaJ domain	0.19	0.65	0.29
10	Glyma.03G145000	Glyma.09G111000	FAD diphosphatase/ FAD pyrophosphatase	0.09	0.58	0.16
11	Glyma.03G145500	Glyma.09G110100	WRC	0.36	0.89	0.40
12	Glyma.03G145600	Glyma.09G109800	PEROXIDASE 53-RELATED	0.11	0.85	0.13
13	Glyma.03G145800	Glyma.09G109700	Transcription factor HEX, contains HOX and HALZ domains Links B M	0.18	0.98	0.18
14	Glyma.03G145900	Glyma.09G109600	PROTEIN TRICHOME BIREFRINGENCE-RELATED	0.2	0.82	0.24
15	Glyma.03G146100	Glyma.09G109200	Uncharacterized conserved protein Links B M	0.19	0.77	0.25
16	Glyma.03G146400	Glyma.09G109100	Non-specific serine/threonine protein kinase	0.09	0.64	0.14
17	Glyma.03G146500	Glyma.09G109000	UF	0.15	0.42	0.36
18	Glyma.03G146900	Glyma.09G108000	KINESIN-RELATED PROTEIN 11	0.16	0.49	0.33
19	Glyma.03G147200	Glyma.09G107400	ZINC-FINGER TRANSCRIPTION FACTOR	0.38	1.41	0.27

*GmFAD2 in chr03 and chr09 belongs to a third duplicated segment containing 19 additional inverted duplicated genes or anchors. Analysis represent ±100 kb duplicated region centered in the GmFAD2 gene*.

**Table 4:** **Gene divergence of duplicated regions between Chr19 (40.71–40.91 Mb) and Chr09 (22.07–22.27 Mb)**.

**Order within block**	**Locus 1**	**Locus 2**	**Putative function**	**Ka**	**Ks**	**Ka/Ks**
1	Glyma.09G107400	Glyma.19G150800	ZINC-FINGER TRANSCRIPTION FACTOR	0.37	1.27	0.29
2	Glyma.09G108000	Glyma.19G150300	INESIN-RELATED PROTEIN 11	0.16	0.49	0.33
3	Glyma.09G109000	Glyma.19G149800	UP	0.15	0.42	0.36
4	Glyma.09G109100	Glyma.19G149700	Non-specific serine/threonine protein kinase	0.09	0.69	0.13
5	Glyma.09G109200	Glyma.19G149300	Uncharacterized conserved protein	0.19	0.71	0.27
6	Glyma.09G109600	Glyma.19G149100	PROTEIN TRICHOME BIREFRINGENCE-RELATED	0.21	0.95	0.22
7	Glyma.09G109700	Glyma.19G149000	HOMEOBOX-LEUCINE ZIPPER PROTEIN HAT14	0.24	1.06	0.23
8	Glyma.09G109800	Glyma.19G148800	PEROXIDASE 53-RELATED	0.11	0.77	0.14
9	Glyma.09G110100	Glyma.19G148700	WRC	0.35	0.76	0.46
10	Glyma.09G111000	Glyma.19G148100	FAD diphosphatase / FAD pyrophosphatase	0.09	0.66	0.14
11	Glyma.09G111100	Glyma.19G148000	DnaJ domain	0.21	0.57	0.37
12	Glyma.09G111200	Glyma.19G147900	ACTIN	0.01	0.67	0.01
13	Glyma.09G111300	Glyma.19G147800	PROTEIN PHOSPHATASE 2C 68-RELATED	0.13	0.74	0.18
14	Glyma.09G111500	Glyma.19G147700	UP	0.23	0.8	0.29
15	Glyma.09G111900	Glyma.19G147300	OMEGA-6 FATTY ACID DESATURASE, ER	0.15	1.23	0.12
16	Glyma.09G112100	Glyma.19G147200	LATE EMBRYOGENESIS ABUNDANT PROTEIN 4-5	0.43	1.07	0.40
17	Glyma.09G112600	Glyma.19G147000	UP	0.2	0.45	0.44
18	Glyma.09G112700	Glyma.19G146900	Cotton fiber expressed protein	0.23	0.78	0.29
19	Glyma.09G113000	Glyma.19G146600	MYB-LIKE HTH TRANSCRIPTIONAL REGULATOR FAMILY PROTEIN	0.21	0.52	0.40
20	Glyma.09G113100	Glyma.19G146500	MYB-LIKE HTH TRANSCRIPTIONAL REGULATOR FAMILY PROTEIN	0.54	1.11	0.49
21	Glyma.09G113800	Glyma.19G146000	SBP domain	0.19	0.71	0.27

*GmFAD2 in chr19 and chr09 belongs to a third duplicated segment containing 21 additional inverted duplicated genes or anchors. Analysis represent ±100 kb duplicated region centered in the GmFAD2 gene*.

### *GmFAD2* gene family evolved through soybean genome duplication

Various duplication events in crop species over time resulted in many polyploid genomes. Polyploidy has conferred diverse advantages to the development of important agronomic traits. Polyploidization has been associated with an increased size in harvested organs, novel gene interactions leading to new traits, and the formation of new crop species (Eckardt, 2004). In the soybean genome, it has been reported that two different large-scale duplication events occurred 13 and 59 million years ago, with subsequent divergent selection in many gene families favoring the diversification of this species (Schmutz et al., 2010; Yin et al., 2013; Zhu et al., 2014; Singh and Jain, 2015). Soybean has a paleopolyploid genome, and approximately 75% of predicted soybean genes are present in multiple copies (Schmutz et al., 2010). Fatty acid desaturases in the soybean genome are present under two *FAD2* copies: *FAD2-1* and *FAD2-2*. This study showed that at least seven members constitute the *FAD2* gene family and are present in six different linkage groups of the soybean genome; *FAD2-1A* (Glyma.10G278000), *FAD2-1B* (Glyma.20G111000), *FAD2-2A* (Glyma.19G147300), *FAD2-2B* (Glyma.19G147400), *FAD2-2C* (Glyma.03G144500), *FAD2-2D* (Glyma.09G111900), and *FAD2-2E* (Glyma.15G195200) (Table 1). In addition, *FAD2-1* members are closely related to one another, with a shared genomic organization containing a single intron and a 99% identity in the encoded amino acid sequence (Figure S6).

### Neofunctionalization and subfunctionalization within the *GmFAD2* gene family

The phylogenetic analysis within the *Glycine max FAD2* gene family indicates that GmFAD2-1A and GmFAD2-1B proteins form a separate clade from the five GmFAD2-2 members (Figure 2). Our mutational analysis demonstrated that only mutations within FAD2-1 members affect seed oleic acid content in soybean. In addition, data from RNAseq shows that the two microsomal *FAD2-1* desaturases *FAD2-1A* and *FAD2-1B* were mainly expressed in developing seeds and constituted the seed specific paralogs in the soybean genome (Figure S1). However, the expression profile of the five members constituting the *GmFAD2-2* gene family was different. While *GmFAD2-2B* and *GmFAD2-2C* were found to display ubiquitous expression in all the vegetative tissues of the soybean plant, *GmFAD2-2D* was expressed in the flower, seed, and nodule, while *GmFAD2-2E* expression was exclusively confined to the pod and seed with low levels of expression. The exception was *GmFAD2-2A*, in which no expression was detected. *GmFAD2-2A* has a deletion of 100 bp in the coding region and therefore was predicted to be non-functional (Pham et al., 2010).

Although GmFAD2-1 members are closely related to GmFAD2-2 members based on amino acid sequences, the reported differences in their expression and functional patterns in addition to the results obtained from syntenic and phylogenetic analysis, suggest that its subfunctionalization is driven by an accumulation of mutations and ongoing evolutionary selection pressures (Figure S6). The genome distribution of *GmFAD2* genes from syntenic analysis indicates the existence of segmental and tandem duplication events (Yin et al., 2013; Zhu et al., 2014). Therefore, we propose that successive duplications of an ancestral *FAD2* gene led to the subfunctionalization and/or neofunctionalization within this large gene family in soybean. Our results support the hypothesis that gene duplication is an important evolutionary mechanism in the generation of novel functions and phenotypes, contributing to the adaptation of land plants (Hanada et al., 2008; Dassanayake et al., 2011).

### Forward genetics screening as an alternative method to gel-based TILLING

Forward genetics screening was used to detect mutations in genes of interest. Gas chromatography was used for the identification of novel alleles with high oleic acid content and their associated mutations were identified by targeted gene sequencing. Phenomics is an important field of research that is consistently improving and can be used as an alternative strategy. Interestingly, using the forward genetic approach, high oleic acid soybean mutant lines were identified to carry missense mutations in either the *FAD2-1A* or *FAD2-1B* paralogs (Figures 3B, 4B). Oleic acid content in the seed oil of the mutants was up to 50.86% (Table S2). However, the levels of palmitic acid, stearic acid, and linolenic acid in seed oil were unchanged, with the exception of a small decrease in linoleic acid compared to the wild type Forrest (Tables 5, 6). Acknowledging the role of FAD2 in converting oleic acid to linoleic acid in the fatty acid biosynthetic pathway (Figure S5), the decrease in linoleic acid is possibly caused by the reduced activity of FAD2 members. Reduction of linoleic acid in these mutants is regarded as an agronomic improvement. It has been demonstrated that high linoleic acid content in soybean oil is potentially negative due to the associated reduction of healthy omega-3 fatty acids (Clark et al., 1992; Friesen and Innis, 2010; Blasbalg et al., 2011).

**Table 5:** **Levels of the five major fatty acids in the seed oil of the screened mutants and the wild type Forrest in the M3 (2012) and M4 (2013) generations**.

**Lines**	**Palmitic acid**		**Stearic acid**		**Oleic acid**		**Linoleic acid**		**Linolenic acid**		***n***	
	**2012 (M3)**	**2013 (M4)**	**2012 (M3)**	**2013 (M4)**	**2012 (M3)**	**2013 (M4)**	**2012 (M3)**	**2013 (M4)**	**2012 (M3)**	**2013 (M4)**	**2012 (M3)**	**2013 (M4)**
Forrest	11.59 ± 0.9	10.93 ± 0.59	3.32 ± 0.26	3.25 ± 0.15	**18.04** ± **3.92**	**18.89** ± **2.34**	54.52 ± 3.91	53.71 ± 3.28	6.19 ± 1.22	6.89 ± 0.79	21	9
F1274	9.20 ± 1.13	9.08 ± 1.14	3.10 ± 0.34	3.44 ± 1.14	**39.50** ± **8.40**	**29.73** ± **2.73**	31.50 ± 8.68	43.70 ± 2.55	5.30 ± 1.10	6.68 ± 1.40	5	4
F1284	8.20 ± 3.17	11.22 ± 0.67	2.61 ± 1.29	3.85 ± 0.55	**34.03** ± **7.19**	**42.81** ± **2.66**	38.18 ± 9.02	33.75 ± 3.80	6.34 ± 3.06	4.49 ± 1.15	4	4
F812	11.18 ± 1.57	9.64 ± 0.24	3.540.28	4.10 ± 0.77	**23.52** ± **2.60**	**27.82** ± **2.86**	47.91 ± 2.59	44.13 ± 3.19	6.06 ± 1.27	7.06 ± 0.45	4	4

*Fatty acid levels were averaged for n segregating M3 and M4 lines for each mutation. Averages and standard deviations are shown*.

**Table 6:** **Levels of the five major fatty acids in the seed oil of the re-screened mutants and the wild type Forrest in the M2 (2011) and M3 (2012) generations**.

**Lines**	**Palmitic acid**		**Stearic acid**		**Oleic acid**		**Linoleic acid**		**Linolenic acid**		***n***	
	**2011 (M2)**	**2012 (M3)**	**2011 (M2)**	**2012 (M3)**	**2011 (M2)**	**2012 (M3)**	**2011 (M2)**	**2012 (M3)**	**2011 (M2)**	**2012 (M3)**	**2011 (M2)**	**2012 (M3)**
Forrest	8.76 ± 0.09		2.05 ± 0.25		**14.25** ± **2.17**		47.04 ± 4.37		8.15 ± 1.65		3	
F784	8.64 ± 0.83	9.08 ± 1.96	3.42 ± 0.45	4.18 ± 0.44	**21.27** ± **2.91**	**29.00** ± **5.87**	40.63 ± 6.23	39.96 ± 4.69	4.43 ± 1.00	4.78 ± 1.49	2	3
F1235	8.98 ± 1.45	8.94 ± 1.46	3.25 ± 1.42	4.99 ± 0.09	**33.58** ± **15.50**	**25.89** ± **3.25**	42.80 ± 6.49	42.71 ± 0.07	6.32 ± 1.19	5.48 ± 0.15	2	3

*Fatty acid levels were averaged for n segregating M2 and M3 lines for each mutation Averages and standard deviations are shown*.

### TILLING by sequencing as alternative to gel-based TILLING

The availability of high-throughput phenotyping methods that allow for the identification of mutants with high oleic acid levels provide us with an alternative way to identify these mutants. However, a forward-screening approach also presents some limitations, including the cost and time involved in the phenotyping process. In addition, only a fraction of these mutants could be linked to mutations within well-known genes for fatty acid biosynthesis. Stability in the desired traits was shown in some mutants when advanced to the next generation. Half of the mutants identified were not stable once advanced to the next generation because of two possibilities. The first one is that the seeds that were taken to advance the population from the M2 family were segregating for the desired high oleic level. This presents 78% of the identified mutants, from which the original M2 seed stock was retested in order to identify mutations linked to the fatty acid biosynthesis pathway. The rest of the seeds that were found to show high oleic acid content were not linked to *FAD2* genes. This was most likely due to the genotype, epistatic environmental interactions, or other genes that may contribute to fatty acid biosynthesis indirectly. The mutants carrying mutations within the *FAD2-1* genes were identified by target sequencing; therefore, it's suggested to use TILLING by sequencing to replace the gel-based system in future works.

In this study, we showed the benefit of using ethyl-methanesulfonate (EMS) mutagenesis, which alkylates DNA and can cause significant changes to the genes and gene networks underlying oil biosynthesis. These changes are hard to accomplish under conventional breeding programs, which require diverse genetic material, and are usually more expensive and time-consuming than mutation breeding. It has also been reported that the highest oleic acid seed content due to natural variations in the soybean germplasm was around 48% (Pham et al., 2011). However, most of the tested lines were plant introductions (PIs) with poor agronomic performance. In this study, a cultivar was used to produce allele variants within *FAD2-1A* and *FAD2-1B*, with oleic acid levels up to 50%. The Forrest cultivar could be used to introgress the high oleic acid content trait into high-yielding lines without compromising their agronomic performance. EMS mutagenesis is also less time-consuming as two seasons were sufficient to develop an EMS-mutagenized population. This is a highly efficient technique for the characterization and identification of multiple beneficial agronomic traits in terms of time, cost, and productivity, not only in soybeans, but also in other plant crops.

## Author contributions

NL wrote the manuscript, made the corresponding figures, performed statistical analysis, primer design, sequencing analysis, syntenic and phylogenetic analysis, greenhouse and field management. SL performed TILLING analysis. NL, ZZ developed EMS mutagenized populations, performed fatty acid analysis, and PCR gel purification. NL, ZZ, SL performed DNA extractions. VC assisted in experiments, analysis, and drafting. AA provided GC analysis. KM planned the experiments, supervised the work and edited the manuscript. All authors reviewed and approved the final version.

## Funding

This research was supported in part by the United Soybean Board project #1520-532-5607 to Khalid Meksem.

### Conflict of interest statement

The authors declare that the research was conducted in the absence of any commercial or financial relationships that could be construed as a potential conflict of interest.

## Supplementary material

Figure S1 **Expression patterns of the six soybean ***FAD2*** gene members ***in planta***, based on Soyseq resource available from RNAsequencing data (**http://www.soybase.org/soyseq**)**. The figure shows that both *FAD2-1A* and *FAD2-1B* encode the seed specific paralogs of *FAD2-1*, while *FAD2-2B* and *FAD2-2C* paralogs are expressed in the vegetative tissues and the development of seeds. *FAD2-2A* expression was not detected. *FAD2-2E* expression was limited to the seed and pods, while *FAD2-2D* expression was detected in the flower, seed, and nodules.

Figure S2 **Schematic representation of the soybean ***FAD2-2B*** containing duplicated segments identified between the soybean and oil palm genomes**. *FAD2-2B* intergenome syntenic relationships were calculated using the Plant Genome Duplication Database. **(A)** *GmFAD2-B* in chr19 and *OlFAD2* in chr05 belongs to a duplicated segment containing 7 additional conserved duplicated genes or anchors. **(B)** *GmFAD2-B* in chr19 and OlFAD2 chr05 belong to another duplicated segment with 8 conserved genes. Graphs represent a ±100 kb duplicated region centered in the *GmFAD2-2B* gene.

Figure S3 **Representative results of the TILLING mutant screening using the mutant Forrest population**. Polyacrylamide gel represent TILLING screening of *FAD2-1A* and *FAD2-1B*, using 640 EMS mutagenized Forrest mutants from FM2-2013. **(A)** Image at Channel 700; **(B)** Image at Channel 800. Some false positive mutants from gel are shown. However, no mutations were identified after screening >2,000 individuals.

Figure S4 **The distribution of five major fatty acid contents in the seed oil of the Forrest M3 population**. The graphs represent the distribution of the palmitic, stearic, oleic, linoleic, and linolenic acid contents in seed oil of the Forrest M3 mutant population (*n* = 1,037). The dark color in the middle of each graph shows the wild type Forrest distribution (*n* = 21). Top of each graph represents a box plot representing the quartiles.

Figure S5 **The biosynthetic pathway of fatty acids**. Vertical direction represents the predominant biosynthetic pathway in soybean seeds. Gray bold arrows represent additional biosynthetic pathway in yeast.

Figure S6 **Protein sequence alignment of the seven members constituting the FAD2 gene family; FAD2-1A, FAD2-1B, FAD2-2A, FAD2-2B, FAD2-2C, FAD2-2D, and FAD2-2E**.

Figure S7 **The ***FAD2*** gene members in the soybean genome available in phytozome**.

Figure S8 **The ***FAD2-1*** splicing variants in the soybean genome available in phytozome**.

Figure S9 **The ***FAD2-2*** splicing variants in the soybean genome available in phytozome**.

Figure S10 *****FAD2*** mutations of the five screened oleic acid mutants**. Nucleotide sequence alignement of *FAD2-1A, FAD2-1B*, wild type Forrest, and the five screened mutants showing the identified mutations: F784 (FAD2-1A_C64G_), F1235 (FAD2-1A_C301T_), F1274 (FAD2-1A_C745T_), F1284 (FAD2-1A_C851T_), and F813 (FAD2-1B_C487T_). Only sequences containing nucleic acid changes are represented.

Table S1 **Gene divergence of duplicated regions between Chr19 (40.71–40.91 Mb) and Chr03 (35.91–36.11 Mb)**. GmFAD2 in chr19 and chr03 belongs to a very large duplicated segment containing 1,060 additional conserved duplicated genes or anchors. Analysis represent ±100 kb duplicated region centered in the GmFAD2 gene.

Table S2 **Levels of the five major fatty acids in the seed oil of the screened mutants**. The five fatty acid levels in seed oil of the four screened mutants and the wild type Forrest in the M3 (2012) and M4 (2013) generations. Fatty acid levels were averaged for n segregating the M3 and M4 lines for each mutation. Averages and standard deviations are shown.

Table S3 **Levels of the five major fatty acids the in seed oil of the re-screened high oleic mutants**. The five fatty acid levels in the seed oil of the eight re-screened mutants and the wild type Forrest in the M2 (2011) and M3 (2012) generations. Two replicates from the M2 and M3 lines are shown for each mutant. Asterisk (^*^) represents the oleic acid content from the first M3 screening. Underlined are mutants presenting significant seed oleic acid changes and maintaining high oleic levels from two generations.

Table S4 **Primers used for TILLING and sequencing**.

## Abbreviations

EMS: Ethyl methanesulfonate
FAD: fatty acid desaturase
SCN: soybean cyst nematode
SDS: sudden death syndrome
TILLING: targeted induced local lesions in genes
PI: plant introduction.
