# Supplementary figures and images for: Characterization of the FAD2 Gene Family in Soybean Reveals the Limitations of Gel-Based TILLING in Genes with High Copy Number
Source: Front Plant Sci. 2017 Mar 13;8:324. doi: 10.3389/fpls.2017.00324 (PMC5346563; doi:10.3389/fpls.2017.00324)

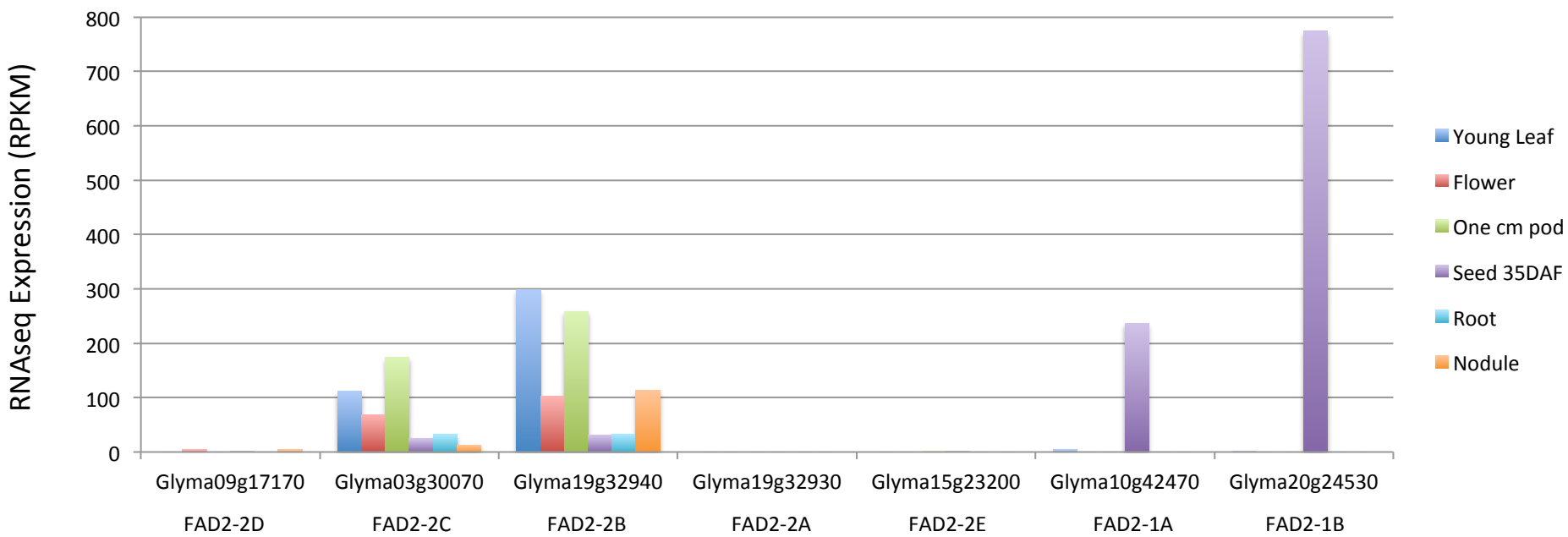

Supplement: Figure S1 — Expression patterns of the six soybean FAD2 gene members in planta, based on Soyseq resource available from RNAsequencing data (http://www.soybase.org/soyseq). The figure shows that both FAD2-1A and FAD2-1B encode the seed specific paralogs of FAD2-1, while FAD2-2B and FAD2-2C paralogs are expressed in the vegetative tissues and the development of seeds. FAD2-2A expression was not detected. FAD2-2E expression was limited to the seed and pods, while FAD2-2D expression was detected in the flower, seed, and nodules. [file DataSheet1.PDF]

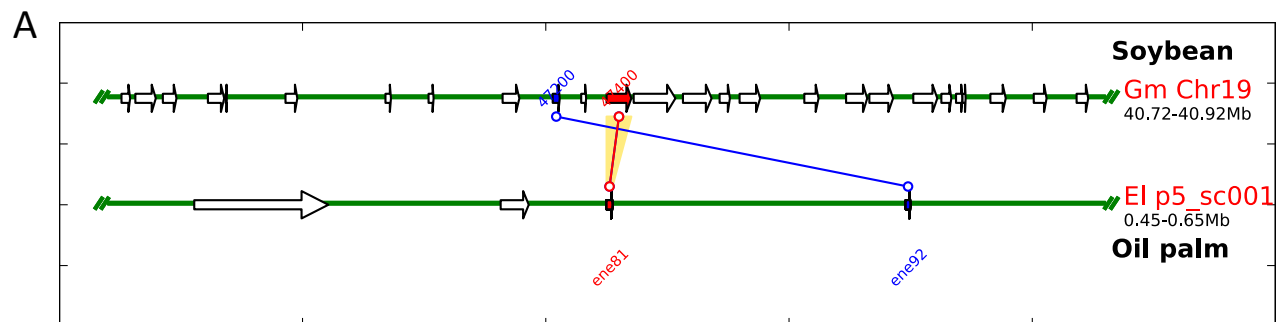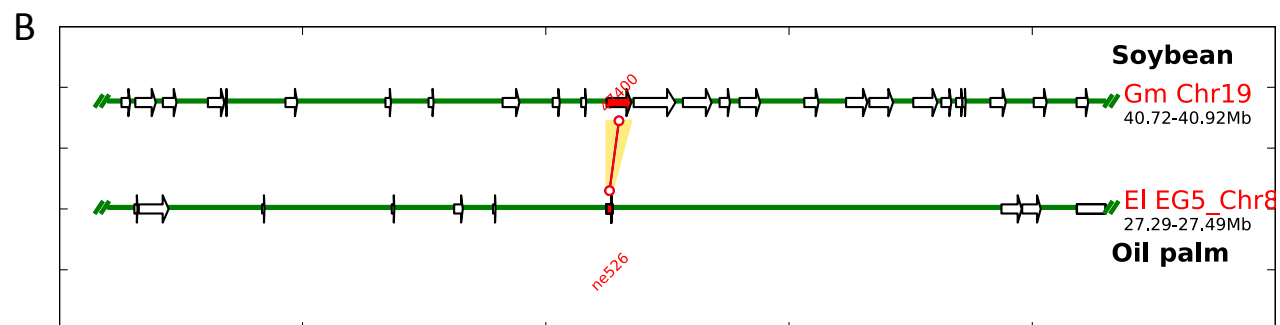

Supplement: Figure S2 — Schematic representation of the soybean FAD2-2B containing duplicated segments identified between the soybean and oil palm genomes. FAD2-2B intergenome syntenic relationships were calculated using the Plant Genome Duplication Database. (A) GmFAD2-B in chr19 and OlFAD2 in chr05 belongs to a duplicated segment containing 7 additional conserved duplicated genes or anchors. (B) GmFAD2-B in chr19 and OlFAD2 chr05 belong to another duplicated segment with 8 conserved genes. Graphs represent a ±100 kb duplicated region centered in the GmFAD2-2B gene. [file DataSheet2.PDF]

**A**

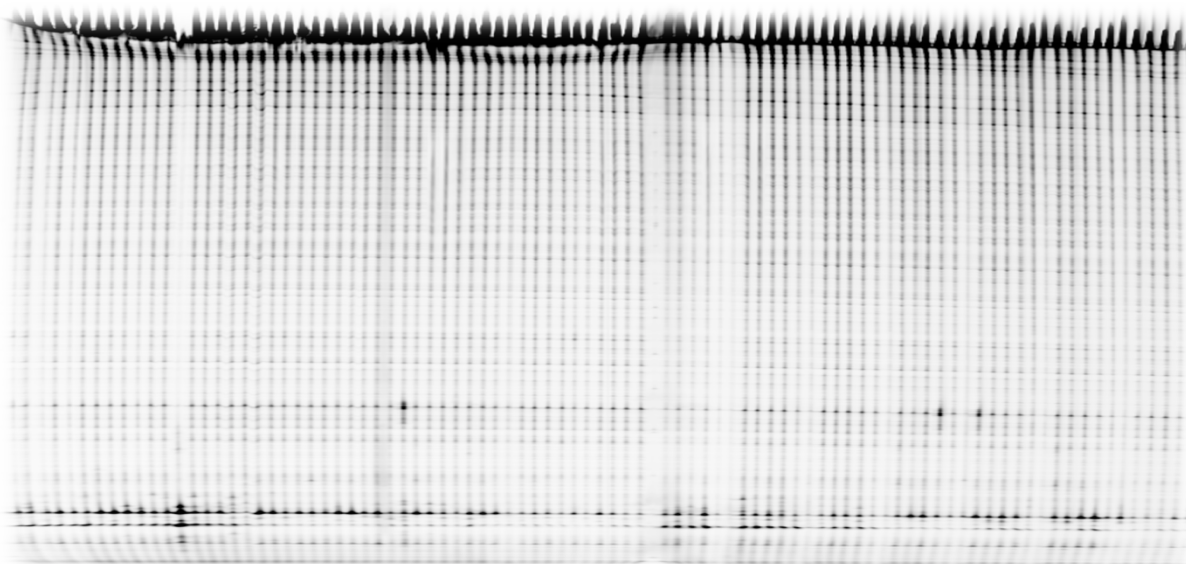

**B**

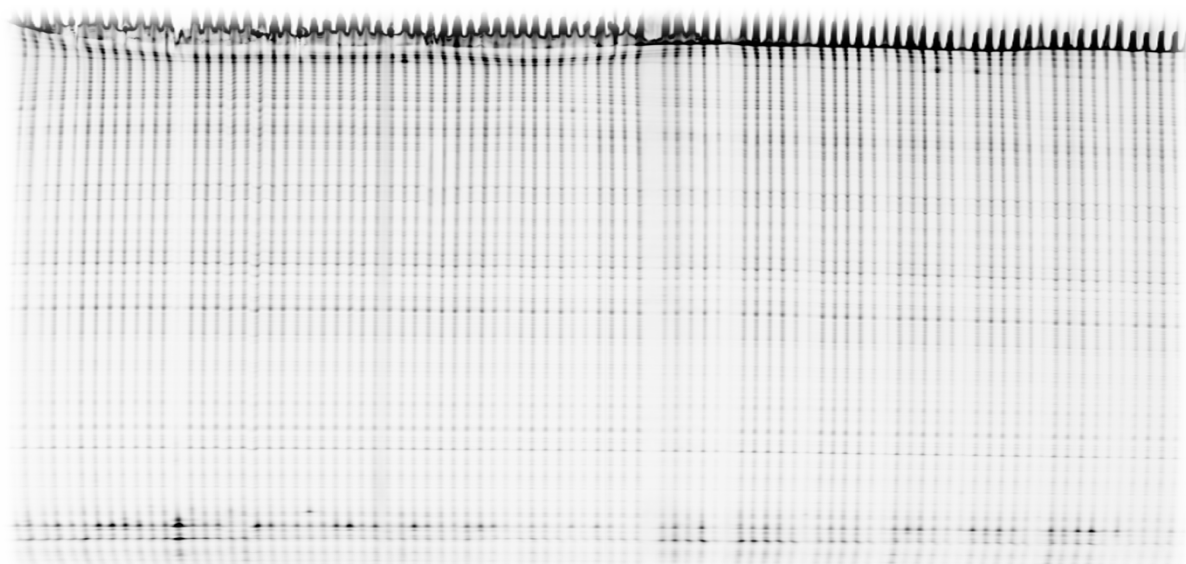

Supplement: Figure S3 — Representative results of the TILLING mutant screening using the mutant Forrest population. Polyacrylamide gel represent TILLING screening of FAD2-1A and FAD2-1B, using 640 EMS mutagenized Forrest mutants from FM2-2013. (A) Image at Channel 700; (B) Image at Channel 800. Some false positive mutants from gel are shown. However, no mutations were identified after screening >2,000 individuals. [file DataSheet3.PDF]

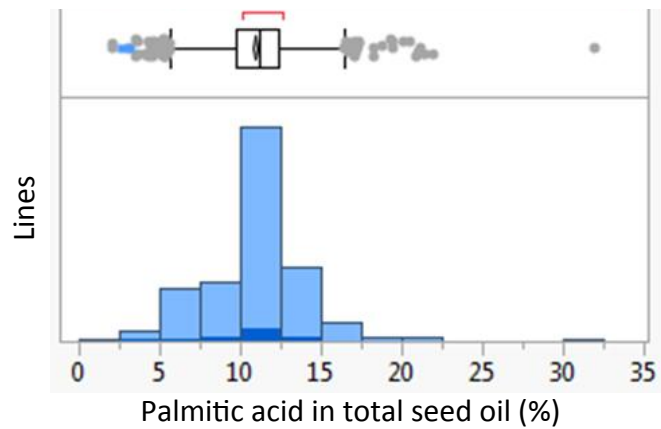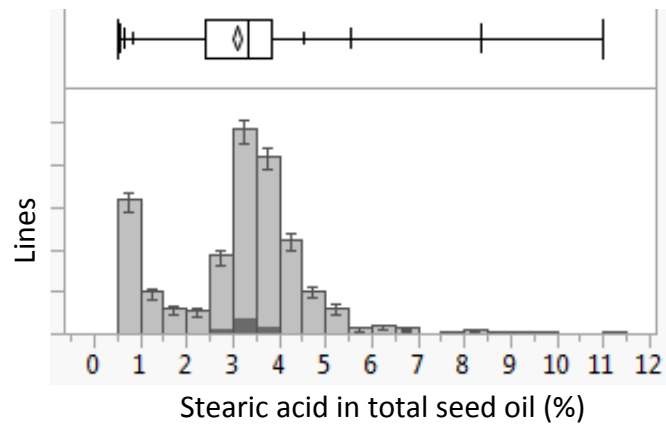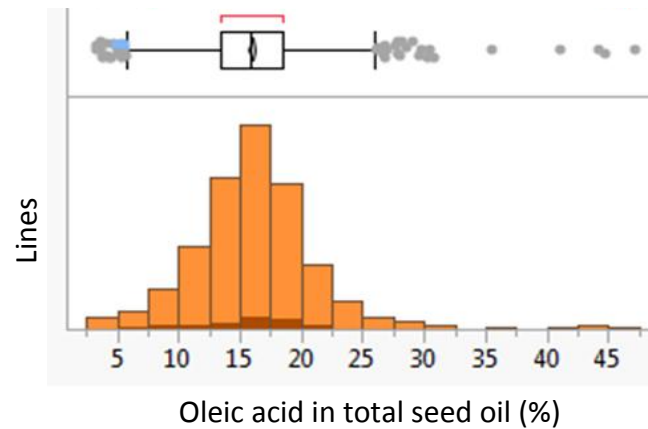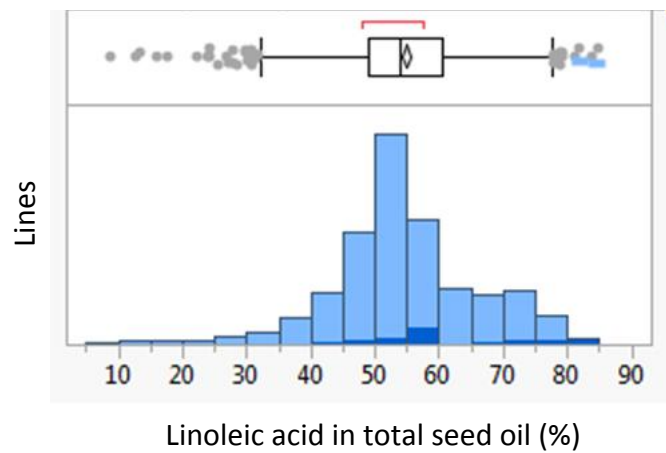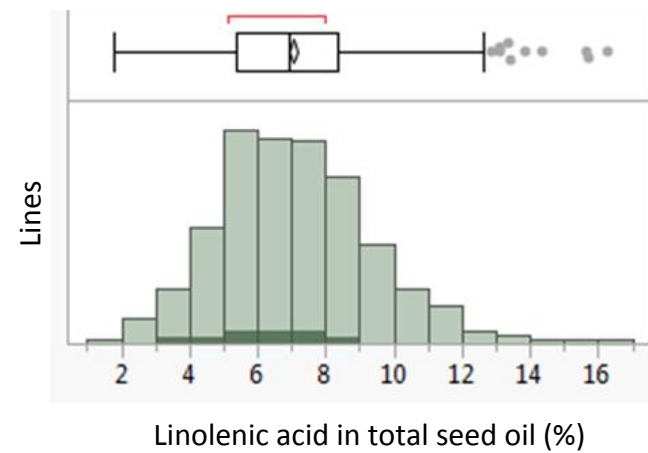

Supplement: Figure S4 — The distribution of five major fatty acid contents in the seed oil of the Forrest M3 population. The graphs represent the distribution of the palmitic, stearic, oleic, linoleic, and linolenic acid contents in seed oil of the Forrest M3 mutant population (n = 1,037). The dark color in the middle of each graph shows the wild type Forrest distribution (n = 21). Top of each graph represents a box plot representing the quartiles. [file DataSheet4.PDF]

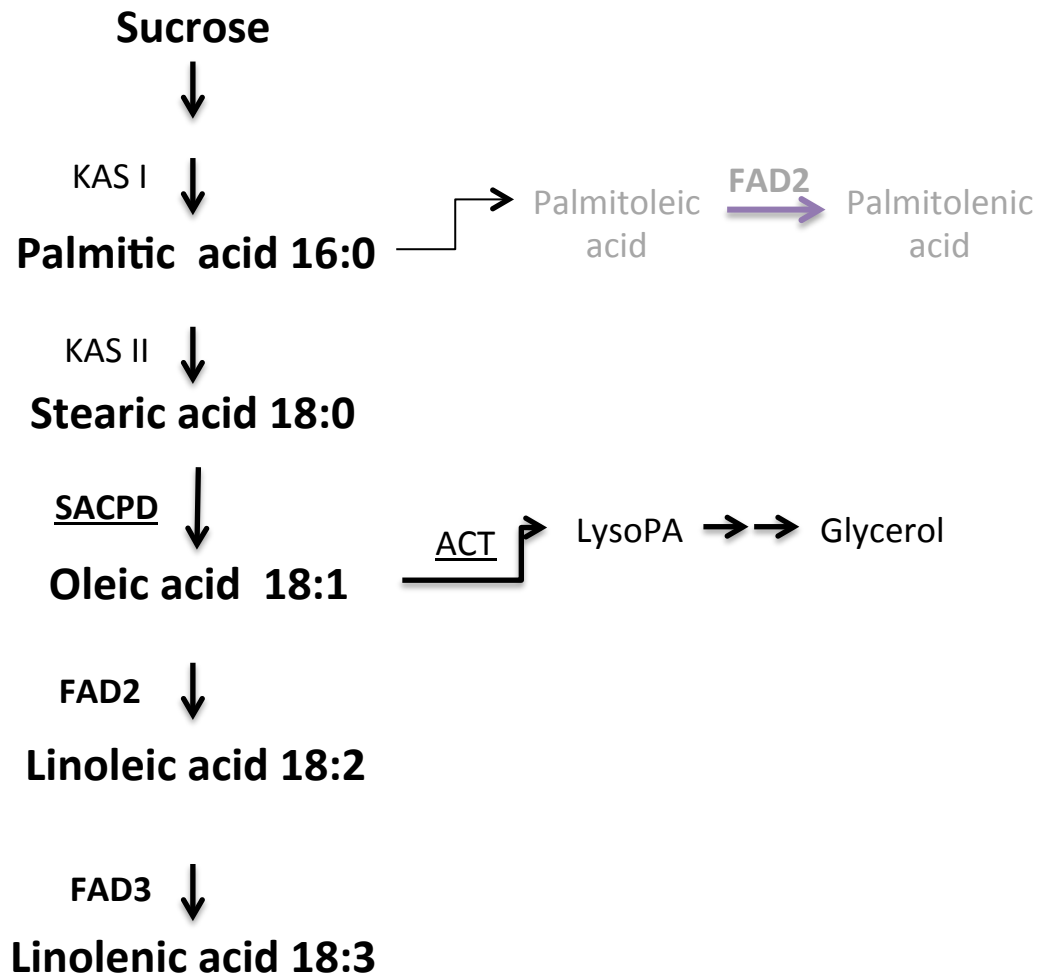

Supplement: Figure S5 — The biosynthetic pathway of fatty acids. Vertical direction represents the predominant biosynthetic pathway in soybean seeds. Gray bold arrows represent additional biosynthetic pathway in yeast. [file DataSheet5.PDF]
