# Supplementary material for: Characterization of the FAD2 Gene Family in Soybean Reveals the Limitations of Gel-Based TILLING in Genes with High Copy Number
Source: Front Plant Sci. 2017 Mar 13;8:324. doi: 10.3389/fpls.2017.00324 (PMC5346563; doi:10.3389/fpls.2017.00324)
Supplement: Figure S6 — Protein sequence alignment of the seven members constituting the FAD2 gene family; FAD2-1A, FAD2-1B, FAD2-2A, FAD2-2B, FAD2-2C, FAD2-2D, and FAD2-2E. [file DataSheet6.PDF]

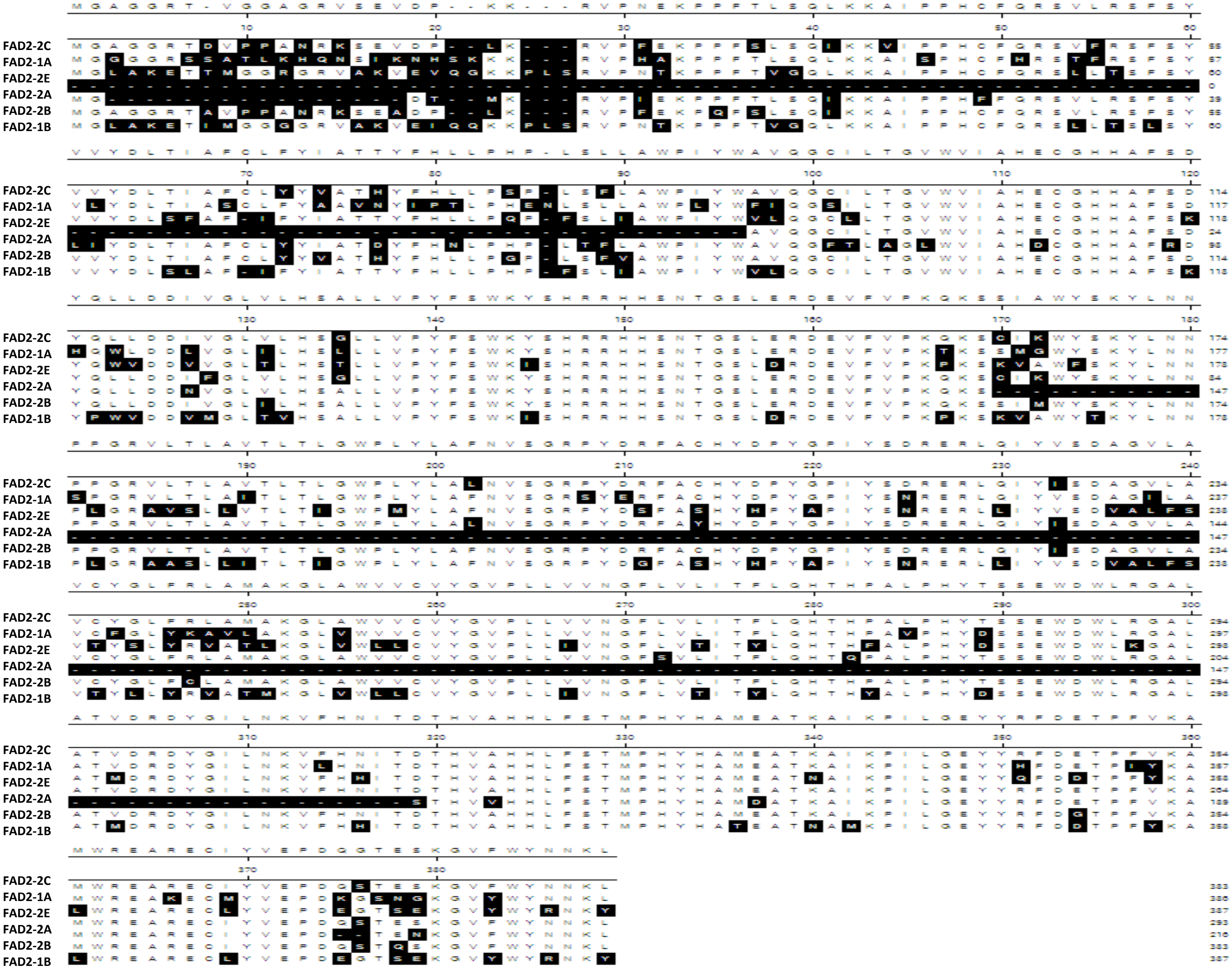
FAD2-2C
FAD2-1A
FAD2-2E
FAD2-2A
FAD2-2B
FAD2-1B
