# Supplementary material for: Characterization of the FAD2 Gene Family in Soybean Reveals the Limitations of Gel-Based TILLING in Genes with High Copy Number
Source: Front Plant Sci. 2017 Mar 13;8:324. doi: 10.3389/fpls.2017.00324 (PMC5346563; doi:10.3389/fpls.2017.00324)
Supplement: Figure S7 — The FAD2 gene members in the soybean genome available in phytozome. [file DataSheet7.PDF]

JGI Phytozome 11
THE PLANT GENOMICS RESOURCE

JGI HOME LOG IN

Species ▾ | Tools ▾ | Info ▾ | Download ▾ | Help ▾ | Cart | Subscribe

Previous view
Help with this page

Actions
- Revise query
- Launch Jalview
- Find related ...
- Add to cart
- Composite family

My Data (0)
- View cart
- Add to cart
- Upload user data
- Send to BioMart
- Send to PhytoMine
- Get from PhytoMine
- Quick download
- Delete data

Settings
- Species display
- Family filter
- Homolog filter

Search Results 5 genes found

Search criteria

Search term FAD2
Target Glycine max Wm82.a2.v1
Member filtering None
Ontologies matched 1

Genes | Ontologies

1-5 of 5 genes

	Views	Description
☐	G B	Glyma.19G147400.1.p - (M=2) 1.14.19.6 - Delta(12)-fatty-acid desaturase / Oleoyl-CoA Delta(12) desaturase
☐	G B	Glyma.03G144500.1.p - (M=2) 1.14.19.6 - Delta(12)-fatty-acid desaturase / Oleoyl-CoA Delta(12) desaturase
☐	G B	Glyma.09G111900.1.p - (M=3) PTHR32100:SF13 - OMEGA-6 FATTY ACID DESATURASE, ENDOPLASMIC RETICULUM
☐	G B	Glyma.20G111000.1.p - (M=12) PF11960 - Domain of unknown function (DUF3474) (DUF3474)
☐	G B	Glyma.10G278000.1.p - (M=28) PF00487 - Fatty acid desaturase (FA_desaturase)

Contact Disclaimer
Accessibility / Section 508 Statement
©1997-2015 The Regents of the University of California

U.S. DEPARTMENT OF ENERGY Office of Science
