# Supplementary material for: Characterization of the FAD2 Gene Family in Soybean Reveals the Limitations of Gel-Based TILLING in Genes with High Copy Number
Source: Front Plant Sci. 2017 Mar 13;8:324. doi: 10.3389/fpls.2017.00324 (PMC5346563; doi:10.3389/fpls.2017.00324)
Supplement: Figure S8 — The FAD2-1 splicing variants in the soybean genome available in phytozome. [file DataSheet8.PDF]

Previous view
Help with this page

Actions
Revise query
Launch Jalview
Find related ...
Add to cart
Composite family

My Data (0)
View cart
Add to cart
Upload user data
Send to BioMart
Send to PhytoMine
Get from PhytoMine
Quick download
Delete data

Settings
Species display
Family filter
Homolog filter

Gene Glyma.10G278000

Gene Info

Organism Glycine max
Locus Name Glyma.10G278000
Transcript Name Glyma.10G278000.1 (primary)
Other transcripts Glyma.10G278000.2
Location: Chr10:50013484..50015460 forward
Alias Glyma10g42470 Glyma10g42470.v1.1 Glyma10g42470.1.v1.1
Description (M=28) PF00487 - Fatty acid desaturase (FA_desaturase)
Links B M UniProt

Functional Annotation | Genomic | Sequences | Protein Homologs | Gene Ancestry | Variation | Expression

Show: All proteins | Primary proteins

Views	Org	Define	MRSF	Relationship	Score	Similarity	this gene (1–387)
G B	Gma	Glyma.10G278000.2			2551	100.0%	
G B	Gma	Glyma.20G111000.7			2429	97.7%	
G B	Gma	Glyma.20G111000.6			2429	97.7%	
G B	Gma	Glyma.20G111000.5			2429	97.7%	
G B	Gma	Glyma.20G111000.4			2429	97.7%	
G B	Gma	Glyma.20G111000.2			2429	97.7%	
G B	Gma	Glyma.20G111000.1 - (M=12) PF11960 - Domai...	FAB		2429	97.7%	
G B	Gma	Glyma.20G111000.3			2391	95.9%	
G B	Pvu	Phvul.007G023400.1 - (M=13) PF00487 - Fatty ...	FAB	M-1	1998	82.4%	
G B	Ccl	Ciclev10015614m - (M=2) 1.14.19.6 - Delta(12)-...	ROS		1973	85.0%	
G B	Csi	orange1.1g040843m - (M=2) 1.14.19.6 - Delta(1...	ROS		1971	85.0%	
G B	Tca	Thecc1EG040384t3 - Microsomal oleic acid de...			1962	82.7%	

©1997-2015 The Regents of the University of California
U.S. DEPARTMENT OF ENERGY Office of Science
