# Supplementary material for: Characterization of the FAD2 Gene Family in Soybean Reveals the Limitations of Gel-Based TILLING in Genes with High Copy Number
Source: Front Plant Sci. 2017 Mar 13;8:324. doi: 10.3389/fpls.2017.00324 (PMC5346563; doi:10.3389/fpls.2017.00324)
Supplement: Figure S9 — The FAD2-2 splicing variants in the soybean genome available in phytozome. [file DataSheet9.PDF]

JGI Phytozome 11
THE PLANT GENOMICS RESOURCE

Previous view
Help with this page

Actions
Revise query
Launch Jalview
Find related ...
Add to cart
Composite family

My Data (0)
View cart
Add to cart
Upload user data
Send to BioMart
Send to PhytoMine
Get from PhytoMine
Quick download
Delete data

Settings
Species display
Family filter
Homolog filter

Gene Glyma.19G147300

Gene Info

Organism Glycine max
Locus Name Glyma.19G147300
Transcript Name Glyma.19G147300.1 (primary)
Location: Chr19:40814864..40815855 forward
Alias Glyma19g32930 Glyma19g32930.v1.1
Description (M=3) PTHR32100:SF13 - OMEGA-6 FATTY ACID DESATURASE, ENDOPLASMIC RETICULUM
Links B M

Functional Annotation | Genomic | Sequences | Protein Homologs | Gene Ancestry | Variation | Expression

Show: All proteins | Primary proteins

	Views	Org	Define	MRSF	Relationship	Score	Similarity	1 this gene 216
	G B	Gma	Glyma.03G144500.2			1164	92.1%	
	G B	Gma	Glyma.03G144500.1 - (M=2) 1.14.19.6 - Delta(1…	FAB		1164	92.1%	
	G B	Pvu	Phvul.001G142100.1 - (M=2) 1.14.19.6 - Delta(1…	FAB		1163	92.1%	
	G B	Gma	Glyma.19G147400.2			1153	92.6%	
	G B	Gma	Glyma.19G147400.1 - (M=2) 1.14.19.6 - Delta(1…	FAB		1153	92.6%	
	G B	Mtr	Medtr7g093200.1 - microsomal omega-3 fatty …	FAB		1108	91.2%	
	G B	Csi	orange1.1g019605m			1072	89.4%	

©1997-2015 The Regents of the University of California

U.S. DEPARTMENT OF ENERGY Office of Science
