# Supplementary material for: Characterization of the FAD2 Gene Family in Soybean Reveals the Limitations of Gel-Based TILLING in Genes with High Copy Number
Source: Front Plant Sci. 2017 Mar 13;8:324. doi: 10.3389/fpls.2017.00324 (PMC5346563; doi:10.3389/fpls.2017.00324)
Supplement: Figure S10 — FAD2 mutations of the five screened oleic acid mutants. Nucleotide sequence alignement of FAD2-1A, FAD2-1B, wild type Forrest, and the five screened mutants showing the identified mutations: F784 (FAD2-1AC64G), F1235 (FAD2-1AC301T), F1274 (FAD2-1AC745T), F1284 (FAD2-1AC851T), and F813 (FAD2-1BC487T). Only sequences containing nucleic acid changes are represented. [file DataSheet10.PDF]

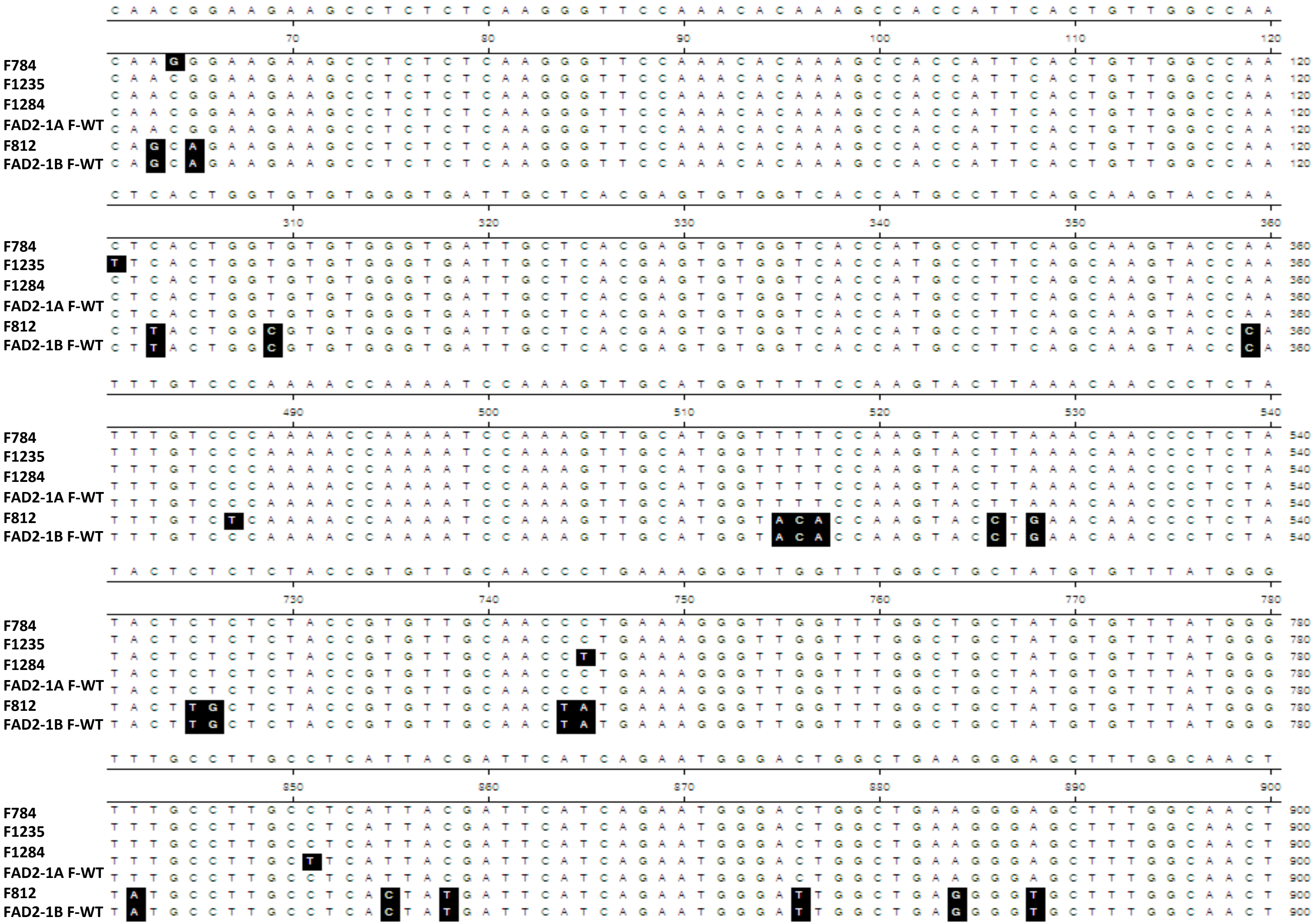
F784
F1235
F1284
FAD2-1A F-WT
F812
FAD2-1B F-WT
70
80
90
100
110
120
310
320
330
340
350
360
490
500
510
520
530
540
730
740
750
760
770
780
850
860
870
880
890
900
