# Supplementary material for: Characterization of the FAD2 Gene Family in Soybean Reveals the Limitations of Gel-Based TILLING in Genes with High Copy Number
Source: Front Plant Sci. 2017 Mar 13;8:324. doi: 10.3389/fpls.2017.00324 (PMC5346563; doi:10.3389/fpls.2017.00324)
Supplement: Table S1 — Gene divergence of duplicated regions between Chr19 (40.71–40.91 Mb) and Chr03 (35.91–36.11 Mb). GmFAD2 in chr19 and chr03 belongs to a very large duplicated segment containing 1,060 additional conserved duplicated genes or anchors. Analysis represent ±100 kb duplicated region centered in the GmFAD2 gene. [file Table1.PDF]

| Order within Block | Locus 1 | Locus 2 | Ka | Ks | Ka/Ks |
|---|---|---|---|---|---|
| 1 | Glyma.03G118800 | Glyma.19G122900 | 0.05 | 0.27 | 0.19 |
| 2 | Glyma.03G118900 | Glyma.19G123300 | 0.05 | 0.17 | 0.29 |
| 3 | Glyma.03G119000 | Glyma.19G123400 | 0.12 | 0.25 | 0.48 |
| 4 | Glyma.03G119200 | Glyma.19G123500 | 0.1 | 0.12 | 0.83 |
| 5 | Glyma.03G119300 | Glyma.19G123600 | 0.04 | 0.09 | 0.44 |
| 6 | Glyma.03G119400 | Glyma.19G123700 | 0.17 | 0.49 | 0.35 |
| 7 | Glyma.03G119500 | Glyma.19G123800 | 0.01 | 0.14 | 0.07 |
| 8 | Glyma.03G119700 | Glyma.19G123900 | 0.03 | 0.08 | 0.38 |
| 9 | Glyma.03G119900 | Glyma.19G124100 | 0.03 | 0.13 | 0.23 |
| 10 | Glyma.03G120000 | Glyma.19G124500 | 0.01 | 0.09 | 0.11 |
| 11 | Glyma.03G120200 | Glyma.19G124800 | 0.04 | 0.13 | 0.31 |
| 12 | Glyma.03G120300 | Glyma.19G124900 | 0.09 | 0.18 | 0.50 |
| 13 | Glyma.03G120400 | Glyma.19G125000 | 0.02 | 0.11 | 0.18 |
| 14 | Glyma.03G120500 | Glyma.19G125100 | 0.1 | 0.29 | 0.34 |
| 15 | Glyma.03G120600 | Glyma.19G125200 | 0.07 | 0.1 | 0.70 |
| 16 | Glyma.03G120700 | Glyma.19G125300 | 0.05 | 0.16 | 0.31 |
| 17 | Glyma.03G120800 | Glyma.19G125400 | 0.01 | 0.18 | 0.06 |
| 18 | Glyma.03G121000 | Glyma.19G125600 | 0.01 | 0.11 | 0.09 |
| 19 | Glyma.03G121100 | Glyma.19G125800 | 0.02 | 0.1 | 0.20 |
| 20 | Glyma.03G121200 | Glyma.19G125900 | 0.08 | 0.23 | 0.35 |
| 21 | Glyma.03G122100 | Glyma.19G126300 | 0.07 | 0.14 | 0.50 |
| 22 | Glyma.03G122200 | Glyma.19G126400 | 0.02 | 0.13 | 0.15 |
| 23 | Glyma.03G122400 | Glyma.19G126500 | 0.02 | 0.07 | 0.29 |
| 24 | Glyma.03G122500 | Glyma.19G126600 | 0.03 | 0.14 | 0.21 |
| 25 | Glyma.03G123100 | Glyma.19G126700 | 0.03 | 0.12 | 0.25 |
| 26 | Glyma.03G123200 | Glyma.19G126800 | 0.04 | 0.16 | 0.25 |
| 27 | Glyma.03G123300 | Glyma.19G126900 | 0.03 | 0.11 | 0.27 |
| 28 | Glyma.03G123400 | Glyma.19G127000 | 0.04 | 0.22 | 0.18 |
| 29 | Glyma.03G123500 | Glyma.19G127100 | 0.05 | 0.12 | 0.42 |
| 30 | Glyma.03G123600 | Glyma.19G127200 | 0.03 | 0.18 | 0.17 |
| 31 | Glyma.03G124100 | Glyma.19G127400 | 0.07 | 0.2 | 0.35 |
| 32 | Glyma.03G124400 | Glyma.19G127700 | 0 | 0.27 | 0.00 |
| 33 | Glyma.03G124700 | Glyma.19G127800 | 0.03 | 0.17 | 0.18 |
| 34 | Glyma.03G125000 | Glyma.19G127900 | 0.13 | 0.3 | 0.43 |
| 35 | Glyma.03G125200 | Glyma.19G128000 | 0.12 | 0.21 | 0.57 |
| 36 | Glyma.03G125800 | Glyma.19G128300 | 0.01 | 0.1 | 0.10 |
| 37 | Glyma.03G125900 | Glyma.19G128400 | 0.06 | 0.1 | 0.60 |
| 38 | Glyma.03G126000 | Glyma.19G128800 | 0.04 | 0.22 | 0.18 |
| 39 | Glyma.03G126100 | Glyma.19G128900 | 0.03 | 0.19 | 0.16 |
| 40 | Glyma.03G126200 | Glyma.19G129300 | 0.02 | 0.12 | 0.17 |
| 41 | Glyma.03G126300 | Glyma.19G129400 | 0.05 | 0.15 | 0.33 |
| 42 | Glyma.03G126400 | Glyma.19G129500 | 0.02 | 0.11 | 0.18 |
| 43 | Glyma.03G126600 | Glyma.19G129600 | 0.03 | 0.12 | 0.25 |

| | | | | | |
|---|---|---|---|---|---|
| 44 | Glyma.03G127000 | Glyma.19G129800 | 0.02 | 0.12 | 0.17 |
| 45 | Glyma.03G127100 | Glyma.19G129900 | 0.03 | 0.09 | 0.33 |
| 46 | Glyma.03G127300 | Glyma.19G130000 | 0.01 | 0.09 | 0.11 |
| 47 | Glyma.03G127500 | Glyma.19G130100 | 0.03 | 0.08 | 0.38 |
| 48 | Glyma.03G127600 | Glyma.19G130200 | 0.02 | 0.08 | 0.25 |
| 49 | Glyma.03G127800 | Glyma.19G130400 | 0.1 | 0.29 | 0.34 |
| 50 | Glyma.03G127900 | Glyma.19G130500 | 0.24 | 0.31 | 0.77 |
| 51 | Glyma.03G128000 | Glyma.19G130600 | 0.03 | 0.14 | 0.21 |
| 52 | Glyma.03G128100 | Glyma.19G130700 | 0.03 | 0.22 | 0.14 |
| 53 | Glyma.03G128300 | Glyma.19G130800 | 0.01 | 0.07 | 0.14 |
| 54 | Glyma.03G128400 | Glyma.19G130900 | 0.04 | 0.07 | 0.57 |
| 55 | Glyma.03G128600 | Glyma.19G131000 | 0.02 | 0.25 | 0.08 |
| 56 | Glyma.03G128700 | Glyma.19G131100 | 0.04 | 0.14 | 0.29 |
| 57 | Glyma.03G128800 | Glyma.19G131200 | 0.01 | 0.15 | 0.07 |
| 58 | Glyma.03G128900 | Glyma.19G131400 | 0.02 | 0.19 | 0.11 |
| 59 | Glyma.03G129100 | Glyma.19G131500 | 0.03 | 0.13 | 0.23 |
| 60 | Glyma.03G129400 | Glyma.19G131600 | 0.01 | 0.07 | 0.14 |
| 61 | Glyma.03G129500 | Glyma.19G131700 | 0.03 | 0.14 | 0.21 |
| 62 | Glyma.03G129600 | Glyma.19G131800 | 0.04 | 0.13 | 0.31 |
| 63 | Glyma.03G129700 | Glyma.19G132000 | 0.02 | 0.07 | 0.29 |
| 64 | Glyma.03G129800 | Glyma.19G132100 | 0.18 | 0.47 | 0.38 |
| 65 | Glyma.03G129900 | Glyma.19G132200 | 0.02 | 0.07 | 0.29 |
| 66 | Glyma.03G130000 | Glyma.19G132300 | 0.04 | 0.08 | 0.50 |
| 67 | Glyma.03G130100 | Glyma.19G132400 | 0.03 | 0.08 | 0.38 |
| 68 | Glyma.03G130400 | Glyma.19G132500 | 0.06 | 0.13 | 0.46 |
| 69 | Glyma.03G130700 | Glyma.19G132700 | 0.02 | 0.08 | 0.25 |
| 70 | Glyma.03G130800 | Glyma.19G132800 | 0.05 | 0.23 | 0.22 |
| 71 | Glyma.03G130900 | Glyma.19G132900 | 0.02 | 0.1 | 0.20 |
| 72 | Glyma.03G131000 | Glyma.19G133000 | 0.06 | 0.11 | 0.55 |
| 73 | Glyma.03G131100 | Glyma.19G133100 | 0.01 | 0.09 | 0.11 |
| 74 | Glyma.03G131200 | Glyma.19G133200 | 0.04 | 0.17 | 0.24 |
| 75 | Glyma.03G131400 | Glyma.19G133300 | 0.06 | 0.16 | 0.38 |
| 76 | Glyma.03G131600 | Glyma.19G133500 | 0.06 | 0.17 | 0.35 |
| 77 | Glyma.03G131700 | Glyma.19G133600 | 0.03 | 0.1 | 0.30 |
| 78 | Glyma.03G131800 | Glyma.19G133700 | 0.08 | 0.09 | 0.89 |
| 79 | Glyma.03G131900 | Glyma.19G133800 | 0.01 | 0.2 | 0.05 |
| 80 | Glyma.03G132000 | Glyma.19G133900 | 0.05 | 0.22 | 0.23 |
| 81 | Glyma.03G132200 | Glyma.19G134000 | 0.03 | 0.1 | 0.30 |
| 82 | Glyma.03G132300 | Glyma.19G134500 | 0.02 | 0.16 | 0.13 |
| 83 | Glyma.03G132600 | Glyma.19G134700 | 0.13 | 0.5 | 0.26 |
| 84 | Glyma.03G133000 | Glyma.19G134900 | 0.18 | 0.19 | 0.95 |
| 85 | Glyma.03G133200 | Glyma.19G135000 | 0.02 | 0.14 | 0.14 |
| 86 | Glyma.03G133300 | Glyma.19G135100 | 0.03 | 0.11 | 0.27 |
| 87 | Glyma.03G133400 | Glyma.19G135200 | 0.03 | 0.15 | 0.20 |
| 88 | Glyma.03G133500 | Glyma.19G135300 | 0.07 | 0.13 | 0.54 |

| | | | | | |
|---|---|---|---|---|---|
| 89 | Glyma.03G133600 | Glyma.19G135900 | 0.13 | 0.12 | 1.08 |
| 90 | Glyma.03G133700 | Glyma.19G136000 | 0.02 | 0.08 | 0.25 |
| 91 | Glyma.03G133800 | Glyma.19G136100 | 0.01 | 0.15 | 0.07 |
| 92 | Glyma.03G133900 | Glyma.19G136200 | 0.05 | 0.11 | 0.45 |
| 93 | Glyma.03G134100 | Glyma.19G136300 | 0.01 | 0.19 | 0.05 |
| 94 | Glyma.03G134200 | Glyma.19G136400 | 0.02 | 0.09 | 0.22 |
| 95 | Glyma.03G134300 | Glyma.19G136500 | 0.04 | 0.11 | 0.36 |
| 96 | Glyma.03G134400 | Glyma.19G136600 | 0.06 | 0.12 | 0.50 |
| 97 | Glyma.03G134500 | Glyma.19G136700 | 0.03 | 0.07 | 0.43 |
| 98 | Glyma.03G134600 | Glyma.19G136800 | 0.01 | 0.16 | 0.06 |
| 99 | Glyma.03G134700 | Glyma.19G137300 | 0.06 | 0.22 | 0.27 |
| 100 | Glyma.03G134800 | Glyma.19G137400 | 0.02 | 0.16 | 0.13 |
| 101 | Glyma.03G134900 | Glyma.19G137500 | 0.04 | 0.09 | 0.44 |
| 102 | Glyma.03G135000 | Glyma.19G137600 | 0.02 | 0.1 | 0.20 |
| 103 | Glyma.03G135200 | Glyma.19G137700 | 0.12 | 0.29 | 0.41 |
| 104 | Glyma.03G135800 | Glyma.19G137800 | 0.04 | 0.15 | 0.27 |
| 105 | Glyma.03G135900 | Glyma.19G137900 | 0.5 | 0.77 | 0.65 |
| 106 | Glyma.03G136100 | Glyma.19G138000 | 0.02 | 0.12 | 0.17 |
| 107 | Glyma.03G136200 | Glyma.19G138100 | 0.13 | 0.22 | 0.59 |
| 108 | Glyma.03G136300 | Glyma.19G138200 | 0.06 | 0.25 | 0.24 |
| 109 | Glyma.03G136500 | Glyma.19G138700 | 0.02 | 0.1 | 0.20 |
| 110 | Glyma.03G136600 | Glyma.19G139000 | 0.13 | 0.39 | 0.33 |
| 111 | Glyma.03G136800 | Glyma.19G139100 | 0.04 | 0.14 | 0.29 |
| 112 | Glyma.03G136900 | Glyma.19G139200 | 0.06 | 0.1 | 0.60 |
| 113 | Glyma.03G137000 | Glyma.19G139300 | 0.01 | 0.12 | 0.08 |
| 114 | Glyma.03G137100 | Glyma.19G139800 | 0.02 | 0.08 | 0.25 |
| 115 | Glyma.03G137300 | Glyma.19G140100 | 0.01 | 0.11 | 0.09 |
| 116 | Glyma.03G137400 | Glyma.19G140200 | 0.16 | 0.3 | 0.53 |
| 117 | Glyma.03G137500 | Glyma.19G140400 | 0.04 | 0.16 | 0.25 |
| 118 | Glyma.03G137600 | Glyma.19G140500 | 0.02 | 0.1 | 0.20 |
| 119 | Glyma.03G137700 | Glyma.19G140600 | 0.03 | 0.09 | 0.33 |
| 120 | Glyma.03G137900 | Glyma.19G140700 | 0.13 | 0.3 | 0.43 |
| 121 | Glyma.03G138000 | Glyma.19G140800 | 0.01 | 0.16 | 0.06 |
| 122 | Glyma.03G138100 | Glyma.19G141100 | 0.11 | 0.2 | 0.55 |
| 123 | Glyma.03G138200 | Glyma.19G141200 | 0.02 | 0.12 | 0.17 |
| 124 | Glyma.03G138300 | Glyma.19G141300 | 0.07 | 0.13 | 0.54 |
| 125 | Glyma.03G138400 | Glyma.19G141400 | 0.11 | 0.23 | 0.48 |
| 126 | Glyma.03G138600 | Glyma.19G141700 | 0.06 | 0.2 | 0.30 |
| 127 | Glyma.03G138700 | Glyma.19G141900 | 0.3 | 0.66 | 0.45 |
| 128 | Glyma.03G138900 | Glyma.19G142000 | 0.04 | 0.15 | 0.27 |
| 129 | Glyma.03G139000 | Glyma.19G142100 | 0.01 | 0.1 | 0.10 |
| 130 | Glyma.03G139100 | Glyma.19G142200 | 0.11 | 0.18 | 0.61 |
| 131 | Glyma.03G139200 | Glyma.19G142400 | 0.01 | 0.14 | 0.07 |
| 132 | Glyma.03G139700 | Glyma.19G142500 | 0.02 | 0.11 | 0.18 |
| 133 | Glyma.03G139800 | Glyma.19G142600 | 0.03 | 0.13 | 0.23 |

| | | | | | |
|---|---|---|---|---|---|
| 134 | Glyma.03G139900 | Glyma.19G142700 | 0.04 | 0.24 | 0.17 |
| 135 | Glyma.03G140000 | Glyma.19G142900 | 0.06 | 0.21 | 0.29 |
| 136 | Glyma.03G140100 | Glyma.19G143000 | 0.03 | 0.12 | 0.25 |
| 137 | Glyma.03G140500 | Glyma.19G143200 | 0.03 | 0.1 | 0.30 |
| 138 | Glyma.03G140700 | Glyma.19G143300 | 0.02 | 0.12 | 0.17 |
| 139 | Glyma.03G140900 | Glyma.19G143600 | 0.03 | 0.12 | 0.25 |
| 140 | Glyma.03G141000 | Glyma.19G143700 | 0.92 | 0 | #DIV/0! |
| 141 | Glyma.03G141200 | Glyma.19G143900 | 0.04 | 0.13 | 0.31 |
| 142 | Glyma.03G141500 | Glyma.19G144000 | 0.06 | 0.15 | 0.40 |
| 143 | Glyma.03G141600 | Glyma.19G144100 | 0.05 | 0.28 | 0.18 |
| 144 | Glyma.03G141700 | Glyma.19G144200 | 0.04 | 0.1 | 0.40 |
| 145 | Glyma.03G141800 | Glyma.19G144400 | 0.08 | 0.19 | 0.42 |
| 146 | Glyma.03G142000 | Glyma.19G144600 | 0.52 | 1.91 | 0.27 |
| 147 | Glyma.03G142200 | Glyma.19G145000 | 0.01 | 0.27 | 0.04 |
| 148 | Glyma.03G142400 | Glyma.19G145300 | 0.02 | 0.12 | 0.17 |
| 149 | Glyma.03G142500 | Glyma.19G145400 | 0.01 | 0.18 | 0.06 |
| 150 | Glyma.03G142600 | Glyma.19G145500 | 0.03 | 0.12 | 0.25 |
| 151 | Glyma.03G142800 | Glyma.19G145800 | 0.02 | 0.09 | 0.22 |
| 152 | Glyma.03G142900 | Glyma.19G145900 | 0.01 | 0.14 | 0.07 |
| 153 | Glyma.03G143100 | Glyma.19G146000 | 0.04 | 0.13 | 0.31 |
| 154 | Glyma.03G143200 | Glyma.19G146200 | 0.02 | 0.13 | 0.15 |
| 155 | Glyma.03G143400 | Glyma.19G146400 | 0.04 | 0.11 | 0.36 |
| 156 | Glyma.03G143500 | Glyma.19G146500 | 0.05 | 0.15 | 0.33 |
| 157 | Glyma.03G143600 | Glyma.19G146600 | 0.05 | 0.15 | 0.33 |
| 158 | Glyma.03G143700 | Glyma.19G146800 | 0.02 | 0.15 | 0.13 |
| 159 | Glyma.03G143800 | Glyma.19G146900 | 0.07 | 0.13 | 0.54 |
| 160 | Glyma.03G143900 | Glyma.19G147000 | 0.02 | 0.07 | 0.29 |
| 161 | Glyma.03G144200 | Glyma.19G147100 | 0.02 | 0.11 | 0.18 |
| 162 | Glyma.03G144400 | Glyma.19G147200 | 0.02 | 0.32 | 0.06 |
| 163 | Glyma.03G144500 | Glyma.19G147300 | 0.06 | 0.33 | 0.18 |
| 164 | Glyma.03G144600 | Glyma.19G147500 | 0.04 | 0.11 | 0.36 |
| 165 | Glyma.03G144700 | Glyma.19G147700 | 0.04 | 0.12 | 0.33 |
| 166 | Glyma.03G144800 | Glyma.19G147900 | 0 | 0.15 | 0.00 |
| 167 | Glyma.03G144900 | Glyma.19G148000 | 0.12 | 0.22 | 0.55 |
| 168 | Glyma.03G145000 | Glyma.19G148100 | 0.02 | 0.08 | 0.25 |
| 169 | Glyma.03G145200 | Glyma.19G148200 | 0.02 | 0.06 | 0.33 |
| 170 | Glyma.03G145300 | Glyma.19G148300 | 0.04 | 0.14 | 0.29 |
| 171 | Glyma.03G145400 | Glyma.19G148600 | 0.06 | 0.18 | 0.33 |
| 172 | Glyma.03G145500 | Glyma.19G148700 | 0.04 | 0.11 | 0.36 |
| 173 | Glyma.03G145600 | Glyma.19G148800 | 0.02 | 0.16 | 0.13 |
| 174 | Glyma.03G145700 | Glyma.19G148900 | 0.02 | 0.11 | 0.18 |
| 175 | Glyma.03G145800 | Glyma.19G149000 | 0.07 | 0.16 | 0.44 |
| 176 | Glyma.03G145900 | Glyma.19G149100 | 0.04 | 0.17 | 0.24 |
| 177 | Glyma.03G146000 | Glyma.19G149200 | 0.03 | 0.15 | 0.20 |
| 178 | Glyma.03G146100 | Glyma.19G149300 | 0.04 | 0.27 | 0.15 |

| | | | | | |
|---|---|---|---|---|---|
| 179 | Glyma.03G146200 | Glyma.19G149500 | 0.03 | 0.15 | 0.20 |
| 180 | Glyma.03G146300 | Glyma.19G149600 | 0.02 | 0.13 | 0.15 |
| 181 | Glyma.03G146400 | Glyma.19G149700 | 0.01 | 0.15 | 0.07 |
| 182 | Glyma.03G146500 | Glyma.19G149800 | 0.03 | 0.08 | 0.38 |
| 183 | Glyma.03G146600 | Glyma.19G149900 | 0.04 | 0.17 | 0.24 |
| 184 | Glyma.03G146700 | Glyma.19G150000 | 0.09 | 0.17 | 0.53 |
| 185 | Glyma.03G146900 | Glyma.19G150300 | 0.02 | 0.09 | 0.22 |
| 186 | Glyma.03G147000 | Glyma.19G150400 | 0.02 | 0.09 | 0.22 |
| 187 | Glyma.03G147100 | Glyma.19G150500 | 0.03 | 0.11 | 0.27 |
| 188 | Glyma.03G147200 | Glyma.19G150800 | 0.05 | 0.14 | 0.36 |
| 189 | Glyma.03G147600 | Glyma.19G150900 | 0.18 | 0.5 | 0.36 |
| 190 | Glyma.03G148100 | Glyma.19G151400 | 0.02 | 0.27 | 0.07 |
| 191 | Glyma.03G148200 | Glyma.19G151500 | 0.06 | 0.08 | 0.75 |
| 192 | Glyma.03G148400 | Glyma.19G151700 | 0.05 | 0.08 | 0.63 |
| 193 | Glyma.03G148500 | Glyma.19G151800 | 0.03 | 0.23 | 0.13 |
| 194 | Glyma.03G148600 | Glyma.19G151900 | 0.06 | 0.1 | 0.60 |
| 195 | Glyma.03G148700 | Glyma.19G152000 | 0.05 | 0.13 | 0.38 |
| 196 | Glyma.03G148800 | Glyma.19G152100 | 0.03 | 0.11 | 0.27 |
| 197 | Glyma.03G149000 | Glyma.19G152200 | 0.05 | 0.25 | 0.20 |
| 198 | Glyma.03G149200 | Glyma.19G152400 | 0.04 | 0.16 | 0.25 |
| 199 | Glyma.03G149500 | Glyma.19G152500 | 0.07 | 0.16 | 0.44 |
| 200 | Glyma.03G149600 | Glyma.19G152600 | 0.02 | 0.08 | 0.25 |
| 201 | Glyma.03G149700 | Glyma.19G152700 | 0.09 | 0.12 | 0.75 |
| 202 | Glyma.03G149900 | Glyma.19G152900 | 0.04 | 0.14 | 0.29 |
| 203 | Glyma.03G150100 | Glyma.19G153000 | 0.05 | 0.07 | 0.71 |
| 204 | Glyma.03G150400 | Glyma.19G153100 | 0.06 | 0.08 | 0.75 |
| 205 | Glyma.03G150600 | Glyma.19G153200 | 0.01 | 0.15 | 0.07 |
| 206 | Glyma.03G150700 | Glyma.19G153300 | 0.04 | 0.11 | 0.36 |
| 207 | Glyma.03G150800 | Glyma.19G153400 | 0.02 | 0.1 | 0.20 |
| 208 | Glyma.03G150900 | Glyma.19G153500 | 0 | 0.08 | 0.00 |
| 209 | Glyma.03G151100 | Glyma.19G153600 | 0.09 | 0.2 | 0.45 |
| 210 | Glyma.03G151200 | Glyma.19G153700 | 0.05 | 0.12 | 0.42 |
| 211 | Glyma.03G151300 | Glyma.19G153800 | 0.03 | 0.09 | 0.33 |
| 212 | Glyma.03G151400 | Glyma.19G153900 | 0.04 | 0.09 | 0.44 |
| 213 | Glyma.03G151500 | Glyma.19G154100 | 0.09 | 0.19 | 0.47 |
| 214 | Glyma.03G151700 | Glyma.19G154300 | 0.06 | 0.21 | 0.29 |
| 215 | Glyma.03G151800 | Glyma.19G154400 | 0.07 | 0.31 | 0.23 |
| 216 | Glyma.03G152000 | Glyma.19G154500 | 0.02 | 0.07 | 0.29 |
| 217 | Glyma.03G152100 | Glyma.19G154600 | 0 | 0.12 | 0.00 |
| 218 | Glyma.03G152200 | Glyma.19G154700 | 0.07 | 0.09 | 0.78 |
| 219 | Glyma.03G152300 | Glyma.19G154800 | 0.02 | 0.09 | 0.22 |
| 220 | Glyma.03G152400 | Glyma.19G154900 | 0.03 | 0.08 | 0.38 |
| 221 | Glyma.03G152600 | Glyma.19G155100 | 0.06 | 0.11 | 0.55 |
| 222 | Glyma.03G152700 | Glyma.19G155200 | 0.02 | 0.13 | 0.15 |
| 223 | Glyma.03G152800 | Glyma.19G155300 | 0.09 | 0.26 | 0.35 |

| | | | | | |
|---|---|---|---|---|---|
| 224 | Glyma.03G153000 | Glyma.19G155400 | 0.21 | 0.45 | 0.47 |
| 225 | Glyma.03G153100 | Glyma.19G155500 | 0.04 | 0.18 | 0.22 |
| 226 | Glyma.03G153200 | Glyma.19G155600 | 0.06 | 0.1 | 0.60 |
| 227 | Glyma.03G153300 | Glyma.19G155700 | 0.02 | 0.08 | 0.25 |
| 228 | Glyma.03G153400 | Glyma.19G155800 | 0.09 | 0.17 | 0.53 |
| 229 | Glyma.03G153500 | Glyma.19G155900 | 0.07 | 0.05 | 1.40 |
| 230 | Glyma.03G153600 | Glyma.19G156000 | 0.06 | 0.2 | 0.30 |
| 231 | Glyma.03G153800 | Glyma.19G156200 | 0.02 | 0.12 | 0.17 |
| 232 | Glyma.03G153900 | Glyma.19G156300 | 0.03 | 0.13 | 0.23 |
| 233 | Glyma.03G154000 | Glyma.19G156400 | 0.07 | 0.15 | 0.47 |
| 234 | Glyma.03G154200 | Glyma.19G156700 | 0.05 | 0.2 | 0.25 |
| 235 | Glyma.03G154400 | Glyma.19G156800 | 0.11 | 0.21 | 0.52 |
| 236 | Glyma.03G154600 | Glyma.19G156900 | 0.04 | 0.11 | 0.36 |
| 237 | Glyma.03G154800 | Glyma.19G157100 | 0.01 | 0.04 | 0.25 |
| 238 | Glyma.03G155000 | Glyma.19G157200 | 0.01 | 0.19 | 0.05 |
| 239 | Glyma.03G155100 | Glyma.19G157300 | 0.02 | 0.11 | 0.18 |
| 240 | Glyma.03G155300 | Glyma.19G157500 | 0.04 | 0.16 | 0.25 |
| 241 | Glyma.03G155400 | Glyma.19G157600 | 0.01 | 0.09 | 0.11 |
| 242 | Glyma.03G155600 | Glyma.19G157700 | 0.09 | 0.14 | 0.64 |
| 243 | Glyma.03G155700 | Glyma.19G157800 | 0.05 | 0.2 | 0.25 |
| 244 | Glyma.03G155800 | Glyma.19G158100 | 0.03 | 0.11 | 0.27 |
| 245 | Glyma.03G155900 | Glyma.19G158200 | 0.03 | 0.13 | 0.23 |
| 246 | Glyma.03G156000 | Glyma.19G158300 | 0.02 | 0.11 | 0.18 |
| 247 | Glyma.03G156100 | Glyma.19G158400 | 0.04 | 0.14 | 0.29 |
| 248 | Glyma.03G156200 | Glyma.19G158500 | 0.03 | 0.09 | 0.33 |
| 249 | Glyma.03G156300 | Glyma.19G158600 | 0.13 | 0.17 | 0.76 |
| 250 | Glyma.03G156400 | Glyma.19G158700 | 0.02 | 0.12 | 0.17 |
| 251 | Glyma.03G156500 | Glyma.19G158800 | 0.05 | 0.16 | 0.31 |
| 252 | Glyma.03G156600 | Glyma.19G158900 | 0.04 | 0.1 | 0.40 |
| 253 | Glyma.03G156700 | Glyma.19G159000 | 0.01 | 0.16 | 0.06 |
| 254 | Glyma.03G156800 | Glyma.19G159100 | 0.04 | 0.16 | 0.25 |
| 255 | Glyma.03G157000 | Glyma.19G159200 | 0.01 | 0.12 | 0.08 |
| 256 | Glyma.03G157100 | Glyma.19G159300 | 0.28 | 0.52 | 0.54 |
| 257 | Glyma.03G157200 | Glyma.19G159400 | 0.02 | 0.11 | 0.18 |
| 258 | Glyma.03G157300 | Glyma.19G159500 | 0.04 | 0.1 | 0.40 |
| 259 | Glyma.03G157400 | Glyma.19G159600 | 0.1 | 0.26 | 0.38 |
| 260 | Glyma.03G157700 | Glyma.19G159900 | 0.02 | 0.13 | 0.15 |
| 261 | Glyma.03G157800 | Glyma.19G160100 | 0.03 | 0.05 | 0.60 |
| 262 | Glyma.03G157900 | Glyma.19G160200 | 0.04 | 0.17 | 0.24 |
| 263 | Glyma.03G158000 | Glyma.19G160300 | 0.14 | 0.2 | 0.70 |
| 264 | Glyma.03G158100 | Glyma.19G160400 | 0 | 0.15 | 0.00 |
| 265 | Glyma.03G158200 | Glyma.19G160500 | 0.17 | 0.3 | 0.57 |
| 266 | Glyma.03G158300 | Glyma.19G160700 | 0.01 | 0.11 | 0.09 |
| 267 | Glyma.03G158400 | Glyma.19G160800 | 0.03 | 0.1 | 0.30 |
| 268 | Glyma.03G158500 | Glyma.19G160900 | 0.02 | 0.1 | 0.20 |

| | | | | | |
|---|---|---|---|---|---|
| 269 | Glyma.03G158600 | Glyma.19G161000 | 0.03 | 0.07 | 0.43 |
| 270 | Glyma.03G158700 | Glyma.19G161100 | 0.02 | 0.11 | 0.18 |
| 271 | Glyma.03G158800 | Glyma.19G161200 | 0.05 | 0.05 | 1.00 |
| 272 | Glyma.03G158900 | Glyma.19G161300 | 0.04 | 0.18 | 0.22 |
| 273 | Glyma.03G159100 | Glyma.19G161400 | 0.03 | 0.12 | 0.25 |
| 274 | Glyma.03G159200 | Glyma.19G161500 | 0.04 | 0.09 | 0.44 |
| 275 | Glyma.03G159400 | Glyma.19G161600 | 0.01 | 0.12 | 0.08 |
| 276 | Glyma.03G159500 | Glyma.19G161700 | 0.03 | 0.17 | 0.18 |
| 277 | Glyma.03G159600 | Glyma.19G161800 | 0.06 | 0.11 | 0.55 |
| 278 | Glyma.03G159900 | Glyma.19G161900 | 0.02 | 0.13 | 0.15 |
| 279 | Glyma.03G160000 | Glyma.19G162000 | 0.02 | 0.13 | 0.15 |
| 280 | Glyma.03G160100 | Glyma.19G162100 | 0.08 | 0.4 | 0.20 |
| 281 | Glyma.03G160400 | Glyma.19G162200 | 0.03 | 0.18 | 0.17 |
| 282 | Glyma.03G160600 | Glyma.19G162400 | 0.03 | 0.17 | 0.18 |
| 283 | Glyma.03G160700 | Glyma.19G162500 | 0.05 | 0.12 | 0.42 |
| 284 | Glyma.03G160900 | Glyma.19G162600 | 0.06 | 0.12 | 0.50 |
| 285 | Glyma.03G161000 | Glyma.19G162700 | 0.18 | 0.28 | 0.64 |
| 286 | Glyma.03G161100 | Glyma.19G162800 | 0.35 | 0.59 | 0.59 |
| 287 | Glyma.03G161400 | Glyma.19G162900 | 0.03 | 0.13 | 0.23 |
| 288 | Glyma.03G161600 | Glyma.19G163200 | 0.03 | 0.09 | 0.33 |
| 289 | Glyma.03G161800 | Glyma.19G163300 | 0.03 | 0.08 | 0.38 |
| 290 | Glyma.03G162000 | Glyma.19G163400 | 0.04 | 0.08 | 0.50 |
| 291 | Glyma.03G162100 | Glyma.19G163500 | 0.03 | 0.08 | 0.38 |
| 292 | Glyma.03G162200 | Glyma.19G163600 | 1.16 | 2.36 | 0.49 |
| 293 | Glyma.03G162400 | Glyma.19G163700 | 0.12 | 0.27 | 0.44 |
| 294 | Glyma.03G162500 | Glyma.19G163800 | 0.29 | 0.35 | 0.83 |
| 295 | Glyma.03G162600 | Glyma.19G164000 | 0.16 | 0.42 | 0.38 |
| 296 | Glyma.03G162800 | Glyma.19G164300 | 0 | 0.15 | 0.00 |
| 297 | Glyma.03G162900 | Glyma.19G164400 | 0.01 | 0.14 | 0.07 |
| 298 | Glyma.03G163000 | Glyma.19G164500 | 0.02 | 0.12 | 0.17 |
| 299 | Glyma.03G163100 | Glyma.19G164600 | 0.05 | 0.23 | 0.22 |
| 300 | Glyma.03G163200 | Glyma.19G164700 | 0.03 | 0.07 | 0.43 |
| 301 | Glyma.03G163400 | Glyma.19G164800 | 0.12 | 0.44 | 0.27 |
| 302 | Glyma.03G163600 | Glyma.19G165000 | 0.03 | 0.17 | 0.18 |
| 303 | Glyma.03G163800 | Glyma.19G165100 | 0.14 | 0.19 | 0.74 |
| 304 | Glyma.03G163900 | Glyma.19G165200 | 0.06 | 0.11 | 0.55 |
| 305 | Glyma.03G164000 | Glyma.19G165300 | 0.02 | 0.07 | 0.29 |
| 306 | Glyma.03G164100 | Glyma.19G165500 | 0.11 | 0.11 | 1.00 |
| 307 | Glyma.03G164200 | Glyma.19G165600 | 0.02 | 0.1 | 0.20 |
| 308 | Glyma.03G164300 | Glyma.19G165700 | 0.05 | 0.14 | 0.36 |
| 309 | Glyma.03G164400 | Glyma.19G165800 | 0.02 | 0.09 | 0.22 |
| 310 | Glyma.03G164500 | Glyma.19G165900 | 0.01 | 0.14 | 0.07 |
| 311 | Glyma.03G164600 | Glyma.19G166000 | 0.04 | 0.18 | 0.22 |
| 312 | Glyma.03G164700 | Glyma.19G166100 | 0.02 | 0.12 | 0.17 |
| 313 | Glyma.03G164800 | Glyma.19G166200 | 0.02 | 0.08 | 0.25 |

| | | | | | |
|---|---|---|---|---|---|
| 314 | Glyma.03G164900 | Glyma.19G166300 | 0.09 | 0.18 | 0.50 |
| 315 | Glyma.03G165000 | Glyma.19G166400 | 0.02 | 0.12 | 0.17 |
| 316 | Glyma.03G165200 | Glyma.19G166500 | 0.07 | 0.15 | 0.47 |
| 317 | Glyma.03G165400 | Glyma.19G166600 | 0.02 | 0.09 | 0.22 |
| 318 | Glyma.03G165500 | Glyma.19G166800 | 0.01 | 0.08 | 0.13 |
| 319 | Glyma.03G165600 | Glyma.19G166900 | 0.03 | 0.14 | 0.21 |
| 320 | Glyma.03G165900 | Glyma.19G167000 | 0.16 | 0.41 | 0.39 |
| 321 | Glyma.03G166000 | Glyma.19G167200 | 0.04 | 0.13 | 0.31 |
| 322 | Glyma.03G166400 | Glyma.19G167500 | 0.03 | 0.08 | 0.38 |
| 323 | Glyma.03G166500 | Glyma.19G167600 | 0.02 | 0.08 | 0.25 |
| 324 | Glyma.03G166600 | Glyma.19G167700 | 0.04 | 0.06 | 0.67 |
| 325 | Glyma.03G166700 | Glyma.19G167800 | 0 | 0.18 | 0.00 |
| 326 | Glyma.03G166800 | Glyma.19G167900 | 0.04 | 0.09 | 0.44 |
| 327 | Glyma.03G166900 | Glyma.19G168000 | 0.04 | 0.2 | 0.20 |
| 328 | Glyma.03G167000 | Glyma.19G168100 | 0.02 | 0.13 | 0.15 |
| 329 | Glyma.03G167100 | Glyma.19G168200 | 0.02 | 0.09 | 0.22 |
| 330 | Glyma.03G167200 | Glyma.19G168300 | 0.03 | 0.17 | 0.18 |
| 331 | Glyma.03G167300 | Glyma.19G168400 | 0.04 | 0.08 | 0.50 |
| 332 | Glyma.03G167500 | Glyma.19G168600 | 0.03 | 0.13 | 0.23 |
| 333 | Glyma.03G167600 | Glyma.19G168700 | 0.04 | 0.19 | 0.21 |
| 334 | Glyma.03G167700 | Glyma.19G168900 | 0.06 | 0.12 | 0.50 |
| 335 | Glyma.03G167800 | Glyma.19G169000 | 0.02 | 0.08 | 0.25 |
| 336 | Glyma.03G167900 | Glyma.19G169100 | 0.27 | 0.34 | 0.79 |
| 337 | Glyma.03G168000 | Glyma.19G169200 | 0.06 | 0.28 | 0.21 |
| 338 | Glyma.03G168100 | Glyma.19G169300 | 0.17 | 0.62 | 0.27 |
| 339 | Glyma.03G168400 | Glyma.19G169500 | 0.39 | 0.86 | 0.45 |
| 340 | Glyma.03G168500 | Glyma.19G169900 | 0.13 | 0.21 | 0.62 |
| 341 | Glyma.03G168600 | Glyma.19G170000 | 0.03 | 0.11 | 0.27 |
| 342 | Glyma.03G168700 | Glyma.19G170100 | 0 | 0.16 | 0.00 |
| 343 | Glyma.03G168800 | Glyma.19G170200 | 0.05 | 0.11 | 0.45 |
| 344 | Glyma.03G169000 | Glyma.19G170300 | 0.02 | 0.09 | 0.22 |
| 345 | Glyma.03G169100 | Glyma.19G170400 | 0.02 | 0.11 | 0.18 |
| 346 | Glyma.03G169200 | Glyma.19G170500 | 0.01 | 0.11 | 0.09 |
| 347 | Glyma.03G169300 | Glyma.19G170600 | 0.16 | 0.26 | 0.62 |
| 348 | Glyma.03G169500 | Glyma.19G170700 | 0.01 | 0.08 | 0.13 |
| 349 | Glyma.03G169600 | Glyma.19G170800 | 0.02 | 0.11 | 0.18 |
| 350 | Glyma.03G169700 | Glyma.19G170900 | 0.03 | 0.1 | 0.30 |
| 351 | Glyma.03G169800 | Glyma.19G171000 | 0.13 | 0.31 | 0.42 |
| 352 | Glyma.03G169900 | Glyma.19G171100 | 0.05 | 0.08 | 0.63 |
| 353 | Glyma.03G170000 | Glyma.19G171200 | 0.01 | 0.07 | 0.14 |
| 354 | Glyma.03G170100 | Glyma.19G171300 | 0.08 | 0.15 | 0.53 |
| 355 | Glyma.03G170400 | Glyma.19G171500 | 0.14 | 0.37 | 0.38 |
| 356 | Glyma.03G170600 | Glyma.19G171700 | 0 | 0.2 | 0.00 |
| 357 | Glyma.03G170800 | Glyma.19G171900 | 0.1 | 0.41 | 0.24 |
| 358 | Glyma.03G171000 | Glyma.19G172100 | 0.02 | 0.17 | 0.12 |

| | | | | | |
|---|---|---|---|---|---|
| 359 | Glyma.03G171100 | Glyma.19G172200 | 0 | 0.21 | 0.00 |
| 360 | Glyma.03G171200 | Glyma.19G172300 | 0.06 | 0.15 | 0.40 |
| 361 | Glyma.03G171300 | Glyma.19G172400 | 0 | 0.2 | 0.00 |
| 362 | Glyma.03G171400 | Glyma.19G172500 | 0 | 0.37 | 0.00 |
| 363 | Glyma.03G171500 | Glyma.19G172600 | 0.41 | 3.23 | 0.13 |
| 364 | Glyma.03G171800 | Glyma.19G172700 | 0.03 | 0.14 | 0.21 |
| 365 | Glyma.03G171900 | Glyma.19G172800 | 0.01 | 0.14 | 0.07 |
| 366 | Glyma.03G172000 | Glyma.19G172900 | 0.06 | 0.17 | 0.35 |
| 367 | Glyma.03G172100 | Glyma.19G173000 | 0.03 | 0.08 | 0.38 |
| 368 | Glyma.03G172200 | Glyma.19G173100 | 0.01 | 0.16 | 0.06 |
| 369 | Glyma.03G172300 | Glyma.19G173200 | 0.03 | 0.15 | 0.20 |
| 370 | Glyma.03G172400 | Glyma.19G173300 | 0.05 | 0.12 | 0.42 |
| 371 | Glyma.03G172500 | Glyma.19G173400 | 0.01 | 0.35 | 0.03 |
| 372 | Glyma.03G172600 | Glyma.19G173500 | 0.06 | 0.29 | 0.21 |
| 373 | Glyma.03G172700 | Glyma.19G173600 | 0.01 | 0.18 | 0.06 |
| 374 | Glyma.03G172900 | Glyma.19G173800 | 0.02 | 0.09 | 0.22 |
| 375 | Glyma.03G173100 | Glyma.19G174000 | 0.04 | 0.04 | 1.00 |
| 376 | Glyma.03G173400 | Glyma.19G174300 | 0.02 | 0.13 | 0.15 |
| 377 | Glyma.03G173500 | Glyma.19G174400 | 0.03 | 0.09 | 0.33 |
| 378 | Glyma.03G173600 | Glyma.19G174500 | 0.04 | 0.14 | 0.29 |
| 379 | Glyma.03G173700 | Glyma.19G174600 | 0.02 | 0.03 | 0.67 |
| 380 | Glyma.03G173800 | Glyma.19G174700 | 0.07 | 0.28 | 0.25 |
| 381 | Glyma.03G173900 | Glyma.19G174800 | 0.08 | 0.1 | 0.80 |
| 382 | Glyma.03G174100 | Glyma.19G175100 | 0.04 | 0.17 | 0.24 |
| 383 | Glyma.03G174300 | Glyma.19G175200 | 0.03 | 0.21 | 0.14 |
| 384 | Glyma.03G174400 | Glyma.19G175300 | 0.03 | 0.16 | 0.19 |
| 385 | Glyma.03G174500 | Glyma.19G175400 | 0.02 | 0.11 | 0.18 |
| 386 | Glyma.03G174700 | Glyma.19G175500 | 0.04 | 0.14 | 0.29 |
| 387 | Glyma.03G174900 | Glyma.19G175600 | 0.02 | 0.11 | 0.18 |
| 388 | Glyma.03G175000 | Glyma.19G175700 | 0.03 | 0.24 | 0.13 |
| 389 | Glyma.03G175100 | Glyma.19G175800 | 0.03 | 0.16 | 0.19 |
| 390 | Glyma.03G175200 | Glyma.19G175900 | 0.01 | 0.12 | 0.08 |
| 391 | Glyma.03G175300 | Glyma.19G176000 | 0.03 | 0.26 | 0.12 |
| 392 | Glyma.03G175400 | Glyma.19G176100 | 0.01 | 0.11 | 0.09 |
| 393 | Glyma.03G175700 | Glyma.19G176300 | 0.04 | 0.07 | 0.57 |
| 394 | Glyma.03G175900 | Glyma.19G176400 | 0.07 | 0.15 | 0.47 |
| 395 | Glyma.03G176000 | Glyma.19G176500 | 0.05 | 0.18 | 0.28 |
| 396 | Glyma.03G176100 | Glyma.19G176600 | 0.01 | 0.11 | 0.09 |
| 397 | Glyma.03G176200 | Glyma.19G176700 | 0.03 | 0.13 | 0.23 |
| 398 | Glyma.03G176300 | Glyma.19G176900 | 0.53 | 0.79 | 0.67 |
| 399 | Glyma.03G176400 | Glyma.19G177100 | 0.02 | 0.07 | 0.29 |
| 400 | Glyma.03G176500 | Glyma.19G177300 | 0.02 | 0.12 | 0.17 |
| 401 | Glyma.03G176600 | Glyma.19G177400 | 0.03 | 0.12 | 0.25 |
| 402 | Glyma.03G176800 | Glyma.19G177500 | 0.02 | 0.1 | 0.20 |
| 403 | Glyma.03G176900 | Glyma.19G177600 | 0.05 | 0.14 | 0.36 |

| | | | | | |
|---|---|---|---|---|---|
| 404 | Glyma.03G177000 | Glyma.19G177700 | 0.03 | 0.07 | 0.43 |
| 405 | Glyma.03G177100 | Glyma.19G177800 | 0.1 | 0.28 | 0.36 |
| 406 | Glyma.03G177200 | Glyma.19G177900 | 0.04 | 0.05 | 0.80 |
| 407 | Glyma.03G177300 | Glyma.19G178000 | 0.05 | 0.09 | 0.56 |
| 408 | Glyma.03G177400 | Glyma.19G178100 | 0.06 | 0.26 | 0.23 |
| 409 | Glyma.03G177500 | Glyma.19G178200 | 0.03 | 0.08 | 0.38 |
| 410 | Glyma.03G177600 | Glyma.19G178400 | 0.01 | 0.14 | 0.07 |
| 411 | Glyma.03G177700 | Glyma.19G178500 | 0.06 | 0.21 | 0.29 |
| 412 | Glyma.03G177800 | Glyma.19G178600 | 0.09 | 0.17 | 0.53 |
| 413 | Glyma.03G177900 | Glyma.19G178700 | 0.05 | 0.1 | 0.50 |
| 414 | Glyma.03G178000 | Glyma.19G178800 | 0 | 0.12 | 0.00 |
| 415 | Glyma.03G178100 | Glyma.19G178900 | 0.04 | 0.11 | 0.36 |
| 416 | Glyma.03G178200 | Glyma.19G179000 | 0.02 | 0.1 | 0.20 |
| 417 | Glyma.03G178300 | Glyma.19G179100 | 0.03 | 0.1 | 0.30 |
| 418 | Glyma.03G178400 | Glyma.19G179200 | 0.03 | 0.12 | 0.25 |
| 419 | Glyma.03G178500 | Glyma.19G179300 | 0.01 | 0.09 | 0.11 |
| 420 | Glyma.03G178700 | Glyma.19G179400 | 0.05 | 0.17 | 0.29 |
| 421 | Glyma.03G178800 | Glyma.19G179500 | 0.02 | 0.07 | 0.29 |
| 422 | Glyma.03G178900 | Glyma.19G179600 | 0.04 | 0.07 | 0.57 |
| 423 | Glyma.03G179000 | Glyma.19G179700 | 0.02 | 0.13 | 0.15 |
| 424 | Glyma.03G179100 | Glyma.19G179800 | 0.03 | 0.08 | 0.38 |
| 425 | Glyma.03G179200 | Glyma.19G179900 | 0.02 | 0.1 | 0.20 |
| 426 | Glyma.03G179300 | Glyma.19G180000 | 0.04 | 0.06 | 0.67 |
| 427 | Glyma.03G179400 | Glyma.19G180100 | 0.07 | 0.06 | 1.17 |
| 428 | Glyma.03G179500 | Glyma.19G180200 | 0.02 | 0.08 | 0.25 |
| 429 | Glyma.03G179600 | Glyma.19G180300 | 0.04 | 0.14 | 0.29 |
| 430 | Glyma.03G179700 | Glyma.19G180400 | 0.05 | 0.19 | 0.26 |
| 431 | Glyma.03G179800 | Glyma.19G180600 | 0.01 | 0.11 | 0.09 |
| 432 | Glyma.03G179900 | Glyma.19G180700 | 0.01 | 0.19 | 0.05 |
| 433 | Glyma.03G180100 | Glyma.19G180800 | 0.02 | 0.16 | 0.13 |
| 434 | Glyma.03G180200 | Glyma.19G180900 | 0.05 | 0.17 | 0.29 |
| 435 | Glyma.03G180300 | Glyma.19G181000 | 0.06 | 0.16 | 0.38 |
| 436 | Glyma.03G180500 | Glyma.19G181100 | 0.05 | 0.19 | 0.26 |
| 437 | Glyma.03G180600 | Glyma.19G181200 | 0.08 | 0.14 | 0.57 |
| 438 | Glyma.03G180900 | Glyma.19G181300 | 0.02 | 0.2 | 0.10 |
| 439 | Glyma.03G181000 | Glyma.19G181700 | 0.04 | 0.16 | 0.25 |
| 440 | Glyma.03G181100 | Glyma.19G181800 | 0.03 | 0.21 | 0.14 |
| 441 | Glyma.03G181200 | Glyma.19G182000 | 0.02 | 0.17 | 0.12 |
| 442 | Glyma.03G181300 | Glyma.19G182100 | 0.01 | 0.09 | 0.11 |
| 443 | Glyma.03G181600 | Glyma.19G182300 | 0.01 | 0.16 | 0.06 |
| 444 | Glyma.03G181900 | Glyma.19G182400 | 0.03 | 0.08 | 0.38 |
| 445 | Glyma.03G182000 | Glyma.19G182500 | 0.02 | 0.18 | 0.11 |
| 446 | Glyma.03G182100 | Glyma.19G182600 | 0.02 | 0.16 | 0.13 |
| 447 | Glyma.03G182200 | Glyma.19G182800 | 0.06 | 0.18 | 0.33 |
| 448 | Glyma.03G182300 | Glyma.19G182900 | 0.3 | 0.47 | 0.64 |

| | | | | | |
|---|---|---|---|---|---|
| 449 | Glyma.03G182400 | Glyma.19G183000 | 0.03 | 0.14 | 0.21 |
| 450 | Glyma.03G182600 | Glyma.19G183100 | 0.03 | 0.1 | 0.30 |
| 451 | Glyma.03G182700 | Glyma.19G183200 | 0 | 0.16 | 0.00 |
| 452 | Glyma.03G182800 | Glyma.19G183400 | 0.01 | 0.16 | 0.06 |
| 453 | Glyma.03G182900 | Glyma.19G183500 | 0.04 | 0.16 | 0.25 |
| 454 | Glyma.03G183000 | Glyma.19G183700 | 0.22 | 0.46 | 0.48 |
| 455 | Glyma.03G183300 | Glyma.19G184100 | 0.05 | 0.16 | 0.31 |
| 456 | Glyma.03G183500 | Glyma.19G184200 | 0.01 | 0.08 | 0.13 |
| 457 | Glyma.03G183600 | Glyma.19G184300 | 0.01 | 0.14 | 0.07 |
| 458 | Glyma.03G183900 | Glyma.19G184500 | 0.04 | 0.16 | 0.25 |
| 459 | Glyma.03G184100 | Glyma.19G184600 | 0.19 | 0.27 | 0.70 |
| 460 | Glyma.03G184200 | Glyma.19G184800 | 0.02 | 0.07 | 0.29 |
| 461 | Glyma.03G184500 | Glyma.19G184900 | 0.02 | 0.07 | 0.29 |
| 462 | Glyma.03G184600 | Glyma.19G185000 | 0.06 | 0.1 | 0.60 |
| 463 | Glyma.03G184700 | Glyma.19G185100 | 0.21 | 0.39 | 0.54 |
| 464 | Glyma.03G184800 | Glyma.19G185300 | 0.02 | 0.1 | 0.20 |
| 465 | Glyma.03G184900 | Glyma.19G185400 | 0.03 | 0.09 | 0.33 |
| 466 | Glyma.03G185000 | Glyma.19G185500 | 0.05 | 0.24 | 0.21 |
| 467 | Glyma.03G185200 | Glyma.19G185700 | 0.01 | 0.17 | 0.06 |
| 468 | Glyma.03G185300 | Glyma.19G185800 | 0.12 | 0.22 | 0.55 |
| 469 | Glyma.03G185500 | Glyma.19G185900 | 0.06 | 0.15 | 0.40 |
| 470 | Glyma.03G185800 | Glyma.19G186000 | 0.01 | 0.15 | 0.07 |
| 471 | Glyma.03G185900 | Glyma.19G186100 | 0.01 | 0.18 | 0.06 |
| 472 | Glyma.03G186000 | Glyma.19G186200 | 0.04 | 0.07 | 0.57 |
| 473 | Glyma.03G186200 | Glyma.19G186300 | 0 | 0.06 | 0.00 |
| 474 | Glyma.03G186300 | Glyma.19G186400 | 0.02 | 0.12 | 0.17 |
| 475 | Glyma.03G186400 | Glyma.19G186500 | 0.05 | 0.1 | 0.50 |
| 476 | Glyma.03G186500 | Glyma.19G186600 | 0.01 | 0.11 | 0.09 |
| 477 | Glyma.03G186600 | Glyma.19G186700 | 0.03 | 0.1 | 0.30 |
| 478 | Glyma.03G186700 | Glyma.19G186800 | 0.2 | 0.18 | 1.11 |
| 479 | Glyma.03G186800 | Glyma.19G186900 | 0.08 | 0.15 | 0.53 |
| 480 | Glyma.03G186900 | Glyma.19G187000 | 0.06 | 0.21 | 0.29 |
| 481 | Glyma.03G187100 | Glyma.19G187100 | 0.12 | 0.19 | 0.63 |
| 482 | Glyma.03G187600 | Glyma.19G187500 | 0.77 | 0 | #DIV/0! |
| 483 | Glyma.03G187800 | Glyma.19G187900 | 0.26 | 0.54 | 0.48 |
| 484 | Glyma.03G187900 | Glyma.19G188100 | 0.03 | 0.11 | 0.27 |
| 485 | Glyma.03G188000 | Glyma.19G188200 | 0.03 | 0.1 | 0.30 |
| 486 | Glyma.03G188100 | Glyma.19G188300 | 0.01 | 0.13 | 0.08 |
| 487 | Glyma.03G188300 | Glyma.19G188400 | 0.03 | 0.14 | 0.21 |
| 488 | Glyma.03G188400 | Glyma.19G188600 | 0.02 | 0.1 | 0.20 |
| 489 | Glyma.03G188500 | Glyma.19G188700 | 0.02 | 0.09 | 0.22 |
| 490 | Glyma.03G188600 | Glyma.19G188800 | 0.02 | 0.11 | 0.18 |
| 491 | Glyma.03G188700 | Glyma.19G188900 | 0.02 | 0.13 | 0.15 |
| 492 | Glyma.03G188800 | Glyma.19G189000 | 0.04 | 0.12 | 0.33 |
| 493 | Glyma.03G188900 | Glyma.19G189100 | 0.03 | 0.07 | 0.43 |

| | | | | | |
|---|---|---|---|---|---|
| 494 | Glyma.03G189000 | Glyma.19G189200 | 0.06 | 0.11 | 0.55 |
| 495 | Glyma.03G189100 | Glyma.19G189300 | 0.03 | 0.16 | 0.19 |
| 496 | Glyma.03G189200 | Glyma.19G189400 | 0.14 | 0.5 | 0.28 |
| 497 | Glyma.03G189300 | Glyma.19G189600 | 0.01 | 0.14 | 0.07 |
| 498 | Glyma.03G189400 | Glyma.19G189700 | 0.04 | 0.15 | 0.27 |
| 499 | Glyma.03G189600 | Glyma.19G190000 | 0.03 | 0.17 | 0.18 |
| 500 | Glyma.03G189700 | Glyma.19G190100 | 0.01 | 0.12 | 0.08 |
| 501 | Glyma.03G189800 | Glyma.19G190200 | 0.03 | 0.24 | 0.13 |
| 502 | Glyma.03G190000 | Glyma.19G190300 | 0.04 | 0.14 | 0.29 |
| 503 | Glyma.03G190100 | Glyma.19G190400 | 0.08 | 0.2 | 0.40 |
| 504 | Glyma.03G190200 | Glyma.19G190600 | 0.01 | 0.07 | 0.14 |
| 505 | Glyma.03G190300 | Glyma.19G190700 | 0.03 | 0.12 | 0.25 |
| 506 | Glyma.03G190400 | Glyma.19G190800 | 0.06 | 0.25 | 0.24 |
| 507 | Glyma.03G190500 | Glyma.19G190900 | 0.01 | 0.11 | 0.09 |
| 508 | Glyma.03G190600 | Glyma.19G191100 | 0.01 | 0.19 | 0.05 |
| 509 | Glyma.03G190800 | Glyma.19G191300 | 0.15 | 0.24 | 0.63 |
| 510 | Glyma.03G191000 | Glyma.19G191600 | 0.02 | 0.1 | 0.20 |
| 511 | Glyma.03G191100 | Glyma.19G191700 | 0.1 | 0.27 | 0.37 |
| 512 | Glyma.03G191200 | Glyma.19G191800 | 0.05 | 0.15 | 0.33 |
| 513 | Glyma.03G191300 | Glyma.19G192000 | 0.07 | 0.2 | 0.35 |
| 514 | Glyma.03G191400 | Glyma.19G192100 | 0.05 | 0.2 | 0.25 |
| 515 | Glyma.03G191700 | Glyma.19G192300 | 0.02 | 0.15 | 0.13 |
| 516 | Glyma.03G191800 | Glyma.19G192400 | 0.03 | 0.18 | 0.17 |
| 517 | Glyma.03G192000 | Glyma.19G192500 | 0.05 | 0.14 | 0.36 |
| 518 | Glyma.03G192100 | Glyma.19G192600 | 0.01 | 0.11 | 0.09 |
| 519 | Glyma.03G192200 | Glyma.19G192700 | 0.03 | 0.12 | 0.25 |
| 520 | Glyma.03G192300 | Glyma.19G192800 | 0.05 | 0.13 | 0.38 |
| 521 | Glyma.03G192500 | Glyma.19G192900 | 0.12 | 0.4 | 0.30 |
| 522 | Glyma.03G192900 | Glyma.19G193000 | 0.07 | 0.21 | 0.33 |
| 523 | Glyma.03G193000 | Glyma.19G193100 | 0.02 | 0.09 | 0.22 |
| 524 | Glyma.03G193100 | Glyma.19G193200 | 0.02 | 0.17 | 0.12 |
| 525 | Glyma.03G193200 | Glyma.19G193300 | 0.25 | 0.47 | 0.53 |
| 526 | Glyma.03G193300 | Glyma.19G193400 | 0.06 | 0.13 | 0.46 |
| 527 | Glyma.03G193600 | Glyma.19G193500 | 0.04 | 0.16 | 0.25 |
| 528 | Glyma.03G193700 | Glyma.19G193600 | 0.06 | 0.23 | 0.26 |
| 529 | Glyma.03G193800 | Glyma.19G193700 | 0.06 | 0.15 | 0.40 |
| 530 | Glyma.03G194000 | Glyma.19G193800 | 0.03 | 0.13 | 0.23 |
| 531 | Glyma.03G194100 | Glyma.19G193900 | 0.04 | 0.15 | 0.27 |
| 532 | Glyma.03G194300 | Glyma.19G194100 | 0.03 | 0.06 | 0.50 |
| 533 | Glyma.03G194400 | Glyma.19G194200 | 0.01 | 0.14 | 0.07 |
| 534 | Glyma.03G194700 | Glyma.19G194300 | 0.01 | 0.03 | 0.33 |
| 535 | Glyma.03G194800 | Glyma.19G194400 | 0.06 | 0.1 | 0.60 |
| 536 | Glyma.03G194900 | Glyma.19G194500 | 0.04 | 0.25 | 0.16 |
| 537 | Glyma.03G195000 | Glyma.19G194700 | 0.16 | 0.3 | 0.53 |
| 538 | Glyma.03G195200 | Glyma.19G194800 | 0.01 | 0.14 | 0.07 |

| | | | | | |
|---|---|---|---|---|---|
| 539 | Glyma.03G195300 | Glyma.19G194900 | 0.12 | 0.17 | 0.71 |
| 540 | Glyma.03G197100 | Glyma.19G195100 | 0 | 0.15 | 0.00 |
| 541 | Glyma.03G197200 | Glyma.19G195200 | 0.04 | 0.16 | 0.25 |
| 542 | Glyma.03G197300 | Glyma.19G195300 | 0.02 | 0.11 | 0.18 |
| 543 | Glyma.03G197400 | Glyma.19G195400 | 0.03 | 0.22 | 0.14 |
| 544 | Glyma.03G197600 | Glyma.19G195500 | 0 | 0.25 | 0.00 |
| 545 | Glyma.03G197700 | Glyma.19G195600 | 0.03 | 0.13 | 0.23 |
| 546 | Glyma.03G197800 | Glyma.19G195700 | 0.04 | 0.16 | 0.25 |
| 547 | Glyma.03G197900 | Glyma.19G195800 | 0.06 | 0.12 | 0.50 |
| 548 | Glyma.03G198000 | Glyma.19G195900 | 0.01 | 0.07 | 0.14 |
| 549 | Glyma.03G198100 | Glyma.19G196000 | 0.01 | 0.1 | 0.10 |
| 550 | Glyma.03G198200 | Glyma.19G196100 | 0.01 | 0.1 | 0.10 |
| 551 | Glyma.03G198300 | Glyma.19G196200 | 0.04 | 0.12 | 0.33 |
| 552 | Glyma.03G198400 | Glyma.19G196300 | 0.03 | 0.11 | 0.27 |
| 553 | Glyma.03G198600 | Glyma.19G196400 | 0.09 | 0.2 | 0.45 |
| 554 | Glyma.03G198800 | Glyma.19G196500 | 0.02 | 0.09 | 0.22 |
| 555 | Glyma.03G199000 | Glyma.19G196600 | 0.07 | 0.27 | 0.26 |
| 556 | Glyma.03G199100 | Glyma.19G196700 | 0.02 | 0.12 | 0.17 |
| 557 | Glyma.03G199500 | Glyma.19G196800 | 0.02 | 0.09 | 0.22 |
| 558 | Glyma.03G199600 | Glyma.19G196900 | 0.43 | 3.03 | 0.14 |
| 559 | Glyma.03G199700 | Glyma.19G197300 | 0.02 | 0.08 | 0.25 |
| 560 | Glyma.03G199800 | Glyma.19G197400 | 0 | 0.07 | 0.00 |
| 561 | Glyma.03G199900 | Glyma.19G197500 | 0.02 | 0.14 | 0.14 |
| 562 | Glyma.03G200000 | Glyma.19G197600 | 0.02 | 0.27 | 0.07 |
| 563 | Glyma.03G200100 | Glyma.19G197700 | 0.06 | 0.18 | 0.33 |
| 564 | Glyma.03G200200 | Glyma.19G197800 | 0.03 | 0.13 | 0.23 |
| 565 | Glyma.03G200300 | Glyma.19G197900 | 0.05 | 0.13 | 0.38 |
| 566 | Glyma.03G200400 | Glyma.19G198000 | 0.01 | 0.08 | 0.13 |
| 567 | Glyma.03G200500 | Glyma.19G198100 | 0.07 | 0.11 | 0.64 |
| 568 | Glyma.03G200600 | Glyma.19G198200 | 0.48 | 0.65 | 0.74 |
| 569 | Glyma.03G200700 | Glyma.19G198300 | 0.12 | 0.26 | 0.46 |
| 570 | Glyma.03G200800 | Glyma.19G198400 | 0 | 0.06 | 0.00 |
| 571 | Glyma.03G200900 | Glyma.19G198500 | 0.05 | 0.22 | 0.23 |
| 572 | Glyma.03G201000 | Glyma.19G198600 | 0.03 | 0.21 | 0.14 |
| 573 | Glyma.03G201600 | Glyma.19G198900 | 0.02 | 0.13 | 0.15 |
| 574 | Glyma.03G201800 | Glyma.19G199100 | 0.22 | 0.59 | 0.37 |
| 575 | Glyma.03G202200 | Glyma.19G199900 | 0.03 | 0.12 | 0.25 |
| 576 | Glyma.03G202600 | Glyma.19G200200 | 0.03 | 0.17 | 0.18 |
| 577 | Glyma.03G202700 | Glyma.19G200400 | 0.02 | 0.08 | 0.25 |
| 578 | Glyma.03G202800 | Glyma.19G200500 | 0.04 | 0.18 | 0.22 |
| 579 | Glyma.03G202900 | Glyma.19G200700 | 0.03 | 0.11 | 0.27 |
| 580 | Glyma.03G203000 | Glyma.19G200800 | 0.04 | 0.1 | 0.40 |
| 581 | Glyma.03G203500 | Glyma.19G200900 | 0.03 | 0.16 | 0.19 |
| 582 | Glyma.03G203600 | Glyma.19G201000 | 0.07 | 0.15 | 0.47 |
| 583 | Glyma.03G203700 | Glyma.19G201100 | 0.03 | 0.09 | 0.33 |

| | | | | | |
|---|---|---|---|---|---|
| 584 | Glyma.03G203800 | Glyma.19G201200 | 0.07 | 0.22 | 0.32 |
| 585 | Glyma.03G203900 | Glyma.19G201300 | 0.04 | 0.11 | 0.36 |
| 586 | Glyma.03G204100 | Glyma.19G201400 | 0.04 | 0.12 | 0.33 |
| 587 | Glyma.03G204200 | Glyma.19G201500 | 0.05 | 0.13 | 0.38 |
| 588 | Glyma.03G204300 | Glyma.19G201600 | 0.02 | 0.21 | 0.10 |
| 589 | Glyma.03G204400 | Glyma.19G201800 | 0.05 | 0.14 | 0.36 |
| 590 | Glyma.03G204500 | Glyma.19G201900 | 0.04 | 0.11 | 0.36 |
| 591 | Glyma.03G204600 | Glyma.19G202000 | 0.05 | 0.05 | 1.00 |
| 592 | Glyma.03G204800 | Glyma.19G202200 | 0.01 | 0.1 | 0.10 |
| 593 | Glyma.03G204900 | Glyma.19G202300 | 0.07 | 0.13 | 0.54 |
| 594 | Glyma.03G205000 | Glyma.19G202400 | 0.04 | 0.09 | 0.44 |
| 595 | Glyma.03G205100 | Glyma.19G202500 | 0.07 | 0.19 | 0.37 |
| 596 | Glyma.03G205200 | Glyma.19G202600 | 0.04 | 0.19 | 0.21 |
| 597 | Glyma.03G205300 | Glyma.19G202800 | 0.06 | 0.21 | 0.29 |
| 598 | Glyma.03G205400 | Glyma.19G202900 | 0.03 | 0.14 | 0.21 |
| 599 | Glyma.03G205600 | Glyma.19G203100 | 0.05 | 0.15 | 0.33 |
| 600 | Glyma.03G205700 | Glyma.19G203200 | 0.03 | 0.11 | 0.27 |
| 601 | Glyma.03G205800 | Glyma.19G203300 | 0 | 0.2 | 0.00 |
| 602 | Glyma.03G205900 | Glyma.19G203400 | 0.04 | 0.15 | 0.27 |
| 603 | Glyma.03G206000 | Glyma.19G203500 | 0.1 | 0.2 | 0.50 |
| 604 | Glyma.03G206100 | Glyma.19G203700 | 0.41 | 0.84 | 0.49 |
| 605 | Glyma.03G206200 | Glyma.19G204000 | 0.02 | 0.08 | 0.25 |
| 606 | Glyma.03G206500 | Glyma.19G204100 | 0.09 | 0.15 | 0.60 |
| 607 | Glyma.03G206600 | Glyma.19G204200 | 0.04 | 0.09 | 0.44 |
| 608 | Glyma.03G206800 | Glyma.19G204300 | 0.08 | 0.1 | 0.80 |
| 609 | Glyma.03G206900 | Glyma.19G204400 | 0 | 0.06 | 0.00 |
| 610 | Glyma.03G207000 | Glyma.19G204500 | 0.01 | 0.08 | 0.13 |
| 611 | Glyma.03G207100 | Glyma.19G204600 | 0.01 | 0.21 | 0.05 |
| 612 | Glyma.03G207200 | Glyma.19G204700 | 0.03 | 0.2 | 0.15 |
| 613 | Glyma.03G207300 | Glyma.19G204800 | 0.02 | 0.09 | 0.22 |
| 614 | Glyma.03G207400 | Glyma.19G204900 | 0.04 | 0.14 | 0.29 |
| 615 | Glyma.03G207500 | Glyma.19G205000 | 0.01 | 0.35 | 0.03 |
| 616 | Glyma.03G207700 | Glyma.19G205100 | 0 | 0.25 | 0.00 |
| 617 | Glyma.03G207900 | Glyma.19G205200 | 0.02 | 0.17 | 0.12 |
| 618 | Glyma.03G208100 | Glyma.19G205300 | 0.21 | 0.28 | 0.75 |
| 619 | Glyma.03G208300 | Glyma.19G205500 | 0.04 | 0.13 | 0.31 |
| 620 | Glyma.03G208400 | Glyma.19G205600 | 0.05 | 0.14 | 0.36 |
| 621 | Glyma.03G208500 | Glyma.19G205700 | 0.03 | 0.11 | 0.27 |
| 622 | Glyma.03G208600 | Glyma.19G205800 | 0.05 | 0.09 | 0.56 |
| 623 | Glyma.03G208800 | Glyma.19G206100 | 0.09 | 0.19 | 0.47 |
| 624 | Glyma.03G208900 | Glyma.19G206200 | 0.25 | 1.43 | 0.17 |
| 625 | Glyma.03G209100 | Glyma.19G206300 | 0.03 | 0.13 | 0.23 |
| 626 | Glyma.03G209400 | Glyma.19G206800 | 0.01 | 0.13 | 0.08 |
| 627 | Glyma.03G209500 | Glyma.19G206900 | 0.06 | 0.11 | 0.55 |
| 628 | Glyma.03G209700 | Glyma.19G207000 | 0.03 | 0.14 | 0.21 |

| | | | | | |
|---|---|---|---|---|---|
| 629 | Glyma.03G209800 | Glyma.19G207100 | 0.03 | 0.15 | 0.20 |
| 630 | Glyma.03G210000 | Glyma.19G207200 | 0.09 | 0.2 | 0.45 |
| 631 | Glyma.03G210100 | Glyma.19G207300 | 0.01 | 0.1 | 0.10 |
| 632 | Glyma.03G210300 | Glyma.19G207500 | 0.03 | 0.11 | 0.27 |
| 633 | Glyma.03G210400 | Glyma.19G207600 | 0.01 | 0.15 | 0.07 |
| 634 | Glyma.03G210600 | Glyma.19G207800 | 0.03 | 0.23 | 0.13 |
| 635 | Glyma.03G210700 | Glyma.19G207900 | 0.02 | 0.14 | 0.14 |
| 636 | Glyma.03G210800 | Glyma.19G208000 | 0.01 | 0.1 | 0.10 |
| 637 | Glyma.03G210900 | Glyma.19G208100 | 0.02 | 0.1 | 0.20 |
| 638 | Glyma.03G211000 | Glyma.19G208200 | 0.02 | 0.14 | 0.14 |
| 639 | Glyma.03G211100 | Glyma.19G208300 | 0.05 | 0.17 | 0.29 |
| 640 | Glyma.03G211200 | Glyma.19G208400 | 0.04 | 0.08 | 0.50 |
| 641 | Glyma.03G211400 | Glyma.19G208600 | 0.01 | 0.1 | 0.10 |
| 642 | Glyma.03G211500 | Glyma.19G208700 | 0.03 | 0.1 | 0.30 |
| 643 | Glyma.03G211700 | Glyma.19G208900 | 0.03 | 0.17 | 0.18 |
| 644 | Glyma.03G211800 | Glyma.19G209000 | 0.03 | 0.09 | 0.33 |
| 645 | Glyma.03G212000 | Glyma.19G209100 | 0.1 | 0.25 | 0.40 |
| 646 | Glyma.03G212100 | Glyma.19G209200 | 0.03 | 0.11 | 0.27 |
| 647 | Glyma.03G212300 | Glyma.19G209400 | 0.04 | 0.2 | 0.20 |
| 648 | Glyma.03G212400 | Glyma.19G209500 | 0.04 | 0.07 | 0.57 |
| 649 | Glyma.03G212500 | Glyma.19G209600 | 0.02 | 0.04 | 0.50 |
| 650 | Glyma.03G212700 | Glyma.19G209700 | 0.02 | 0.14 | 0.14 |
| 651 | Glyma.03G212900 | Glyma.19G209800 | 0.01 | 0.09 | 0.11 |
| 652 | Glyma.03G213000 | Glyma.19G209900 | 0.03 | 0.17 | 0.18 |
| 653 | Glyma.03G213200 | Glyma.19G210000 | 0.03 | 0.15 | 0.20 |
| 654 | Glyma.03G213300 | Glyma.19G210100 | 0.28 | 0.44 | 0.64 |
| 655 | Glyma.03G213400 | Glyma.19G210200 | 0.04 | 0.19 | 0.21 |
| 656 | Glyma.03G213500 | Glyma.19G210300 | 0.02 | 0.09 | 0.22 |
| 657 | Glyma.03G213600 | Glyma.19G210400 | 0.37 | 0.74 | 0.50 |
| 658 | Glyma.03G213700 | Glyma.19G210500 | 0.04 | 0.14 | 0.29 |
| 659 | Glyma.03G213800 | Glyma.19G210600 | 0.45 | 0.54 | 0.83 |
| 660 | Glyma.03G214000 | Glyma.19G210700 | 0.12 | 0.34 | 0.35 |
| 661 | Glyma.03G214100 | Glyma.19G210900 | 0.01 | 0.16 | 0.06 |
| 662 | Glyma.03G214400 | Glyma.19G211100 | 0.03 | 0.14 | 0.21 |
| 663 | Glyma.03G214500 | Glyma.19G211200 | 0.01 | 0.13 | 0.08 |
| 664 | Glyma.03G214700 | Glyma.19G211300 | 0.01 | 0.21 | 0.05 |
| 665 | Glyma.03G214900 | Glyma.19G211500 | 0.03 | 0.09 | 0.33 |
| 666 | Glyma.03G215000 | Glyma.19G211600 | 0.05 | 0.17 | 0.29 |
| 667 | Glyma.03G215100 | Glyma.19G211700 | 0.06 | 0.14 | 0.43 |
| 668 | Glyma.03G215300 | Glyma.19G211800 | 0.01 | 0.18 | 0.06 |
| 669 | Glyma.03G215400 | Glyma.19G211900 | 0.05 | 0.14 | 0.36 |
| 670 | Glyma.03G215500 | Glyma.19G212000 | 0.03 | 0.11 | 0.27 |
| 671 | Glyma.03G215600 | Glyma.19G212100 | 0.05 | 0.13 | 0.38 |
| 672 | Glyma.03G215700 | Glyma.19G212200 | 0.04 | 0.11 | 0.36 |
| 673 | Glyma.03G215800 | Glyma.19G212300 | 0.01 | 0.24 | 0.04 |

| | | | | | |
|---|---|---|---|---|---|
| 674 | Glyma.03G216100 | Glyma.19G212700 | 0.03 | 0.14 | 0.21 |
| 675 | Glyma.03G216300 | Glyma.19G212800 | 0.01 | 0.07 | 0.14 |
| 676 | Glyma.03G216400 | Glyma.19G212900 | 0.01 | 0.09 | 0.11 |
| 677 | Glyma.03G216500 | Glyma.19G213000 | 0.08 | 0.13 | 0.62 |
| 678 | Glyma.03G216600 | Glyma.19G213200 | 0.01 | 0.19 | 0.05 |
| 679 | Glyma.03G216700 | Glyma.19G213300 | 0.01 | 0.15 | 0.07 |
| 680 | Glyma.03G216800 | Glyma.19G213400 | 0.03 | 0.1 | 0.30 |
| 681 | Glyma.03G216900 | Glyma.19G213500 | 0.06 | 0.12 | 0.50 |
| 682 | Glyma.03G217000 | Glyma.19G213600 | 0.02 | 0.1 | 0.20 |
| 683 | Glyma.03G217100 | Glyma.19G213700 | 0.02 | 0.13 | 0.15 |
| 684 | Glyma.03G217200 | Glyma.19G213800 | 0.05 | 0.14 | 0.36 |
| 685 | Glyma.03G217300 | Glyma.19G213900 | 0.05 | 0.13 | 0.38 |
| 686 | Glyma.03G217400 | Glyma.19G214000 | 0.02 | 0.12 | 0.17 |
| 687 | Glyma.03G217500 | Glyma.19G214100 | 0.02 | 0.21 | 0.10 |
| 688 | Glyma.03G217600 | Glyma.19G214200 | 0.01 | 0.15 | 0.07 |
| 689 | Glyma.03G217700 | Glyma.19G214300 | 0.04 | 0.15 | 0.27 |
| 690 | Glyma.03G217800 | Glyma.19G214400 | 0.1 | 0.27 | 0.37 |
| 691 | Glyma.03G217900 | Glyma.19G214600 | 0.09 | 0.14 | 0.64 |
| 692 | Glyma.03G218000 | Glyma.19G214700 | 0.02 | 0.26 | 0.08 |
| 693 | Glyma.03G218100 | Glyma.19G214800 | 0.06 | 0.22 | 0.27 |
| 694 | Glyma.03G218200 | Glyma.19G214900 | 0.1 | 0.19 | 0.53 |
| 695 | Glyma.03G218300 | Glyma.19G215100 | 0.01 | 0.12 | 0.08 |
| 696 | Glyma.03G218400 | Glyma.19G215200 | 0.05 | 0.09 | 0.56 |
| 697 | Glyma.03G218500 | Glyma.19G215300 | 0.03 | 0.1 | 0.30 |
| 698 | Glyma.03G218600 | Glyma.19G215400 | 0.01 | 0.16 | 0.06 |
| 699 | Glyma.03G218700 | Glyma.19G215500 | 0.03 | 0.2 | 0.15 |
| 700 | Glyma.03G218800 | Glyma.19G215600 | 0.04 | 0.12 | 0.33 |
| 701 | Glyma.03G218900 | Glyma.19G215700 | 0.18 | 0.25 | 0.72 |
| 702 | Glyma.03G219000 | Glyma.19G215900 | 0.04 | 0.11 | 0.36 |
| 703 | Glyma.03G219200 | Glyma.19G216100 | 0.01 | 0.09 | 0.11 |
| 704 | Glyma.03G219300 | Glyma.19G216200 | 0.02 | 0.17 | 0.12 |
| 705 | Glyma.03G219400 | Glyma.19G216300 | 0.05 | 0.16 | 0.31 |
| 706 | Glyma.03G219500 | Glyma.19G216400 | 0.02 | 0.11 | 0.18 |
| 707 | Glyma.03G219700 | Glyma.19G216500 | 0.03 | 0.1 | 0.30 |
| 708 | Glyma.03G219800 | Glyma.19G216600 | 0.11 | 0.25 | 0.44 |
| 709 | Glyma.03G219900 | Glyma.19G216700 | 0.05 | 0.14 | 0.36 |
| 710 | Glyma.03G220000 | Glyma.19G216900 | 0.05 | 0.23 | 0.22 |
| 711 | Glyma.03G220100 | Glyma.19G217000 | 0.02 | 0.14 | 0.14 |
| 712 | Glyma.03G220200 | Glyma.19G217100 | 0.53 | 0.82 | 0.65 |
| 713 | Glyma.03G220300 | Glyma.19G217200 | 0.01 | 0.11 | 0.09 |
| 714 | Glyma.03G220500 | Glyma.19G217300 | 0.03 | 0.09 | 0.33 |
| 715 | Glyma.03G220600 | Glyma.19G217400 | 0.01 | 0.13 | 0.08 |
| 716 | Glyma.03G220700 | Glyma.19G217500 | 0.02 | 0.08 | 0.25 |
| 717 | Glyma.03G220800 | Glyma.19G217800 | 0.04 | 0.13 | 0.31 |
| 718 | Glyma.03G220900 | Glyma.19G217900 | 0.05 | 0.12 | 0.42 |

| | | | | | |
|---|---|---|---|---|---|
| 719 | Glyma.03G221000 | Glyma.19G218000 | 0.02 | 0.09 | 0.22 |
| 720 | Glyma.03G221100 | Glyma.19G218100 | 0.06 | 0.15 | 0.40 |
| 721 | Glyma.03G221200 | Glyma.19G218200 | 0 | 0.11 | 0.00 |
| 722 | Glyma.03G221400 | Glyma.19G218300 | 0.02 | 0.13 | 0.15 |
| 723 | Glyma.03G221500 | Glyma.19G218500 | 0.02 | 0.11 | 0.18 |
| 724 | Glyma.03G221600 | Glyma.19G218700 | 0.03 | 0.1 | 0.30 |
| 725 | Glyma.03G221700 | Glyma.19G218800 | 0.09 | 0.21 | 0.43 |
| 726 | Glyma.03G222000 | Glyma.19G219100 | 0.01 | 0.17 | 0.06 |
| 727 | Glyma.03G222100 | Glyma.19G219300 | 0.03 | 0.2 | 0.15 |
| 728 | Glyma.03G222200 | Glyma.19G219400 | 0.04 | 0.15 | 0.27 |
| 729 | Glyma.03G222300 | Glyma.19G219500 | 0.01 | 0.11 | 0.09 |
| 730 | Glyma.03G222400 | Glyma.19G219600 | 0.12 | 0.17 | 0.71 |
| 731 | Glyma.03G222500 | Glyma.19G219700 | 0.01 | 0.09 | 0.11 |
| 732 | Glyma.03G222600 | Glyma.19G219800 | 0.25 | 1.26 | 0.20 |
| 733 | Glyma.03G222800 | Glyma.19G219900 | 0.08 | 0.15 | 0.53 |
| 734 | Glyma.03G222900 | Glyma.19G220000 | 0.03 | 0.1 | 0.30 |
| 735 | Glyma.03G223000 | Glyma.19G220200 | 0 | 0.13 | 0.00 |
| 736 | Glyma.03G223100 | Glyma.19G220300 | 0.01 | 0.09 | 0.11 |
| 737 | Glyma.03G223200 | Glyma.19G220400 | 0.03 | 0.1 | 0.30 |
| 738 | Glyma.03G223300 | Glyma.19G220500 | 0.24 | 0.34 | 0.71 |
| 739 | Glyma.03G223400 | Glyma.19G220600 | 0.06 | 0.27 | 0.22 |
| 740 | Glyma.03G223500 | Glyma.19G220700 | 0.14 | 0.24 | 0.58 |
| 741 | Glyma.03G223600 | Glyma.19G220800 | 0.05 | 0.07 | 0.71 |
| 742 | Glyma.03G223800 | Glyma.19G220900 | 0.07 | 0.17 | 0.41 |
| 743 | Glyma.03G223900 | Glyma.19G221000 | 0.02 | 0.2 | 0.10 |
| 744 | Glyma.03G224000 | Glyma.19G221100 | 0.04 | 0.16 | 0.25 |
| 745 | Glyma.03G224200 | Glyma.19G221200 | 0.03 | 0.12 | 0.25 |
| 746 | Glyma.03G224400 | Glyma.19G221300 | 0.01 | 0.08 | 0.13 |
| 747 | Glyma.03G224500 | Glyma.19G221400 | 0 | 0.09 | 0.00 |
| 748 | Glyma.03G224600 | Glyma.19G221600 | 0.01 | 0.08 | 0.13 |
| 749 | Glyma.03G224700 | Glyma.19G221700 | 0.06 | 0.12 | 0.50 |
| 750 | Glyma.03G224800 | Glyma.19G221900 | 0.02 | 0.13 | 0.15 |
| 751 | Glyma.03G225000 | Glyma.19G222000 | 0.05 | 0.1 | 0.50 |
| 752 | Glyma.03G225100 | Glyma.19G222100 | 0.11 | 0.14 | 0.79 |
| 753 | Glyma.03G225200 | Glyma.19G222200 | 0.02 | 0.11 | 0.18 |
| 754 | Glyma.03G225300 | Glyma.19G222300 | 0.07 | 0.19 | 0.37 |
| 755 | Glyma.03G225400 | Glyma.19G222400 | 0.04 | 0.1 | 0.40 |
| 756 | Glyma.03G225500 | Glyma.19G222500 | 0.04 | 0.11 | 0.36 |
| 757 | Glyma.03G225600 | Glyma.19G222600 | 0.06 | 0.13 | 0.46 |
| 758 | Glyma.03G225800 | Glyma.19G222700 | 0.01 | 0.09 | 0.11 |
| 759 | Glyma.03G225900 | Glyma.19G222900 | 0.02 | 0.11 | 0.18 |
| 760 | Glyma.03G226000 | Glyma.19G223000 | 0.02 | 0.11 | 0.18 |
| 761 | Glyma.03G226100 | Glyma.19G223100 | 0.02 | 0.08 | 0.25 |
| 762 | Glyma.03G226200 | Glyma.19G223200 | 0.01 | 0.08 | 0.13 |
| 763 | Glyma.03G226300 | Glyma.19G223300 | 0.02 | 0.12 | 0.17 |

| | | | | | |
|---|---|---|---|---|---|
| 764 | Glyma.03G226400 | Glyma.19G223400 | 0.02 | 0.07 | 0.29 |
| 765 | Glyma.03G226500 | Glyma.19G223500 | 0.02 | 0.06 | 0.33 |
| 766 | Glyma.03G226600 | Glyma.19G223600 | 0.03 | 0.12 | 0.25 |
| 767 | Glyma.03G226700 | Glyma.19G223700 | 0.02 | 0.1 | 0.20 |
| 768 | Glyma.03G226900 | Glyma.19G223800 | 0.05 | 0.09 | 0.56 |
| 769 | Glyma.03G227000 | Glyma.19G223900 | 0.04 | 0.13 | 0.31 |
| 770 | Glyma.03G227100 | Glyma.19G224000 | 0.02 | 0.1 | 0.20 |
| 771 | Glyma.03G227200 | Glyma.19G224100 | 0.07 | 0.08 | 0.88 |
| 772 | Glyma.03G227300 | Glyma.19G224200 | 0.1 | 0.14 | 0.71 |
| 773 | Glyma.03G227400 | Glyma.19G224300 | 0.02 | 0.19 | 0.11 |
| 774 | Glyma.03G227500 | Glyma.19G224400 | 0.01 | 0.12 | 0.08 |
| 775 | Glyma.03G227600 | Glyma.19G224500 | 0.01 | 0.07 | 0.14 |
| 776 | Glyma.03G227700 | Glyma.19G224600 | 0.02 | 0.12 | 0.17 |
| 777 | Glyma.03G227800 | Glyma.19G224700 | 0.05 | 0.07 | 0.71 |
| 778 | Glyma.03G228000 | Glyma.19G225200 | 0.03 | 0.09 | 0.33 |
| 779 | Glyma.03G228100 | Glyma.19G225400 | 0.02 | 0.13 | 0.15 |
| 780 | Glyma.03G228200 | Glyma.19G225500 | 0.02 | 0.1 | 0.20 |
| 781 | Glyma.03G228300 | Glyma.19G225600 | 0.03 | 0.22 | 0.14 |
| 782 | Glyma.03G228400 | Glyma.19G225700 | 0.08 | 0.16 | 0.50 |
| 783 | Glyma.03G228500 | Glyma.19G225800 | 0.03 | 0.13 | 0.23 |
| 784 | Glyma.03G228600 | Glyma.19G225900 | 0.05 | 0.1 | 0.50 |
| 785 | Glyma.03G228700 | Glyma.19G226000 | 0.06 | 0.1 | 0.60 |
| 786 | Glyma.03G228900 | Glyma.19G226100 | 0.08 | 0.19 | 0.42 |
| 787 | Glyma.03G229000 | Glyma.19G226200 | 0.01 | 0.12 | 0.08 |
| 788 | Glyma.03G229100 | Glyma.19G226300 | 0.15 | 0.46 | 0.33 |
| 789 | Glyma.03G229200 | Glyma.19G226400 | 0 | 0.11 | 0.00 |
| 790 | Glyma.03G229300 | Glyma.19G226600 | 0.02 | 0.08 | 0.25 |
| 791 | Glyma.03G229400 | Glyma.19G226700 | 0.03 | 0.13 | 0.23 |
| 792 | Glyma.03G229500 | Glyma.19G226800 | 0.05 | 0.19 | 0.26 |
| 793 | Glyma.03G229600 | Glyma.19G226900 | 0.03 | 0.14 | 0.21 |
| 794 | Glyma.03G229700 | Glyma.19G227000 | 0.02 | 0.11 | 0.18 |
| 795 | Glyma.03G230300 | Glyma.19G227400 | 0.16 | 0.24 | 0.67 |
| 796 | Glyma.03G230800 | Glyma.19G228000 | 0.07 | 0.12 | 0.58 |
| 797 | Glyma.03G230900 | Glyma.19G228100 | 0.11 | 0.2 | 0.55 |
| 798 | Glyma.03G231000 | Glyma.19G228200 | 0.04 | 0.09 | 0.44 |
| 799 | Glyma.03G231100 | Glyma.19G228300 | 0.02 | 0.14 | 0.14 |
| 800 | Glyma.03G231200 | Glyma.19G228400 | 0.02 | 0.13 | 0.15 |
| 801 | Glyma.03G231300 | Glyma.19G228500 | 0.05 | 0.11 | 0.45 |
| 802 | Glyma.03G231500 | Glyma.19G228600 | 0.11 | 0.23 | 0.48 |
| 803 | Glyma.03G231700 | Glyma.19G228700 | 0.04 | 0.1 | 0.40 |
| 804 | Glyma.03G231800 | Glyma.19G228800 | 0 | 0.13 | 0.00 |
| 805 | Glyma.03G231900 | Glyma.19G228900 | 0 | 0.1 | 0.00 |
| 806 | Glyma.03G232000 | Glyma.19G229000 | 0.05 | 0.15 | 0.33 |
| 807 | Glyma.03G232100 | Glyma.19G229100 | 0.05 | 0.15 | 0.33 |
| 808 | Glyma.03G232200 | Glyma.19G229200 | 0.04 | 0.08 | 0.50 |

| | | | | | |
|---|---|---|---|---|---|
| 809 | Glyma.03G232300 | Glyma.19G229300 | 0.06 | 0.23 | 0.26 |
| 810 | Glyma.03G232400 | Glyma.19G229400 | 0.06 | 0.14 | 0.43 |
| 811 | Glyma.03G232700 | Glyma.19G229700 | 0.02 | 0.13 | 0.15 |
| 812 | Glyma.03G232800 | Glyma.19G229800 | 0.01 | 0.11 | 0.09 |
| 813 | Glyma.03G232900 | Glyma.19G229900 | 0.06 | 0.26 | 0.23 |
| 814 | Glyma.03G233000 | Glyma.19G230000 | 0.03 | 0.09 | 0.33 |
| 815 | Glyma.03G233100 | Glyma.19G230100 | 0.03 | 0.13 | 0.23 |
| 816 | Glyma.03G233300 | Glyma.19G230200 | 0.01 | 0.1 | 0.10 |
| 817 | Glyma.03G233400 | Glyma.19G230500 | 0.02 | 0.1 | 0.20 |
| 818 | Glyma.03G233500 | Glyma.19G230600 | 0.01 | 0.08 | 0.13 |
| 819 | Glyma.03G233600 | Glyma.19G230700 | 0.05 | 0.09 | 0.56 |
| 820 | Glyma.03G233700 | Glyma.19G230900 | 0.04 | 0.12 | 0.33 |
| 821 | Glyma.03G233800 | Glyma.19G231000 | 0.04 | 0.09 | 0.44 |
| 822 | Glyma.03G234000 | Glyma.19G231200 | 0.02 | 0.13 | 0.15 |
| 823 | Glyma.03G234100 | Glyma.19G231500 | 0.04 | 0.2 | 0.20 |
| 824 | Glyma.03G234200 | Glyma.19G231600 | 0.02 | 0.13 | 0.15 |
| 825 | Glyma.03G234300 | Glyma.19G231700 | 0.09 | 0.41 | 0.22 |
| 826 | Glyma.03G234600 | Glyma.19G232100 | 0.01 | 0.07 | 0.14 |
| 827 | Glyma.03G234700 | Glyma.19G232300 | 0.01 | 0.06 | 0.17 |
| 828 | Glyma.03G234800 | Glyma.19G232400 | 0.18 | 0.23 | 0.78 |
| 829 | Glyma.03G234900 | Glyma.19G232500 | 0.03 | 0.11 | 0.27 |
| 830 | Glyma.03G235000 | Glyma.19G232700 | 0.02 | 0.14 | 0.14 |
| 831 | Glyma.03G235100 | Glyma.19G232800 | 0.03 | 0.05 | 0.60 |
| 832 | Glyma.03G235200 | Glyma.19G232900 | 0.07 | 0.12 | 0.58 |
| 833 | Glyma.03G235300 | Glyma.19G233000 | 0.01 | 0.12 | 0.08 |
| 834 | Glyma.03G235400 | Glyma.19G233100 | 0.03 | 0.04 | 0.75 |
| 835 | Glyma.03G235500 | Glyma.19G233300 | 0.01 | 0.09 | 0.11 |
| 836 | Glyma.03G235600 | Glyma.19G233400 | 0.03 | 0.15 | 0.20 |
| 837 | Glyma.03G235700 | Glyma.19G233500 | 0.18 | 0.21 | 0.86 |
| 838 | Glyma.03G235800 | Glyma.19G233600 | 0.03 | 0.14 | 0.21 |
| 839 | Glyma.03G236000 | Glyma.19G233700 | 0.09 | 0.12 | 0.75 |
| 840 | Glyma.03G236100 | Glyma.19G233800 | 0.03 | 0.12 | 0.25 |
| 841 | Glyma.03G236300 | Glyma.19G233900 | 0.01 | 0.12 | 0.08 |
| 842 | Glyma.03G236400 | Glyma.19G234000 | 0.04 | 0.12 | 0.33 |
| 843 | Glyma.03G236500 | Glyma.19G234400 | 0.02 | 0.07 | 0.29 |
| 844 | Glyma.03G236600 | Glyma.19G234500 | 0.04 | 0.12 | 0.33 |
| 845 | Glyma.03G236900 | Glyma.19G234600 | 0.02 | 0.09 | 0.22 |
| 846 | Glyma.03G237000 | Glyma.19G234700 | 0.13 | 0.33 | 0.39 |
| 847 | Glyma.03G237100 | Glyma.19G234800 | 0.03 | 0.14 | 0.21 |
| 848 | Glyma.03G237200 | Glyma.19G234900 | 0.06 | 0.09 | 0.67 |
| 849 | Glyma.03G237400 | Glyma.19G235000 | 0.05 | 0.23 | 0.22 |
| 850 | Glyma.03G237500 | Glyma.19G235100 | 0.17 | 0.28 | 0.61 |
| 851 | Glyma.03G237600 | Glyma.19G235200 | 0.02 | 0.09 | 0.22 |
| 852 | Glyma.03G237700 | Glyma.19G235300 | 0.06 | 0.12 | 0.50 |
| 853 | Glyma.03G237800 | Glyma.19G235400 | 0.03 | 0.07 | 0.43 |

| | | | | | |
|---|---|---|---|---|---|
| 854 | Glyma.03G238100 | Glyma.19G235500 | 0.02 | 0.1 | 0.20 |
| 855 | Glyma.03G238200 | Glyma.19G235600 | 0.1 | 0.22 | 0.45 |
| 856 | Glyma.03G238300 | Glyma.19G235800 | 0.03 | 0.14 | 0.21 |
| 857 | Glyma.03G238400 | Glyma.19G235900 | 0.01 | 0.1 | 0.10 |
| 858 | Glyma.03G238600 | Glyma.19G236000 | 0.04 | 0.08 | 0.50 |
| 859 | Glyma.03G238700 | Glyma.19G236100 | 0.04 | 0.16 | 0.25 |
| 860 | Glyma.03G239000 | Glyma.19G236200 | 0.03 | 0.16 | 0.19 |
| 861 | Glyma.03G239200 | Glyma.19G236300 | 0.08 | 0.14 | 0.57 |
| 862 | Glyma.03G239400 | Glyma.19G236400 | 0 | 0.09 | 0.00 |
| 863 | Glyma.03G239600 | Glyma.19G236500 | 0.01 | 0.22 | 0.05 |
| 864 | Glyma.03G239700 | Glyma.19G236600 | 0.03 | 0.18 | 0.17 |
| 865 | Glyma.03G239800 | Glyma.19G236700 | 0.08 | 0.2 | 0.40 |
| 866 | Glyma.03G239900 | Glyma.19G236800 | 0.04 | 0.19 | 0.21 |
| 867 | Glyma.03G240000 | Glyma.19G236900 | 0.04 | 0.14 | 0.29 |
| 868 | Glyma.03G240100 | Glyma.19G237000 | 0.02 | 0.05 | 0.40 |
| 869 | Glyma.03G240200 | Glyma.19G237200 | 0.05 | 0.15 | 0.33 |
| 870 | Glyma.03G240300 | Glyma.19G237300 | 0.02 | 0.09 | 0.22 |
| 871 | Glyma.03G240400 | Glyma.19G237500 | 0.16 | 0.21 | 0.76 |
| 872 | Glyma.03G240500 | Glyma.19G237600 | 0.09 | 0.12 | 0.75 |
| 873 | Glyma.03G240600 | Glyma.19G237700 | 0.02 | 0.1 | 0.20 |
| 874 | Glyma.03G240700 | Glyma.19G238000 | 0.03 | 0.12 | 0.25 |
| 875 | Glyma.03G240800 | Glyma.19G238100 | 0.06 | 0.19 | 0.32 |
| 876 | Glyma.03G240900 | Glyma.19G238400 | 0.06 | 0.21 | 0.29 |
| 877 | Glyma.03G241000 | Glyma.19G238500 | 0.07 | 0.13 | 0.54 |
| 878 | Glyma.03G241100 | Glyma.19G238600 | 0 | 0.28 | 0.00 |
| 879 | Glyma.03G241400 | Glyma.19G238900 | 0.06 | 0.37 | 0.16 |
| 880 | Glyma.03G241700 | Glyma.19G239100 | 0.04 | 0.13 | 0.31 |
| 881 | Glyma.03G241800 | Glyma.19G239200 | 0 | 0.13 | 0.00 |
| 882 | Glyma.03G241900 | Glyma.19G239300 | 0.05 | 0.19 | 0.26 |
| 883 | Glyma.03G242000 | Glyma.19G239400 | 0.01 | 0.11 | 0.09 |
| 884 | Glyma.03G242100 | Glyma.19G239500 | 0.04 | 0.11 | 0.36 |
| 885 | Glyma.03G242300 | Glyma.19G239700 | 0.04 | 0.09 | 0.44 |
| 886 | Glyma.03G242400 | Glyma.19G239800 | 0.06 | 0.1 | 0.60 |
| 887 | Glyma.03G242500 | Glyma.19G240000 | 0.04 | 0.14 | 0.29 |
| 888 | Glyma.03G242600 | Glyma.19G240100 | 0.19 | 0.17 | 1.12 |
| 889 | Glyma.03G242700 | Glyma.19G240200 | 0.02 | 0.17 | 0.12 |
| 890 | Glyma.03G242800 | Glyma.19G240300 | 0.03 | 0.13 | 0.23 |
| 891 | Glyma.03G242900 | Glyma.19G240400 | 0.03 | 0.13 | 0.23 |
| 892 | Glyma.03G243000 | Glyma.19G240500 | 0.02 | 0.06 | 0.33 |
| 893 | Glyma.03G243100 | Glyma.19G240600 | 0.02 | 0.12 | 0.17 |
| 894 | Glyma.03G243400 | Glyma.19G240700 | 0.02 | 0.09 | 0.22 |
| 895 | Glyma.03G243500 | Glyma.19G241000 | 0.02 | 0.09 | 0.22 |
| 896 | Glyma.03G243600 | Glyma.19G241100 | 0.04 | 0.11 | 0.36 |
| 897 | Glyma.03G243700 | Glyma.19G241200 | 0.03 | 0.09 | 0.33 |
| 898 | Glyma.03G243800 | Glyma.19G241300 | 0.01 | 0.08 | 0.13 |

| | | | | | |
|---|---|---|---|---|---|
| 899 | Glyma.03G244000 | Glyma.19G241400 | 0.03 | 0.1 | 0.30 |
| 900 | Glyma.03G244200 | Glyma.19G241700 | 0.03 | 0.22 | 0.14 |
| 901 | Glyma.03G244300 | Glyma.19G241900 | 0.42 | 1.45 | 0.29 |
| 902 | Glyma.03G244700 | Glyma.19G242200 | 0.01 | 0.09 | 0.11 |
| 903 | Glyma.03G244800 | Glyma.19G242300 | 0.02 | 0.14 | 0.14 |
| 904 | Glyma.03G245000 | Glyma.19G242400 | 0.02 | 0.08 | 0.25 |
| 905 | Glyma.03G245200 | Glyma.19G242600 | 0.04 | 0.15 | 0.27 |
| 906 | Glyma.03G245300 | Glyma.19G242700 | 0.01 | 0.1 | 0.10 |
| 907 | Glyma.03G245400 | Glyma.19G242800 | 0.01 | 0.1 | 0.10 |
| 908 | Glyma.03G245500 | Glyma.19G242900 | 0.05 | 0.16 | 0.31 |
| 909 | Glyma.03G245600 | Glyma.19G243100 | 0.06 | 0.13 | 0.46 |
| 910 | Glyma.03G245700 | Glyma.19G243200 | 0.01 | 0.13 | 0.08 |
| 911 | Glyma.03G245800 | Glyma.19G243300 | 0.02 | 0.12 | 0.17 |
| 912 | Glyma.03G245900 | Glyma.19G243400 | 0.03 | 0.07 | 0.43 |
| 913 | Glyma.03G246000 | Glyma.19G243500 | 0.06 | 0.14 | 0.43 |
| 914 | Glyma.03G246100 | Glyma.19G243600 | 0.03 | 0.12 | 0.25 |
| 915 | Glyma.03G246200 | Glyma.19G243700 | 0.04 | 0.13 | 0.31 |
| 916 | Glyma.03G246300 | Glyma.19G243800 | 0.02 | 0.22 | 0.09 |
| 917 | Glyma.03G246500 | Glyma.19G243900 | 0.1 | 0.15 | 0.67 |
| 918 | Glyma.03G246600 | Glyma.19G244100 | 0.04 | 0.16 | 0.25 |
| 919 | Glyma.03G246700 | Glyma.19G244200 | 0.19 | 1.3 | 0.15 |
| 920 | Glyma.03G246800 | Glyma.19G244300 | 0.01 | 0.18 | 0.06 |
| 921 | Glyma.03G246900 | Glyma.19G244500 | 0.04 | 0.07 | 0.57 |
| 922 | Glyma.03G247000 | Glyma.19G244600 | 0 | 0.08 | 0.00 |
| 923 | Glyma.03G247100 | Glyma.19G244800 | 0.04 | 0.08 | 0.50 |
| 924 | Glyma.03G247200 | Glyma.19G244900 | 0.09 | 0.25 | 0.36 |
| 925 | Glyma.03G247300 | Glyma.19G245100 | 0.02 | 0.12 | 0.17 |
| 926 | Glyma.03G247400 | Glyma.19G245200 | 0.04 | 0.14 | 0.29 |
| 927 | Glyma.03G247500 | Glyma.19G245400 | 0.26 | 1.57 | 0.17 |
| 928 | Glyma.03G247600 | Glyma.19G245700 | 0.02 | 0.14 | 0.14 |
| 929 | Glyma.03G247800 | Glyma.19G245800 | 0.04 | 0.17 | 0.24 |
| 930 | Glyma.03G247900 | Glyma.19G245900 | 0.06 | 0.21 | 0.29 |
| 931 | Glyma.03G248000 | Glyma.19G246000 | 0.02 | 0.07 | 0.29 |
| 932 | Glyma.03G248100 | Glyma.19G246100 | 0.06 | 0.18 | 0.33 |
| 933 | Glyma.03G248200 | Glyma.19G246200 | 0.01 | 0.05 | 0.20 |
| 934 | Glyma.03G248300 | Glyma.19G246300 | 0.16 | 0.23 | 0.70 |
| 935 | Glyma.03G248800 | Glyma.19G246400 | 0.03 | 0.09 | 0.33 |
| 936 | Glyma.03G248900 | Glyma.19G246500 | 0.05 | 0.15 | 0.33 |
| 937 | Glyma.03G249000 | Glyma.19G246600 | 0.1 | 0.27 | 0.37 |
| 938 | Glyma.03G249100 | Glyma.19G246700 | 0.08 | 0.2 | 0.40 |
| 939 | Glyma.03G249300 | Glyma.19G246800 | 0.02 | 0.09 | 0.22 |
| 940 | Glyma.03G249500 | Glyma.19G247000 | 0.02 | 0.09 | 0.22 |
| 941 | Glyma.03G249600 | Glyma.19G247100 | 0.01 | 0.14 | 0.07 |
| 942 | Glyma.03G249700 | Glyma.19G247300 | 0.07 | 0.24 | 0.29 |
| 943 | Glyma.03G249800 | Glyma.19G247400 | 0.06 | 0.13 | 0.46 |

| | | | | | |
|---|---|---|---|---|---|
| 944 | Glyma.03G249900 | Glyma.19G247500 | 0.02 | 0.12 | 0.17 |
| 945 | Glyma.03G250000 | Glyma.19G247600 | 0.06 | 0.1 | 0.60 |
| 946 | Glyma.03G250100 | Glyma.19G247700 | 0.03 | 0.11 | 0.27 |
| 947 | Glyma.03G250200 | Glyma.19G247800 | 0.06 | 0.16 | 0.38 |
| 948 | Glyma.03G250300 | Glyma.19G247900 | 0.11 | 0.26 | 0.42 |
| 949 | Glyma.03G250400 | Glyma.19G248000 | 0.02 | 0.15 | 0.13 |
| 950 | Glyma.03G250600 | Glyma.19G248100 | 0.05 | 0.08 | 0.63 |
| 951 | Glyma.03G250700 | Glyma.19G248200 | 0.01 | 0.07 | 0.14 |
| 952 | Glyma.03G250900 | Glyma.19G248300 | 0.03 | 0.15 | 0.20 |
| 953 | Glyma.03G251100 | Glyma.19G248400 | 0.01 | 0.06 | 0.17 |
| 954 | Glyma.03G251200 | Glyma.19G248500 | 0.04 | 0.21 | 0.19 |
| 955 | Glyma.03G251300 | Glyma.19G248600 | 0.01 | 0.05 | 0.20 |
| 956 | Glyma.03G251500 | Glyma.19G248700 | 0.02 | 0.13 | 0.15 |
| 957 | Glyma.03G251600 | Glyma.19G249000 | 0.03 | 0.16 | 0.19 |
| 958 | Glyma.03G251700 | Glyma.19G249100 | 0.04 | 0.12 | 0.33 |
| 959 | Glyma.03G251800 | Glyma.19G249200 | 0.03 | 0.13 | 0.23 |
| 960 | Glyma.03G251900 | Glyma.19G249300 | 0.03 | 0.1 | 0.30 |
| 961 | Glyma.03G252000 | Glyma.19G249400 | 0.01 | 0.09 | 0.11 |
| 962 | Glyma.03G252100 | Glyma.19G249500 | 0.04 | 0.08 | 0.50 |
| 963 | Glyma.03G252200 | Glyma.19G249800 | 0.03 | 0.12 | 0.25 |
| 964 | Glyma.03G252300 | Glyma.19G249900 | 0.01 | 0.09 | 0.11 |
| 965 | Glyma.03G252500 | Glyma.19G250000 | 0.02 | 0.1 | 0.20 |
| 966 | Glyma.03G252800 | Glyma.19G250100 | 0.22 | 2.16 | 0.10 |
| 967 | Glyma.03G252900 | Glyma.19G250500 | 0.15 | 0.21 | 0.71 |
| 968 | Glyma.03G253000 | Glyma.19G250600 | 0.01 | 0.06 | 0.17 |
| 969 | Glyma.03G253100 | Glyma.19G250700 | 0.03 | 0.22 | 0.14 |
| 970 | Glyma.03G253300 | Glyma.19G250800 | 0.01 | 0.12 | 0.08 |
| 971 | Glyma.03G253400 | Glyma.19G250900 | 0.03 | 0.13 | 0.23 |
| 972 | Glyma.03G253500 | Glyma.19G251000 | 0.05 | 0.15 | 0.33 |
| 973 | Glyma.03G253600 | Glyma.19G251100 | 0.04 | 0.14 | 0.29 |
| 974 | Glyma.03G253700 | Glyma.19G251200 | 0.08 | 0.13 | 0.62 |
| 975 | Glyma.03G253800 | Glyma.19G251300 | 0.04 | 0.12 | 0.33 |
| 976 | Glyma.03G254000 | Glyma.19G251400 | 0.01 | 0.09 | 0.11 |
| 977 | Glyma.03G254100 | Glyma.19G251600 | 0.04 | 0.06 | 0.67 |
| 978 | Glyma.03G254200 | Glyma.19G251700 | 0.2 | 0.38 | 0.53 |
| 979 | Glyma.03G254300 | Glyma.19G251900 | 0.13 | 0.28 | 0.46 |
| 980 | Glyma.03G254400 | Glyma.19G252000 | 0.07 | 0.22 | 0.32 |
| 981 | Glyma.03G254500 | Glyma.19G252100 | 0.03 | 0.08 | 0.38 |
| 982 | Glyma.03G254600 | Glyma.19G252300 | 0.07 | 0.14 | 0.50 |
| 983 | Glyma.03G254700 | Glyma.19G252400 | 0 | 0.03 | 0.00 |
| 984 | Glyma.03G254800 | Glyma.19G252500 | 0.01 | 0.11 | 0.09 |
| 985 | Glyma.03G255000 | Glyma.19G252600 | 0.02 | 0.09 | 0.22 |
| 986 | Glyma.03G255100 | Glyma.19G252700 | 0.02 | 0.09 | 0.22 |
| 987 | Glyma.03G255200 | Glyma.19G252800 | 0.07 | 0.09 | 0.78 |
| 988 | Glyma.03G255300 | Glyma.19G252900 | 0.15 | 0.47 | 0.32 |

| | | | | | |
|---|---|---|---|---|---|
| 989 | Glyma.03G255400 | Glyma.19G253000 | 0 | 0.13 | 0.00 |
| 990 | Glyma.03G255500 | Glyma.19G253100 | 0.08 | 0.11 | 0.73 |
| 991 | Glyma.03G255600 | Glyma.19G253200 | 0.03 | 0.13 | 0.23 |
| 992 | Glyma.03G255800 | Glyma.19G253300 | 0 | 0.1 | 0.00 |
| 993 | Glyma.03G255900 | Glyma.19G253600 | 0.03 | 0.08 | 0.38 |
| 994 | Glyma.03G256000 | Glyma.19G253800 | 0.07 | 0.1 | 0.70 |
| 995 | Glyma.03G256100 | Glyma.19G253900 | 0.01 | 0.09 | 0.11 |
| 996 | Glyma.03G256200 | Glyma.19G254000 | 0.01 | 0.08 | 0.13 |
| 997 | Glyma.03G256300 | Glyma.19G254200 | 0.03 | 0.13 | 0.23 |
| 998 | Glyma.03G256400 | Glyma.19G254300 | 0.32 | 0.54 | 0.59 |
| 999 | Glyma.03G256500 | Glyma.19G254500 | 0.08 | 0.27 | 0.30 |
| 1000 | Glyma.03G256600 | Glyma.19G254600 | 0.25 | 1.32 | 0.19 |
| 1001 | Glyma.03G256700 | Glyma.19G254800 | 0.04 | 0.1 | 0.40 |
| 1002 | Glyma.03G256800 | Glyma.19G255000 | 0.05 | 0.11 | 0.45 |
| 1003 | Glyma.03G256900 | Glyma.19G255100 | 0.03 | 0.1 | 0.30 |
| 1004 | Glyma.03G257000 | Glyma.19G255300 | 0.03 | 0.09 | 0.33 |
| 1005 | Glyma.03G257300 | Glyma.19G255600 | 0.03 | 0.15 | 0.20 |
| 1006 | Glyma.03G257500 | Glyma.19G255800 | 0.01 | 0.11 | 0.09 |
| 1007 | Glyma.03G257600 | Glyma.19G256100 | 0.02 | 0.09 | 0.22 |
| 1008 | Glyma.03G257700 | Glyma.19G256200 | 0.02 | 0.14 | 0.14 |
| 1009 | Glyma.03G257800 | Glyma.19G256300 | 0.03 | 0.11 | 0.27 |
| 1010 | Glyma.03G257900 | Glyma.19G256400 | 0.06 | 0.3 | 0.20 |
| 1011 | Glyma.03G258000 | Glyma.19G256500 | 0.04 | 0.17 | 0.24 |
| 1012 | Glyma.03G258100 | Glyma.19G256700 | 0.09 | 0.22 | 0.41 |
| 1013 | Glyma.03G258200 | Glyma.19G256800 | 0.02 | 0.16 | 0.13 |
| 1014 | Glyma.03G258400 | Glyma.19G257000 | 0.05 | 0.17 | 0.29 |
| 1015 | Glyma.03G258500 | Glyma.19G257100 | 0.08 | 0.3 | 0.27 |
| 1016 | Glyma.03G258600 | Glyma.19G257200 | 0.02 | 0.04 | 0.50 |
| 1017 | Glyma.03G258800 | Glyma.19G257500 | 0.02 | 0.11 | 0.18 |
| 1018 | Glyma.03G258900 | Glyma.19G257600 | 0.01 | 0.09 | 0.11 |
| 1019 | Glyma.03G259000 | Glyma.19G257700 | 0.02 | 0.1 | 0.20 |
| 1020 | Glyma.03G259100 | Glyma.19G257800 | 0.02 | 0.13 | 0.15 |
| 1021 | Glyma.03G259200 | Glyma.19G258000 | 0.01 | 0.15 | 0.07 |
| 1022 | Glyma.03G259300 | Glyma.19G258100 | 0.04 | 0.14 | 0.29 |
| 1023 | Glyma.03G259400 | Glyma.19G258300 | 0.07 | 0.19 | 0.37 |
| 1024 | Glyma.03G259500 | Glyma.19G258400 | 0.16 | 0.32 | 0.50 |
| 1025 | Glyma.03G259600 | Glyma.19G258500 | 0.03 | 0.17 | 0.18 |
| 1026 | Glyma.03G259700 | Glyma.19G258800 | 0.32 | 1.57 | 0.20 |
| 1027 | Glyma.03G259800 | Glyma.19G258900 | 0.02 | 0.11 | 0.18 |
| 1028 | Glyma.03G260100 | Glyma.19G259000 | 0.09 | 0.1 | 0.90 |
| 1029 | Glyma.03G260400 | Glyma.19G259100 | 0.43 | 0.74 | 0.58 |
| 1030 | Glyma.03G260500 | Glyma.19G259200 | 0.01 | 0.08 | 0.13 |
| 1031 | Glyma.03G260600 | Glyma.19G259300 | 0.03 | 0.15 | 0.20 |
| 1032 | Glyma.03G260700 | Glyma.19G259600 | 0.11 | 0.22 | 0.50 |
| 1033 | Glyma.03G260900 | Glyma.19G259800 | 0.06 | 0.23 | 0.26 |

| | | | | | |
|---|---|---|---|---|---|
| 1034 | Glyma.03G261000 | Glyma.19G260000 | 0.02 | 0.08 | 0.25 |
| 1035 | Glyma.03G261100 | Glyma.19G260100 | 0.01 | 0.1 | 0.10 |
| 1036 | Glyma.03G261200 | Glyma.19G260300 | 0.07 | 0.16 | 0.44 |
| 1037 | Glyma.03G261300 | Glyma.19G260400 | 0.05 | 0.09 | 0.56 |
| 1038 | Glyma.03G261400 | Glyma.19G260500 | 0.03 | 0.17 | 0.18 |
| 1039 | Glyma.03G261500 | Glyma.19G260600 | 0.22 | 0.35 | 0.63 |
| 1040 | Glyma.03G261800 | Glyma.19G260900 | 0.04 | 0.07 | 0.57 |
| 1041 | Glyma.03G261900 | Glyma.19G261000 | 0.05 | 0.14 | 0.36 |
| 1042 | Glyma.03G262000 | Glyma.19G261100 | 0.01 | 0.19 | 0.05 |
| 1043 | Glyma.03G262100 | Glyma.19G261200 | 0.02 | 0.09 | 0.22 |
| 1044 | Glyma.03G262200 | Glyma.19G261300 | 0.07 | 0.21 | 0.33 |
| 1045 | Glyma.03G262300 | Glyma.19G261400 | 0.07 | 0.31 | 0.23 |
| 1046 | Glyma.03G262400 | Glyma.19G261500 | 0.03 | 0.12 | 0.25 |
| 1047 | Glyma.03G262700 | Glyma.19G261700 | 0.04 | 0.13 | 0.31 |
| 1048 | Glyma.03G262800 | Glyma.19G261800 | 0 | 0.09 | 0.00 |
| 1049 | Glyma.03G262900 | Glyma.19G262000 | 0.04 | 0.13 | 0.31 |
| 1050 | Glyma.03G263100 | Glyma.19G262200 | 0.04 | 0.1 | 0.40 |
| 1051 | Glyma.03G263400 | Glyma.19G262400 | 0.03 | 0.05 | 0.60 |
| 1052 | Glyma.03G263500 | Glyma.19G262500 | 0.02 | 0.12 | 0.17 |
| 1053 | Glyma.03G263600 | Glyma.19G262600 | 0.04 | 0.23 | 0.17 |
| 1054 | Glyma.03G263700 | Glyma.19G262700 | 0.09 | 0.17 | 0.53 |
| 1055 | Glyma.03G263900 | Glyma.19G262800 | 0.11 | 0.27 | 0.41 |
| 1056 | Glyma.03G264100 | Glyma.19G263100 | 0.04 | 0.13 | 0.31 |
| 1057 | Glyma.03G264200 | Glyma.19G263200 | 0.02 | 0.09 | 0.22 |
| 1058 | Glyma.03G264300 | Glyma.19G263300 | 0.03 | 0.1 | 0.30 |
| 1059 | Glyma.03G264400 | Glyma.19G263400 | 0.02 | 0.08 | 0.25 |
| 1060 | Glyma.03G264600 | Glyma.19G263600 | 0.02 | 0.08 | 0.25 |
