# Supplementary material for: Characterization of the FAD2 Gene Family in Soybean Reveals the Limitations of Gel-Based TILLING in Genes with High Copy Number
Source: Front Plant Sci. 2017 Mar 13;8:324. doi: 10.3389/fpls.2017.00324 (PMC5346563; doi:10.3389/fpls.2017.00324)
Supplement: Table S2 — Levels of the five major fatty acids in the seed oil of the screened mutants. The five fatty acid levels in seed oil of the four screened mutants and the wild type Forrest in the M3 (2012) and M4 (2013) generations. Fatty acid levels were averaged for n segregating the M3 and M4 lines for each mutation. Averages and standard deviations are shown. [file Table2.PDF]

	Palmitic Acid (16:0)	Stearic Acid (18:0)	Oleic Acid (18:1)	Linoleic Acid (18:2)	Linolenic Acid (18:3)
Forrest WT	**10.3±30.59**	**3.25±0.15**	**18.89±2.34**	**53.71±3.28**	**6.89±0.79**
FM3-1274	9.6	3.6	47.2	28.0	4.5
	7.8	3.1	44.7	23.2	4.7
	10.8	3.0	25.8	44.4	7.2
	9.3	3.0	38.2	36.2	5.2
	8.6	2.7	41.8	25.8	5.0
Average	9.2±1.13	3.1±0.34	**39.5±8.4**	31.5±8.68	5.3±1.1
FM4-1274	8.23	2.43	32.87	45.07	8.68
	10.01	5.06	28.19	42.66	5.37
	7.96	3.35	26.82	40.66	7.08
	10.14	2.92	31.05	46.42	6.31
Average	9.08±1.14	3.44±1.14	**29.73±2.73**	43.7±2.55	6.86±1.4
FM3-1284	3.81	4.10	28.80	37.71	9.48
	8.04	3.264	29.693	28.78	8.448
	10.99	1.67	44.43	35.82	3.79
	9.95	1.4	33.2	50.43	3.64
Average	8.2±3.17	2.61±1.29	**34.03±7.19**	38.18±9.02	6.34±3.06
FM4-1284	11.364	4.539	43.308	30.654	3.35
	11.31	3.9	45.97	32.08	3.65
	11.93	3.8	39.49	39.27	5.49
	10.31	3.18	42.5	33.02	5.48
Average	11.22±0.67	3.85±0.55	**42.81±2.66**	33.75±3.8	4.49±1.15
FM3-812	10.659	3.243	21.868	51.089	5.782
	9.438	3.706	26.881	46.932	6.233
	11.432	3.381	24.214	44.992	7.653
	13.195	3.857	21.132	48.643	4.575
Average	11.18±1.57	3.54±0.28	**23.52±2.59**	47.91±2.58	6.06±1.27
FM4-812	9.92	3.01	30.51	40.8	7.73
	9.775	4.613	25.724	46.936	6.948
	9.38	4.7	30.059	41.991	6.684
	9.503	4.114	25.006	46.804	6.891
Average	9.64±0.24	4.1±0.77	**27.82±2.86**	44.13±3.19	7.06±0.45
