# Supplementary material for: Characterization of the FAD2 Gene Family in Soybean Reveals the Limitations of Gel-Based TILLING in Genes with High Copy Number
Source: Front Plant Sci. 2017 Mar 13;8:324. doi: 10.3389/fpls.2017.00324 (PMC5346563; doi:10.3389/fpls.2017.00324)
Supplement: Table S3 — Levels of the five major fatty acids the in seed oil of the re-screened high oleic mutants. The five fatty acid levels in the seed oil of the eight re-screened mutants and the wild type Forrest in the M2 (2011) and M3 (2012) generations. Two replicates from the M2 and M3 lines are shown for each mutant. Asterisk (*) represents the oleic acid content from the first M3 screening. Underlined are mutants presenting significant seed oleic acid changes and maintaining high oleic levels from two generations. [file Table3.PDF]

Lines	Palmitic acid		Stearic acid		Oleic acid		Linoleic acid		Linolenic acid	
	2011 (M2)	2012 (M3)	2011 (M2)	2012 (M3)	2011 (M2)	2012 (M3)	2011 (M2)	2012 (M3)	2011 (M2)	2012 (M3)
**Forrest WT**	8.76±0.09		2.05±0.25		**14.25±2.17**		47.04±4.37		8.15±1.65	
F403	8.85	8.36	2.62	2.98	18.73	21.54	47.87	36.36	6.67	5.47
	10.42	9.67	2.94	2.39	14.96	13.84	40.02	48.31	6.88	8.74
F550	7.4	10.06	1.98	2.96	12.12	11.63	31.21	46.42	5.12	13.34
	8.35	8.08	2.74	19.36	16.03	43.33	34.91	29.21	5.86	
F445	7.34	11.71	2.09	4.87	39.7	27.82*	27.18	40.19	4.18	5.33
	7.05	ND	2.7	ND	25.08	ND	36.55	ND	4.14	ND
**F782**	9.48	10.29	3.96	2.41	26.24	11.63	46.59	41.9	4.35	11.53
	7.48	10.19	3.43	4.89	22.98	33.2	38.64	35.26	4.32	6.48
F784	8.05	7.84	3.74	4.37	23.33	35.1	36.22	34.57	3.72	4.04
	9.23	8.05	3.1	3.68	19.21	23.38	45.04	43.13	5.14	6.51
F924	7.61	21.09	2.54	5.27	25.51	29.58*	38.15	32.6	4.44	3.76
	6.34	ND	2.51	ND	18.66	ND	32.8	ND	4.4	ND
F1085	ND	10.15	ND	2.41	ND	16.3	ND	44.65	ND	7.99
	ND	16.59	ND	19.9	ND	33.24	ND	30.25	ND	
**F1235**	10.01	7.91	5.06	2.24	28.19	20.88	42.66	36.15	5.37	7.17
	7.95	ND	4.93	ND	23.6	50.86	42.76	49.13	5.59	ND
