# Supplementary material for: Characterization of the FAD2 Gene Family in Soybean Reveals the Limitations of Gel-Based TILLING in Genes with High Copy Number
Source: Front Plant Sci. 2017 Mar 13;8:324. doi: 10.3389/fpls.2017.00324 (PMC5346563; doi:10.3389/fpls.2017.00324)
Supplement: Table S4 — Primers used for TILLING and sequencing. [file Table4.PDF]

Gene	Gene model	Primers	Primer Sequences	Products (bps)	Target	Purpose
FAD2-1A	Glyma10g42470	FAD2-1A-Fw	CACATTCAGCAAAACAACTGA	1272	Exon 1	Sequencing & Tilling
		FAD2-1A-Rv	TGTACTAATACATGACAAAAC			
		FAD2-1A-Fw-Pro	ATGACATGTAATTGAATTTT	595	Promoter	Sequencing
		FAD2-1A-Rv-Pro	TAAACAAGTTCAGTTGAGTT			
FAD2-1B	Glyma20g24530	FAD2-1B-Fw	GTATTAGACATTCAGCAACAAC	1273	Exon 1	Sequencing & Tilling
		FAD2-1B-Rv	TTAAGTGATAAGTGACAAAAC			
		FAD2-1B-Fw-Pro	AAATTTAACTACGTAGCATC	570	Promoter	Sequencing
		FAD2-1B-Rv-Pro	TGTTGCTGAATGTCTAATAC			
